# Computational Study of a Model System of Enzyme-Mediated [4+2] Cycloaddition Reaction

**DOI:** 10.1371/journal.pone.0119984

**Published:** 2015-04-08

**Authors:** Evgeniy G. Gordeev, Valentine P. Ananikov

**Affiliations:** Zelinsky Institute of Organic Chemistry, Russian Academy of Sciences, Leninsky Prospekt 47, Moscow, Russia; Instituto de Tecnologica Química e Biológica, UNL, PORTUGAL

## Abstract

A possible mechanistic pathway related to an enzyme-catalyzed [4+2] cycloaddition reac-tion was studied by theoretical calculations at density functional (B3LYP, O3LYP, M062X) and semiempirical levels (PM6-DH2, PM6) performed on a model system. The calculations were carried out for the key [4+2] cycloaddition step considering enzyme-catalyzed biosynthesis of Spinosyn A in a model reaction, where a reliable example of a biological Diels-Alder reaction was reported experimentally. In the present study it was demonstrated that the [4+2] cycloaddition reaction may benefit from moving along the energetically balanced reaction coordinate, which enabled the catalytic rate enhancement of the [4+2] cycloaddition pathway involving a single transition state. Modeling of such a system with coordination of three amino acids indicated a reliable decrease of activation energy by ~18.0 kcal/mol as compared to a non-catalytic transformation.

## Introduction

From the practical point of view, the cycloaddition strategy is a well-recognized direct route to cyclic molecules and it is ubiquitously utilized in organic chemistry and industry for the preparation of pharmaceutical substances, drugs, biologically active molecules, new materials and molecular devices [1, 2, 3, 4]. An outstanding potential of cycloaddition reactions for eco-friendly technologies is governed by so-called “100% atom efficiency”—all the starting material is converted to product without generation of any wastes [5, 6, 7]. Rapid development of Diels-Alder [4+2] cycloaddition chemistry has explored new opportunities and fascinating applications leading to elaboration of efficient practical procedures and resulting in high product yields. An amazing feature revealed for cycloaddition reactions is a wide substrate scope and high tolerance to various functional groups present in the substrate [1, 2, 3, 4, 8, 9, 10, 11, 12, 13]. Ultimately, this approach can provide an efficient chemical methodology to access a diverse range of cyclic products of key-importance. However, the stumbling block in cycloaddition chemistry is the development of a new generation of catalysts to achieve high stereoselectivity and enantioselectivity under mild conditions (preferably at room temperature). Since enzymatic catalysis has not yet been employed in the area, many potentially very useful cycloaddition reactions are currently carried out at high temperatures of 100–200°C and require additional chemical reagents to speed up the transformation (to be removed as wastes at the product purification step) [8, 9, 10, 11, 12, 13].

In spite of the undisputable significance and outstanding areas of practical applications, the opportunity of enzyme-catalyzed cycloaddition reactions remains a challenging question. The unique feature of the concerted cycloaddition pathway, proposed for the Diels-Alder reaction, is a single-step mechanism without involvement of transient species (concerted pathway via a single transition state was shown for Diels-Alder reaction [1, 2, 3, 4, 8, 9, 10, 11, 12]; stepwise ring formation is also known, but it is out of scope of the present study). Remarkably, an enzyme may catalyze a chemical transformation involving only one transition state (without splitting to a multi-stage route and several smaller energy barriers) [14]. The mechanistic picture of cycloaddition reactions at the enzymatic level was discussed in terms of protein motions and dynamics, activation by H-bonds, influence of protein microenvironment or a combination of these and other factors [15, 16, 17, 18, 19, 20, 21, 22, 23, 24, 25, 26]. The enzymatic mechanism of concerted cycloaddition was called enigmatic [27], while an efficient and selective Diels-Alderase was for many years “a holy grail” dream in enzymatic catalysis [28].

Biochemical equivalents of [4+2] cycloaddition reactions have been proposed as the key steps in the biosynthesis of many natural products and possible involvement of enzymes (“Diels-Alderase”) have been debated recently [15, 16, 17, 18, 29, 30]. Some enzymes are known to mediate a cyclohexene ring formation involving the [4+2] cycloaddition: solanapyrone synthase [31], lovastatin nonaketide synthase [32], riboflavin synthase [33], and macrophomate synthase [34]. Recently, the molecular structures of certain enzymes catalyzing the cycloaddition reactions have been determined [35, 36]. An elegant proof of concept was demonstrated by computational design of an enzyme catalyst for carrying out an intermolecular Diels-Alder reaction [37].

**Fig 1:**
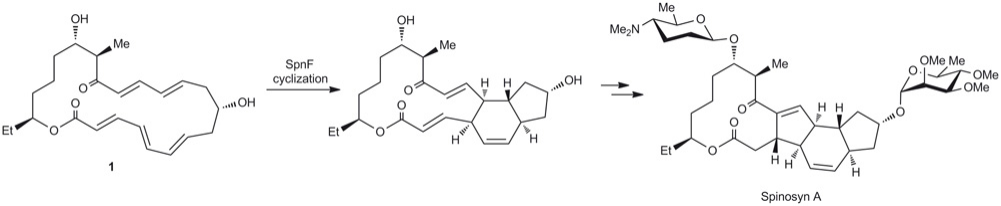
SpnF-mediated cyclization. SpnF-mediated cyclization leading to cyclohexene ring in the biosynthesis of Spinosyn A.

An important finding in the field was reported in the study of enzyme-catalyzed [4+2] cycloaddition in the biosynthesis of Spinosyn A (Fig. 1) [38]. The study has identified a cyclase (SpnF), that solely catalyzed the [4+2] cycloaddition reaction and resulted in the construction of a cyclohexene ring. The kinetic measurements have shown as large as 500-fold rate enhancement in a specific acceleration of the six-membered ring formation reaction [38]. Spinosyn A is a component of effective eco-friendly insecticides; it has a low toxicity towards vertebrata and is used in commercially available preparations [38].

SpnF was reported to be the first enzyme experimentally verified for the specific acceleration of the concerted [4+2] cycloaddition reaction [14, 38]. An important point of the model system is governed by intramolecular transformation of the cyclic substrate (**1**). Promoting the cyclization by reducing activation entropy and adopting a favorable mutual orientation of the reacting fragments may be feasible for an intermolecular reaction [29, 30, 39, 40], whereas it may not be expected to play a significant role in the intramolecular transformation of chemically preorganized macrocyclic substrate **1** catalyzed by SpnF (Fig. 1). Thus, the cyclohexene formation step in the synthesis of Spinosyn A is an excellent model for computational studies.

In the present article we explored possible pathways of the [4+2] cycloaddition by computational methods using the synthesis of Spinosyn A as a model. A new approach is proposed to describe the cycloaddition reaction in the catalyst active center involving a single transition state.

## Results and Discussion

### Analysis of [4+2] cycloaddition reaction for the studied macrocyclic substrate

**Fig 2:**
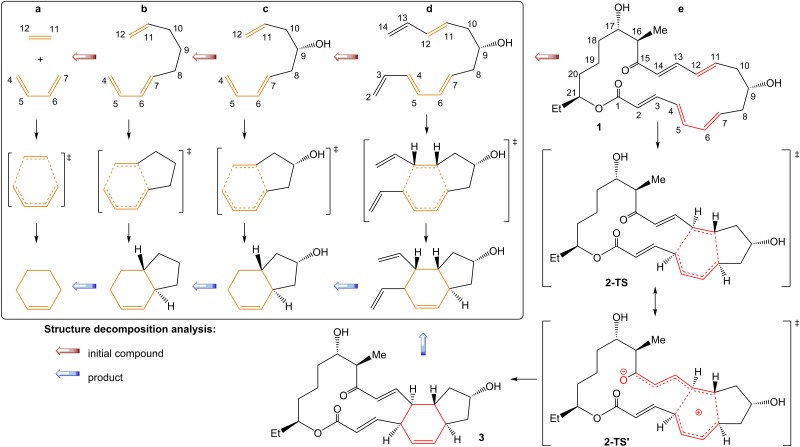
Structural decomposition analysis of the cycloaddition reaction. Structural decomposition analysis of the cycloaddition reaction involved in the biosynthesis of Spinosyn A into principal components (the atom numbering was the same as in compound **1** for comparative purpose).

The classical Diels-Alder reaction involves cycloaddition of butadiene and ethylene units and results in a cyclohexene ring via a single transition state (Fig. 2a) [1, 2, 3, 4, 8, 9, 10, 11, 12]. In the biosynthesis of Spinosyn A, several structural changes were introduced to this [4+2] reaction core, and an important question is to what extent these changes may affect the reactivity of the ring construction. Possible contributions of the following known factors have been considered in the present study: 1) influence of substituents in the substrate; 2) polarization of the substrate; and 3) influence of hydrogen bonding.

#### Influence of substituents

First, we carried out a chemical structure decomposition analysis by identifying principal structural fragments and revealing their influence on energetic parameters. The following key-stages were involved in the decomposition analysis (Fig. 2): a) the reference [4+2] cycloaddition reaction; b) attachment of an intramolecular-(CH_2_)_3_- linkage; c) attachment of a hydroxyl group in the linkage; d) elongation of the conjugated chain; e) attachment of a carbonyl group and macrocyclic linkage. In fact, the structure decomposition follows an increasing complexity of assembly of Spinosyn A over the Diels-Alder reaction core in a step-by-step manner. Full geometry optimization of initial structures, transition states and products were performed for each of the reactions (Fig. 2a— 2e). The concerted transition states were successfully located for all the cases studied (see S1 Fig; S2 Fig; S3 Fig; S4 Fig; S1 Table and S1 File for structures and geometry parameters). The increase of molecular complexity led to a more pronounced asynchrony of the C-C bonds formation. The result is in a good agreement with the earlier DFT calculations of the [4+2] transition states [41, 42, 43, 44, 45, 46, 83].

The calculated activation barriers and reaction energies at the B3LYP level showed only minor changes in spite of significant structural variations (Table 1). A rather small difference of 1.5 kcal/mol was found for reactions **a** and **e**. The stepwise assembly of the target substrate maintained the activation barriers in a narrow range of ΔE^≠^ = 23.6–26.1 kcal/mol for the [4+2] cycloaddition reactions they are involved in. The calculated ΔH energy surface showed the same trend (Table 1). Thus, the decomposition analysis demonstrated that the considered structural changes neither led to a dramatic decrease of the activation barrier in the [4+2] cycloaddition in the biosynthesis of Spinosyn A, nor could account for carrying out the reaction under mild room temperature conditions. The calculated ΔG free energy surface showed an expected decrease in the activation barrier and reaction energy due to an appearance of the first (*cf*. **a** and **b**) and second (*cf*. **d** and **e**) intramolecular linkages. This effect is well-known and reflects lowering of the reaction entropy [39, 40]. In overall, the calculated energy parameters agree well with other [4+2] cycloaddition reactions involving 1,3-diene and alkene units as well as with an experimental study of reaction **a** [42, 43, 44, 45, 46].

**Table 1:** Calculated activation and reaction energies for the cycloaddition reactions.

	Reactions				
	(a)	(b)	(c)	(d)	(e)
**ΔE** ^**≠**^	24.5	26.1	26.1	23.6	22.9
**ΔE**	-32.8	-29.7	-29.7	-14.0	-9.3
**ΔH** ^**≠**^	25.6	25.6	25.7	22.8	22.0
**ΔH**	-28.2	-27.2	-27.3	-12.2	-7.5
**ΔG** ^**≠**^	38.9	30.3	30.2	27.3	25.4
**ΔG**	-15.5	-21.9	-22.0	-7.2	-2.9

Calculated activation and reaction energies (B3LYP/6-311+G(d) level, kcal/mol) for the cycloaddition reactions (see Fig. 2).

#### Polarization of the substrate

The second factor of interest is the degree of polarization of the dienophylic part of the substrate and charge delocalization over the conjugated chain (**2-TS** and **2-TS’**; Fig. 2) proposed as a plausible channel to overcome the transition state [14, 38]. An analysis of the charge delocalization showed insignificant alterations upon the formation of the transition state (see S2 Table; S3 Table; S4 Table). Thus, the calculations did not confirm that the zwitterionic (or significantly polarized) nature of the transition state would necessarily facilitate the reaction.

#### Influence of hydrogen bonding

The third important point is a possible influence of hydrogen bonding on the reactivity of the substrate in the cycloaddition reaction. These interactions may facilitate cycloaddition in the active site of enzymes due to modulation of energies of the HOMO/LUMO molecular orbitals by specific hydrogen bonding. Positioning of a hydrogen bond acceptor to diene (increasing the energy of HOMO) and a hydrogen bond donor to alkene (decreasing the energy of LUMO) could narrow the energy gap and lower the activation barrier. Both functional groups of amino acids and water molecules from a solvent shell may be involved in hydrogen bonding with the substrate. It was demonstrated that a coordination of glutamine or asparagine amino acids with diene and serine or tyrosine with dienophile can lower the activation barrier by 4.7 kcal/mol [37]. We verified the role of both factors and studied the influence of coordination of one, two and three amino acids, as well as interactions with water molecules. The calculations were carried out at various density functional levels (B3LYP, O3LYP, M062X) and semiempirical level (PM6) including an accurate description of H-bonding and dispersion interactions (PM6-DH2). PM6-DH2 has shown the same accuracy as sophisticated B2-PLYP-D/TZVPP and M06-2X/6-311+G(3df,2p) levels. The deviation of 0.4 kcal/mol with benchmark CCSD(T)/CBS level was re-ported [47, 48].

As shown by the density functional calculations, a coordination of Gln to the hydroxyl group has a small influence on the reaction barrier (<1 kcal/mol); on the PM6 energy surface the reaction became slightly more favored by ~3 kcal/mol (entries 1, 2; Table 2). The influence of the second amino acid slightly lowered the barrier at the B3LYP and O3LYP levels, while the opposite change was found for the M062X, PM6 and PM6-DH2 energy surfaces (entries 3, 2; Table 2). A coordination of Ser to the carbonyl group of the substrate slightly lowered the activation energy (entries 4, 3; Table 2), which was in line with proposed polarization in the substrate (Fig. 2). At the density functional level the activation barriers were decreased by 0.4–5.7 kcal/mol and by 1.3 kcal/mol at the PM6 level (Table 2). A replacement of hydrogen bonding of amino acids by explicit solvation with water molecules resulted in a similar change in the calculated parameters (ΔE^≠^_**2-TS**_ = 33.8 kcal/mol at the PM6 level).

**Table 2:** Calculated activation barriers of Spinosyn A formation.

Entry^[a]^	PM6^[b]^	PM6-DH2^[b]^	B3LYP^[c]^	O3LYP^[c]^	M062X^[c]^
1	36.6	35.2	27.8	26.4	27.2
2 (+Gln)	33.6	32.7	28.1	26.1	27.8
3 (+Gln+Ser)	36.0	35.6	27.0	23.7	30.4
4 (+Gln+2Ser)	34.7	35.9	25.1	23.3	24.7

Calculated ΔE^≠^_2-TS_ activation barriers (in kcal/mol) of the cycloaddition step involved in the biosynthesis of Spinosyn A showing effect of amino acids coordination. See S5 Fig; S6 Fig; S7 Fig; S8 Fig and S5 Table for structures and geometric parameters.

^[a]^ Coordinated amino acids: 1—none; 2—Gln; 3—Gln and Ser; 4—Gln and 2 molecules of Ser.

^[b]^ Full geometry optimization of all stationary points.

^[c]^ Single point calculations at the PM6 geometry.

The calculated influence of hydrogen bonding revealed a possible change in the activation barrier in the range of 2–5 kcal/mol in a good agreement with previous studies [28, 37]. Such a change could contribute to the reaction rate enhancement, although it can not be suggested as the only leading factor for accelerating the cycloaddition reaction under experimental conditions [14, 38].

Thus, considering the above data on the influence of substituents, polarization of the substrate and hydrogen bonding we cannot clearly identify the key factor for facilitating the reaction. Neither the substrate structure itself nor the expected H-bonding interactions can describe the enhancement of reaction rate in the [4+2] cycloaddition reaction.

### An alternative route for the studied [4+2] cycloaddition reaction

Upon considering hydrogen bonding structures we have revealed another mechanistic pathway of enzymatic catalysis of the cycloaddition reaction. The course of the Diels-Alder reaction is governed by shortening of intramolecular distances resulting in the formation of C-C bonds, which is reflected by an increase of potential energy of the system until reaching the transition state. In our finding, the energetic profile of the studied system may adopt a more favorable curve if the movement along the reaction coordinate is accompanied by stepwise hydrogen bonding. Particularly, a computational study of the stepwise process involving i) H-bonding followed by shortening of intramolecular distances; ii) the next H-bonding followed by further shortening of intramolecular distances; and iii) repeat the same sequence until complete reaction; revealed a much more favorable reaction profile.

**Fig 3:**
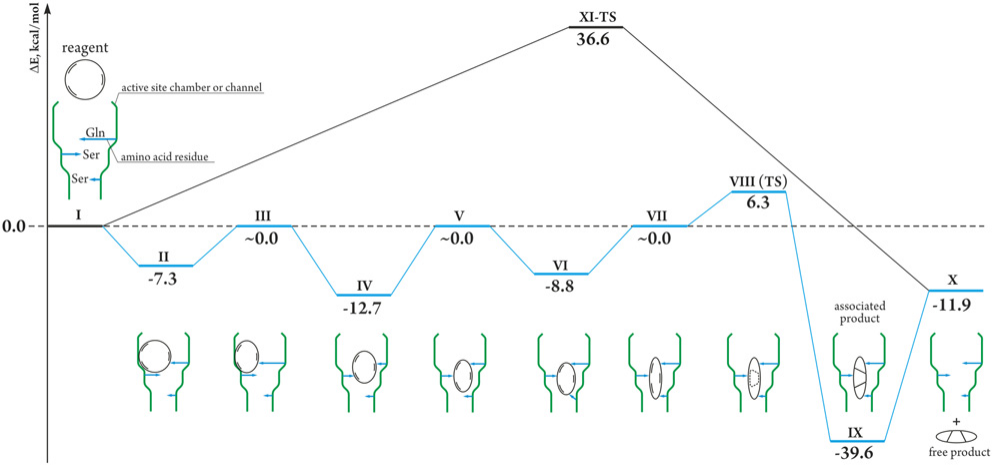
Comparison of regular and proposed cycloaddition reactions. Comparison of regular (black line) and proposed in the present study (blue line) cycloaddition reactions (calculated at the PM6 level). Coordination of amino acids and schematic substrate transformations are shown in the case of enzyme-catalyzed reaction (See S9 Fig and S10 Fig for structures and geometries).

In the studied system this kind of transformation may start with initial state **I**, where substrate **1** is located in a hydrophobic environment of the enzyme (Fig. 3). For the substrate bond distances C_7_-C_11_ = 3.217 Å and C_4_-C_12_ = 3.952 Å were calculated at the starting point (see Fig. 2 for atom numbering). A coordination of the substrate with an amino acid residue initiates an arrangement of the enzyme active center and H-bond formation with C_9_-OH was calculated to be exothermic by 7.3 kcal/mol (**II**; Fig. 3) (we have selected Gln and Ser for the model computational study as these amino acids were shown to be involved in the active center of enzymes with cycloaddition activity (see refs. [35, 36]) and the choice of these amino acids is not strictly limited, since coordination of other amino acids would lead to a similar energy gain). Upon moving in the enzyme active center the substrate experienced the influence of the protein environment and returned to the thermoneutral state on the calculated energy surface (**III**) after contraction of the macrocycle resulted in a shortening of the C_7_-C_11_ and C_4_-C_12_ bonds to 2.619 Å and 2.830 Å, respectively.

On the next step, H-bond formation between the C_17_-OH group and the second amino acid led to the energy gain of 12.7 kcal/mol (**IV**), and made further contraction of the forming bonds to C_7_-C_11_ = 2.190 Å and C_4_-C_12_ = 2.690 Å possible. At this point the calculated energy of the studied system was again around the thermoneutral point (**V**).

The third step involved H-bonding of the C_15_ = O group (**VI**) and a macrocycle ring contraction to the bond distances C_7_-C_11_ = 2.010 Å and C_4_-C_12_ = 2.610 Å. The process furnished structure **VII** with the relative energy around zero point (Fig. 3); however, geometric parameters of **VII** were significantly shifted towards the “product-like” state as compared to initial structure **I**.

Here we described the process involving movement along the reaction coordinate in *an H-bonding followed by ring contraction* manner (Fig. 3). Exactly the same energy surface was calculated when the opposite sequence of *ring contraction followed by H-bonding* was studied. Indeed, both “H-bonding/contraction” and “contraction/H-bonding” type movements along the reaction coordinate led to the same stationary points that can be unambiguously defined by the values of the C1–C6 and C4–C5 bonds lengths.

**Fig 4:**
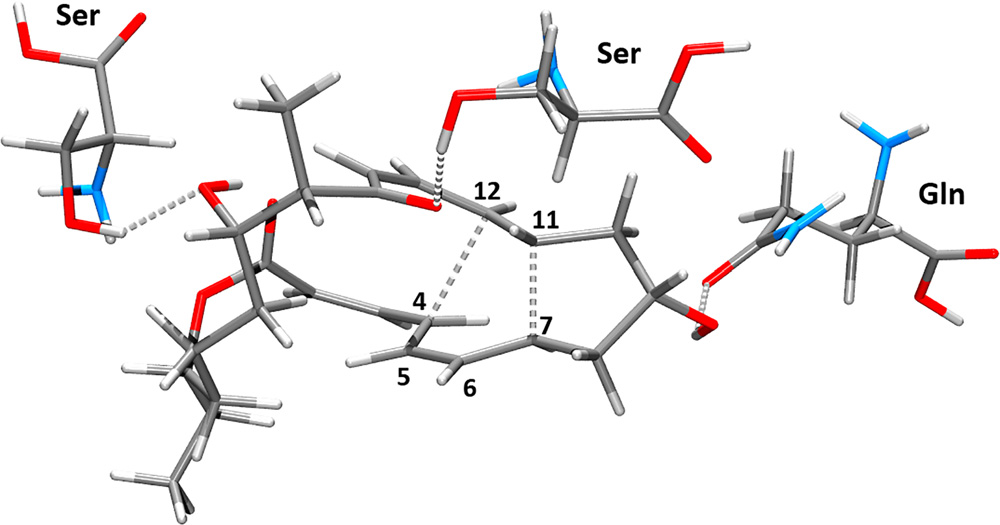
Optimized molecular structure of transition state VIII-TS.

Starting with **VII** as an initial structure, the cycloaddition reaction took place easily through the transition state **VIII-TS** (Fig. 4) and involved overcoming a small calculated activation barrier of 6.3 kcal/mol (Fig. 3). As expected, the cycloaddition reaction is exothermic due to formation of C-C bonds and due to release of internal strain of the contracted molecule.

When moving along the reaction path, the stepwise contraction of the substrate was energetically balanced by the H-bond formation. The substrate motion via the energetically balanced reaction path furnished formation of structure **VII**, which was more “product-like” as compared to **I**, while the overall energy of the system changed in the range from 0.0 to -12.7 kcal/mol. Considering the overall surface, the energy difference of 19.0 kcal/mol between the highest and lowest points (**IV** and **VIII-TS**) would contribute to the rate-determining activation parameters.

For comparison, cycloaddition of the isolated molecule of **I** requires overcoming the energy barrier as high as 36.6 kcal/mol (**XI-TS**; Fig. 3). It is important to note that a simple coordination of the same amino acids (without macrocycle contraction) did not lead to a dramatic decrease in the activation energy (Table 2 and discussion above). An important role in such a process plays an influence of the protein environment in order to govern directed macrocycle ring contraction.

Thus, the catalytic effect of the calculated pathway lowered the activation energy by 17.6 kcal/mol: from 36.6 kcal/mol for **XI-TS** to 18.0 kcal/mol for **VIII-TS**. The reaction energy remained the same for both pathways—decoordination of amino acids from **IX** results in **X** (Fig. 3). To confirm the reliability of the selected computational approach single point calculations were carried out at CCSD(T)/cc-pVDZ level. The CCSD(T) calculations have clearly indicated significant lowering of the activation energy by 13.0 kcal/mol (17.6 kcal/mol at DFT level) and a similar overall reaction energy -10.8 kcal/mol (-11.9 kcal/mol at DFT level).

## Conclusions

To summarize, in the present study we propose a new pathway for the cycloaddition reaction based on the computational study. Moving along the energetically balanced reaction profile enables enhancement of the catalytic rate of a concerted cycloaddition reaction involving a single transition state. It should be emphasized that we modeled “an ideal” system and the catalytic effect resulting in a decrease of activation energy by ~18.0 kcal/mol reflects a high efficiency value, which may be somewhat lower in the real systems due to various factors. However, the study clearly shows tremendous possibilities for enzymatic catalysis of Diels-Alder reactions and provides a mechanistic framework for the reaction involving a single transition state.

Of course, it should be noted that the absolute values of the activation barriers may significantly depend on the model system and theory level. The calculations in the present study highlighted relative changes in the activation barriers that clearly point out on higher reactivity originated from movement along the stepwise energetically balanced reaction coordinate. Such reaction pathway makes possible catalytic rate enhancement of the [4+2] cycloaddition reaction.

## Computational Methods

The calculations were performed on a small model system including interaction of the substrate with the key amino acid residues. Activation barriers and reaction energies for reactions (**a**)-(**e**) shown on Scheme 2 were calculated using the standard 6–311+G(d) basis set [49]. Full geometry optimization of reagents, transition states and products for reactions (**a**)-(**e**) was carried out using the B3LYP hybrid density functional method [50, 51, 52]. For all studied structures a normal coordinate analysis was performed to characterize the nature of the stationary points and to calculate thermodynamic properties (298.15 K and 1 atm). All transition states possess only one imaginary frequency (See S1 Table and S5 Table for the values of imaginary frequencies). The transition states were confirmed with IRC (Intrinsic Reaction Coordinate) calculations using the standard method [53, 54, 55, 56].

Modeling of the influence of hydrogen bonding on substrate reactivity in the cycloaddition reaction was performed with several approximations (Table 2). Full optimization of reagents, transition states and products was carried out at the semiempirical PM6 [57] and PM6-DH2 [58, 59] levels. PM6-DH2 was parameterized by experimental data for reproducing energy calculated by high level quantum-mechanical methods such as B2-PLYP-D/TZVPP and CCSD(T)/CBS [58, 59]. PM6-DH2 is used for improved description of H-bonding and dispersion interaction, which is especially important for biological molecules. Single point energy calculations at the PM6 geometry (Table 2) were carried out with the B3LYP [50, 51, 52], O3LYP [60] and M062X [61, 62] methods using the 6–311+G(d,p) basis set. DFT and PM6 calculations were performed using the Gaussian program [63], and PM6-DH2 calculations were performed using the MOPAC program [64]. Single point calculations with the DLPNO-CCSD(T) method [65, 66] were performed using the cc-pVDZ basis set [67, 68, 69]. The DLPNO-CCSD(T) calculations were carried out using the Orca 3.0.1 program [70].

DFT methods were proven to provide reliable and accurate results for the correct description of different types of cycloaddition reactions, in particular the [4+2] cycloaddition [61, 62, 71, 72, 73, 74, 75, 76, 77, 78, 79, 80, 81, 82] and its transannular variant, implemented in the synthesis of Spinosyn A [83]. Reaction (**a**) was investigated in several previous studies (see refs. [8, 9, 10, 11, 12, 13, 39, 40, 42, 43, 44, 45, 46, 71, 72, 73, 74, 75, 76, 77, 78, 79, 80, 81, 82]) and the calculations here are provided for comparative purpose.

For all studied structures (reactions (**a**)-(**e**) on Figs. 2, 3 and S1–S10 Figs) the atom numbering was the same as in compound **1** (Fig. 2). To mimic the influence of the enzymatic environment, for modeling of the cycloaddition step selected amino acids and their geometrical orientations were taken according to previously studied enzymatic reactions [28, 37].

## Supporting Information

S1 Fig Optimized molecular structures for reaction (a). B3LYP/6-311+G(d) optimized molecular structures of the reagent, transition state and reaction product (a) shown in Fig. 2 (column (a) in Table 1). Displacement vectors corresponding to an imaginary frequency are shown for the transition state.
(TIF)

S2 Fig Optimized molecular structures for reactions (b) and (c). B3LYP/6-311+G(d) optimized molecular structures of the reagents, transition states and reaction products (b) and (c) shown in Fig. 2 (columns (b) and (c) in Table 1). Displacement vectors corresponding to an imaginary frequency are shown for each transition state (see Fig. 2 for structures).
(TIF)

S3 Fig Optimized molecular structures for reaction (d). B3LYP/6-311+G(d) optimized molecular structures of the reagent, transition state and reaction product (d) shown in Fig. 2 (column (d) in Table 1). Displacement vectors corresponding to an imaginary frequency are shown for the transition state (see Fig. 2 for structures).
(TIF)

S4 Fig Optimized molecular structures for reaction (e). B3LYP/6-311+G(d) optimized molecular structures of the reagent, transition state and reaction product (e) shown in Fig. 2 (column (e) in Table 1). Displacement vectors corresponding to an imaginary frequency are shown for the transition state (see Fig. 2 for structures).
(TIF)

S5 Fig Molecular structures for the cycloaddition step with one amino acid. PM6 optimized molecular structures of the reagent, transition state and product of the cycloaddition step coordinated by glutamine amino acid (Entry 2 in Table 2 and S5 Table for interatomic distances). Displacement vectors corresponding to an imaginary frequency are shown for the transition state (see Fig. 2 for pericycle atomic numbers).
(TIF)

S6 Fig Molecular structures for the cycloaddition step with two amino acids. PM6 optimized molecular structures of the reagent, transition state and product of the cycloaddition step coordinated by glutamine and serine amino acids (Entry 3 in Table 2 and S5 Table for interatomic distances). Displacement vectors corresponding to an imaginary frequency are shown for the transition state (see Fig. 2 for pericycle atomic numbers).
(TIF)

S7 Fig Molecular structures for the cycloaddition step with three amino acids. PM6 optimized molecular structures of the reagent, transition state and product of the cycloaddition step coordinated by glutamine and two serine amino acids (Entry 4 in Table 2 and S5 Table for interatomic distances). Displacement vectors corresponding to an imaginary frequency are shown for the transition state (see Fig. 2 for pericycle atomic numbers).
(TIF)

S8 Fig Molecular structures for the cycloaddition step with eight water molecules. PM6 optimized molecular structures of the reagent, transition state and product of cycloaddition step coordinated by eight water molecules (see S5 Table for interatomic distances). Displacement vectors corresponding to an imaginary frequency are shown for the transition state (see Fig. 2 for pericycle atomic numbers).
(TIF)

S9 Fig PM6 optimized molecular structures (I-VI). PM6 optimized molecular structures (I-VI) of enzyme-catalyzed cycloaddition reactions of Spinoson A (see Fig. 3 for energy surface).
(TIF)

S10 Fig PM6 optimized molecular structures (VII-X). PM6 optimized molecular structures (VII-X) of enzyme-catalyzed cycloaddition reactions of Spinoson A (see Fig. 3 for energy surface).
(TIF)

S1 File XYZ structures for studied molecules. (DOC)

S1 Table Optimized geometry parameters of molecular structures 1–15. B3LYP/6-311+G(d) level (bond length in Å, angles in deg); for atom numbering see S1–S4 Figs.
(DOC)

S2 Table Mulliken atomic charges for molecules in reactions (a)-(c). PM6 and B3LYP/6-311+G(d) levels of theory (see Fig. 2 for atomic numbers).
(DOC)

S3 Table Mulliken atomic charges for molecules in reaction (d). PM6 and B3LYP/6-311+G(d) levels of theory (see Fig. 2 for atomic numbers).
(PDF)

S4 Table Mulliken atomic charges for molecules in reaction (e). PM6 and B3LYP/6-311+G(d) levels of theory (see Fig. 2 for atomic numbers).
(DOC)

S5 Table Optimized geometry parameters of molecular structures 16–27. PM6 level (bond length in Å, angles in deg); for atom numbering see Figs. S5–S8.
(DOC)
