# Supplementary figures and images for: Computational Study of a Model System of Enzyme-Mediated [4+2] Cycloaddition Reaction
Source: PLoS One. 2015 Apr 8;10(4):e0119984. doi: 10.1371/journal.pone.0119984 (PMC4390235; doi:10.1371/journal.pone.0119984)

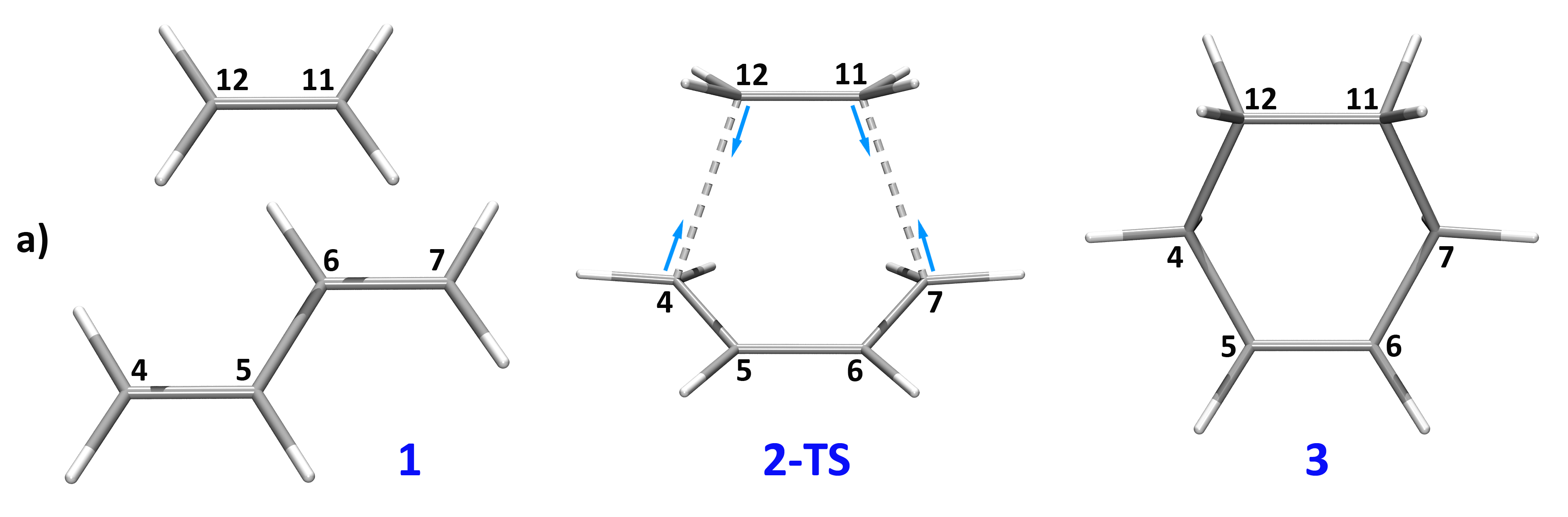

Supplement: S1 Fig — B3LYP/6-311+G(d) optimized molecular structures of the reagent, transition state and reaction product (a) shown in Fig. 2 (column (a) in Table 1). Displacement vectors corresponding to an imaginary frequency are shown for the transition state. (TIF) [file pone.0119984.s001.tif]

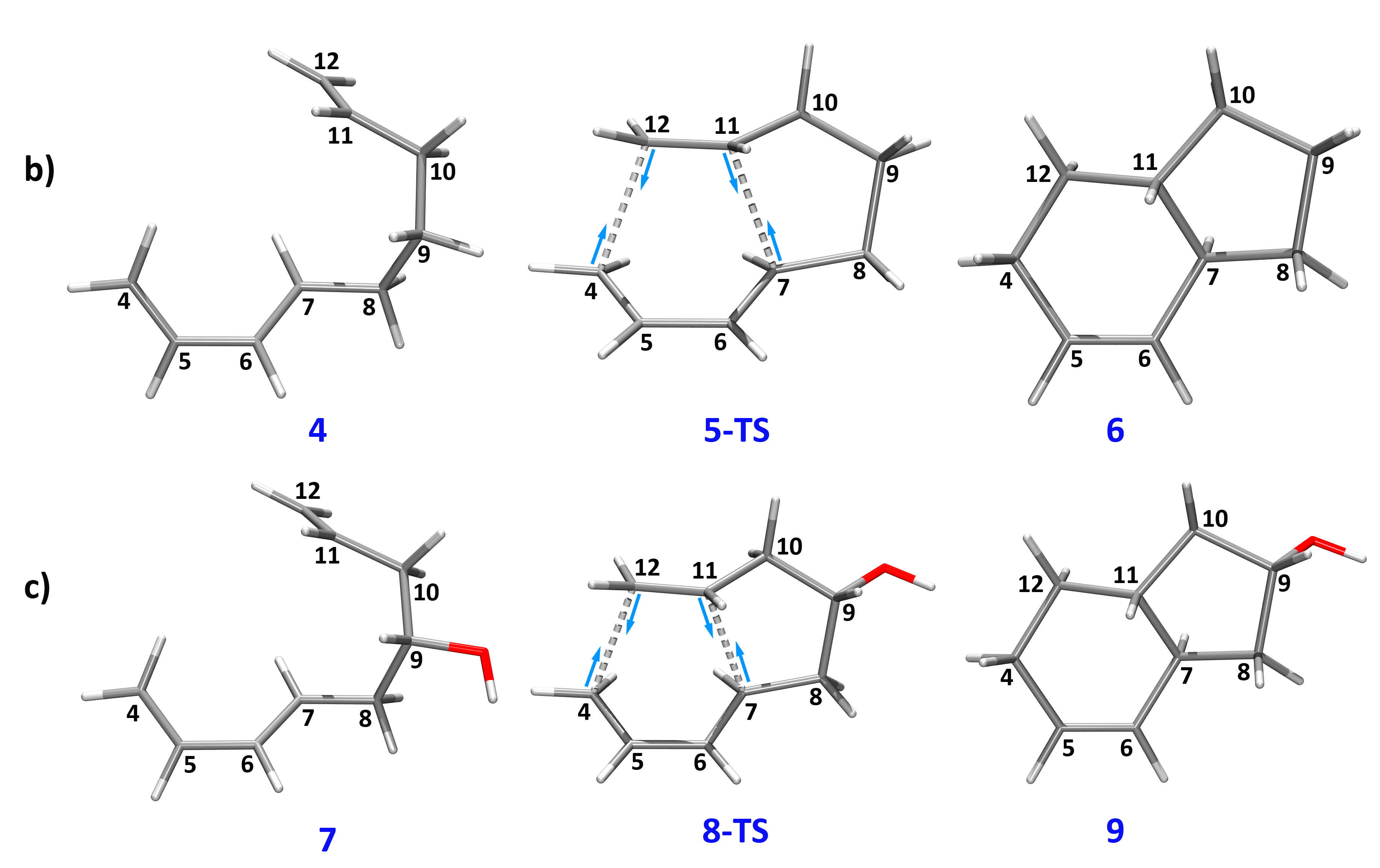

Supplement: S2 Fig — B3LYP/6-311+G(d) optimized molecular structures of the reagents, transition states and reaction products (b) and (c) shown in Fig. 2 (columns (b) and (c) in Table 1). Displacement vectors corresponding to an imaginary frequency are shown for each transition state (see Fig. 2 for structures). (TIF) [file pone.0119984.s002.tif]

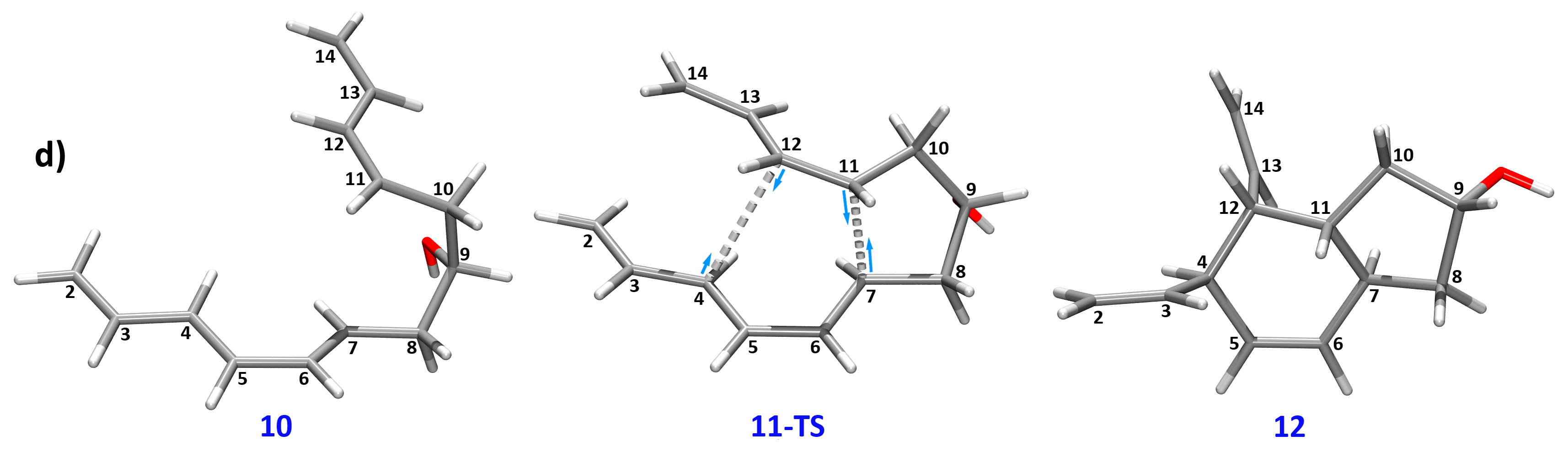

Supplement: S3 Fig — B3LYP/6-311+G(d) optimized molecular structures of the reagent, transition state and reaction product (d) shown in Fig. 2 (column (d) in Table 1). Displacement vectors corresponding to an imaginary frequency are shown for the transition state (see Fig. 2 for structures). (TIF) [file pone.0119984.s003.tif]

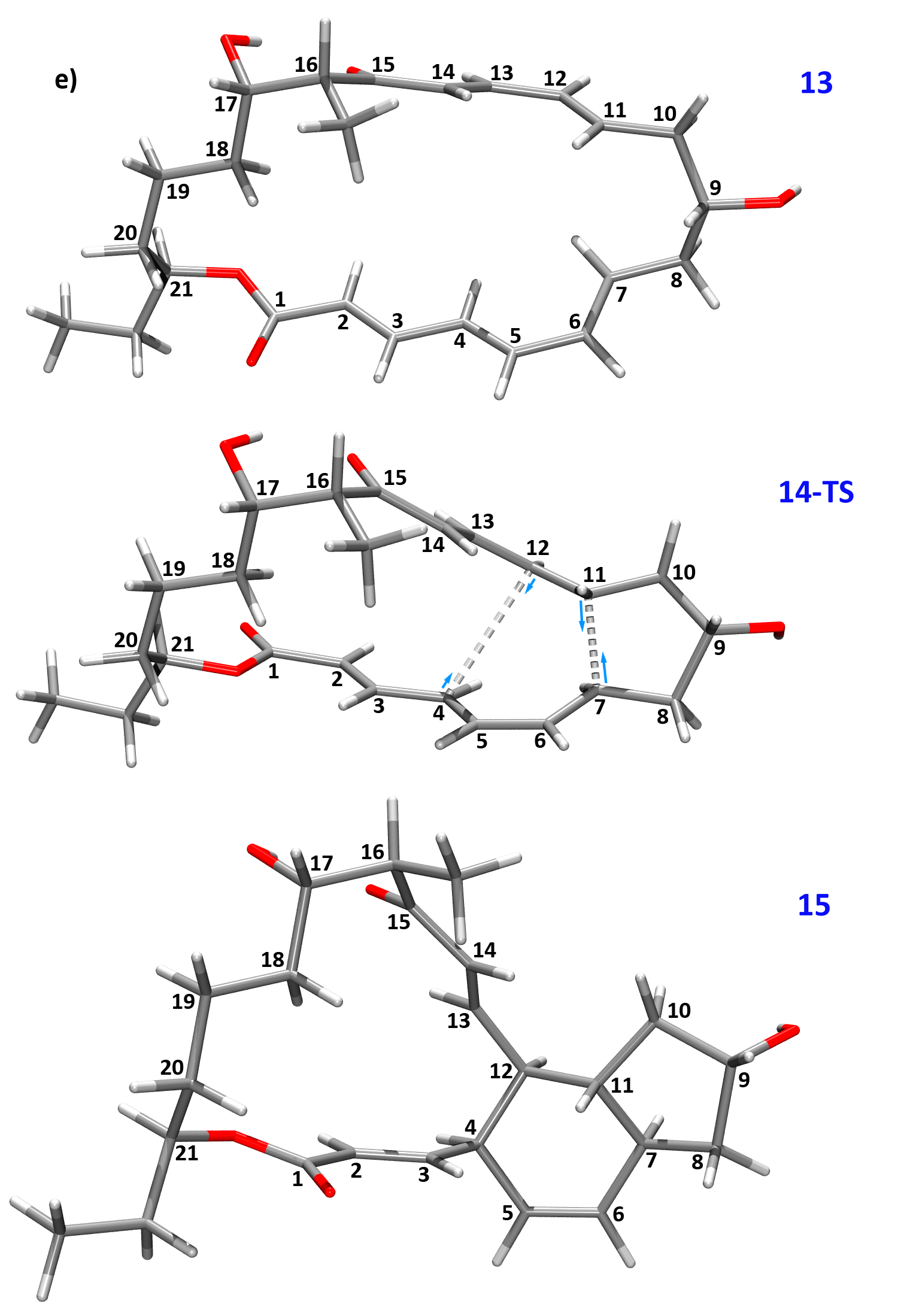

Supplement: S4 Fig — B3LYP/6-311+G(d) optimized molecular structures of the reagent, transition state and reaction product (e) shown in Fig. 2 (column (e) in Table 1). Displacement vectors corresponding to an imaginary frequency are shown for the transition state (see Fig. 2 for structures). (TIF) [file pone.0119984.s004.tif]

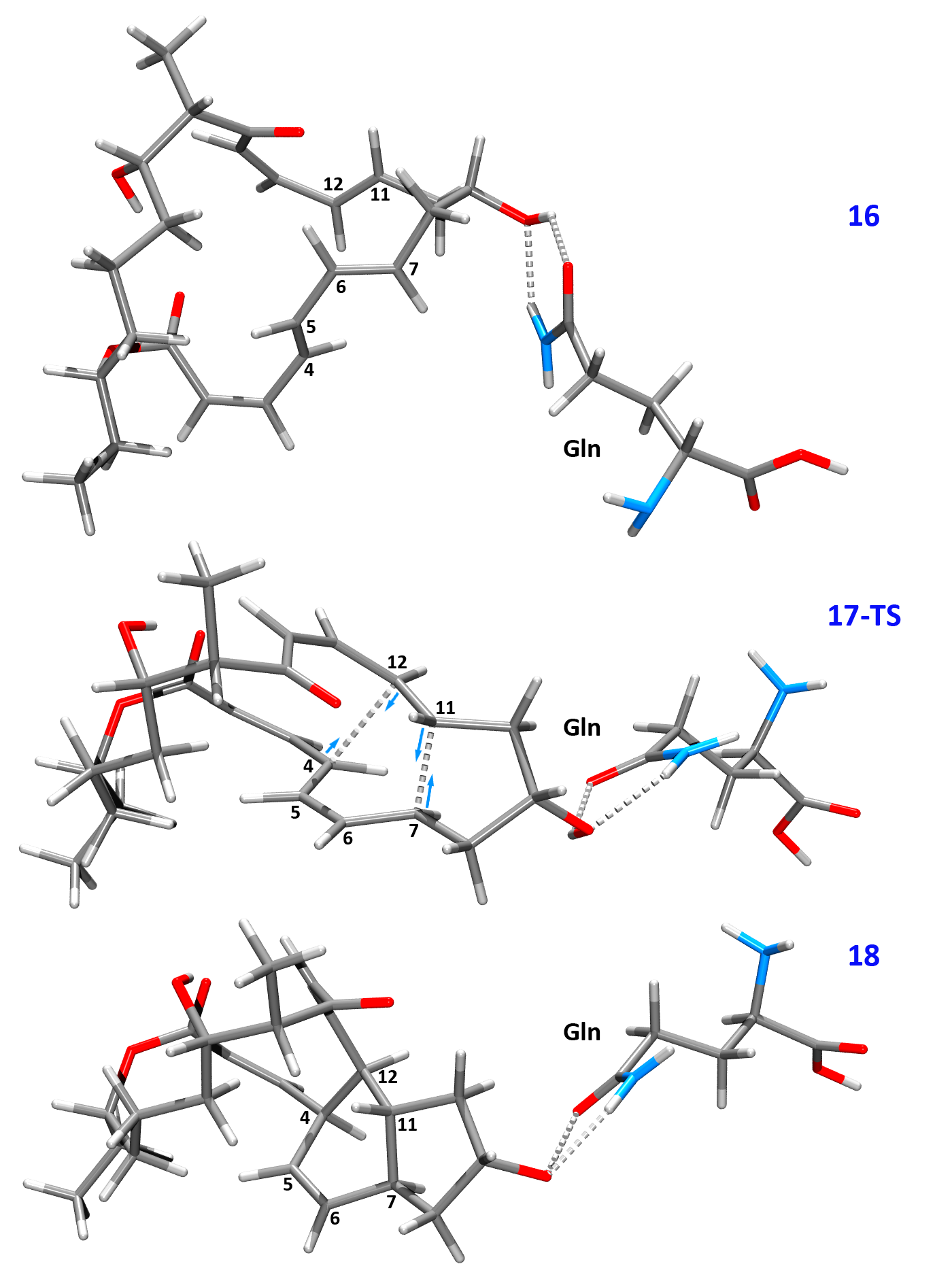

Supplement: S5 Fig — PM6 optimized molecular structures of the reagent, transition state and product of the cycloaddition step coordinated by glutamine amino acid (Entry 2 in Table 2 and S5 Table for interatomic distances). Displacement vectors corresponding to an imaginary frequency are shown for the transition state (see Fig. 2 for pericycle atomic numbers). (TIF) [file pone.0119984.s005.tif]

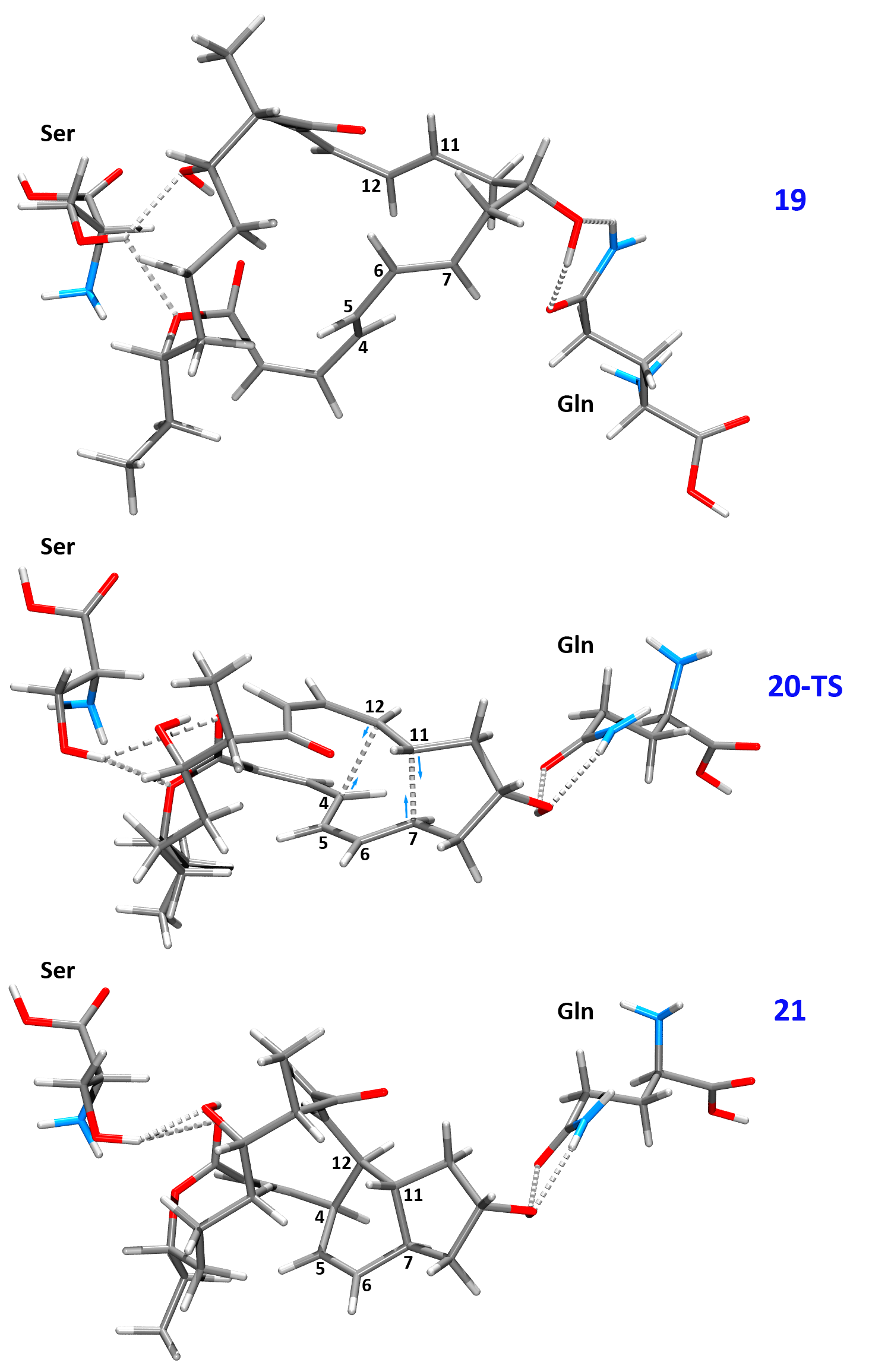

Supplement: S6 Fig — PM6 optimized molecular structures of the reagent, transition state and product of the cycloaddition step coordinated by glutamine and serine amino acids (Entry 3 in Table 2 and S5 Table for interatomic distances). Displacement vectors corresponding to an imaginary frequency are shown for the transition state (see Fig. 2 for pericycle atomic numbers). (TIF) [file pone.0119984.s006.tif]

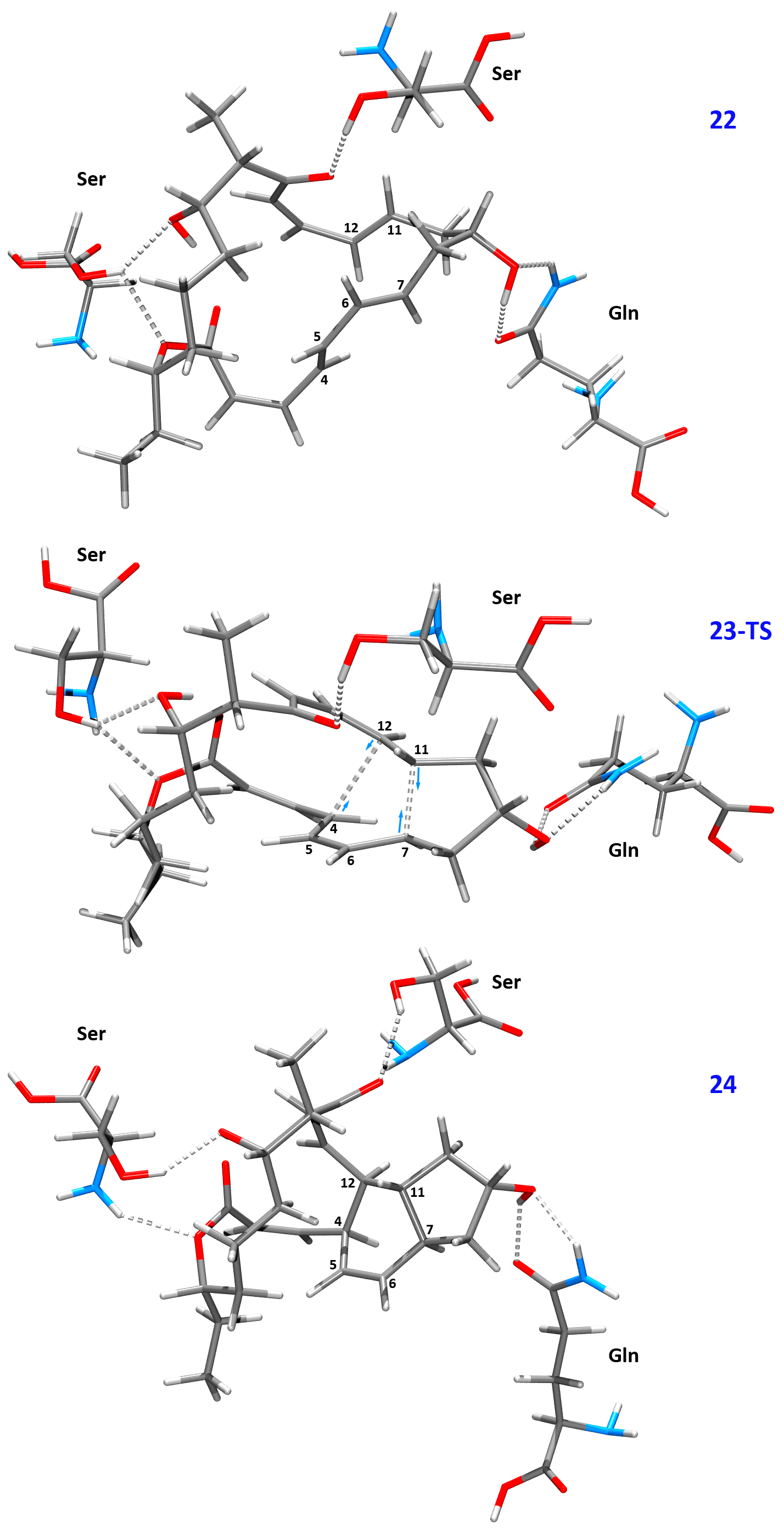

Supplement: S7 Fig — PM6 optimized molecular structures of the reagent, transition state and product of the cycloaddition step coordinated by glutamine and two serine amino acids (Entry 4 in Table 2 and S5 Table for interatomic distances). Displacement vectors corresponding to an imaginary frequency are shown for the transition state (see Fig. 2 for pericycle atomic numbers). (TIF) [file pone.0119984.s007.tif]

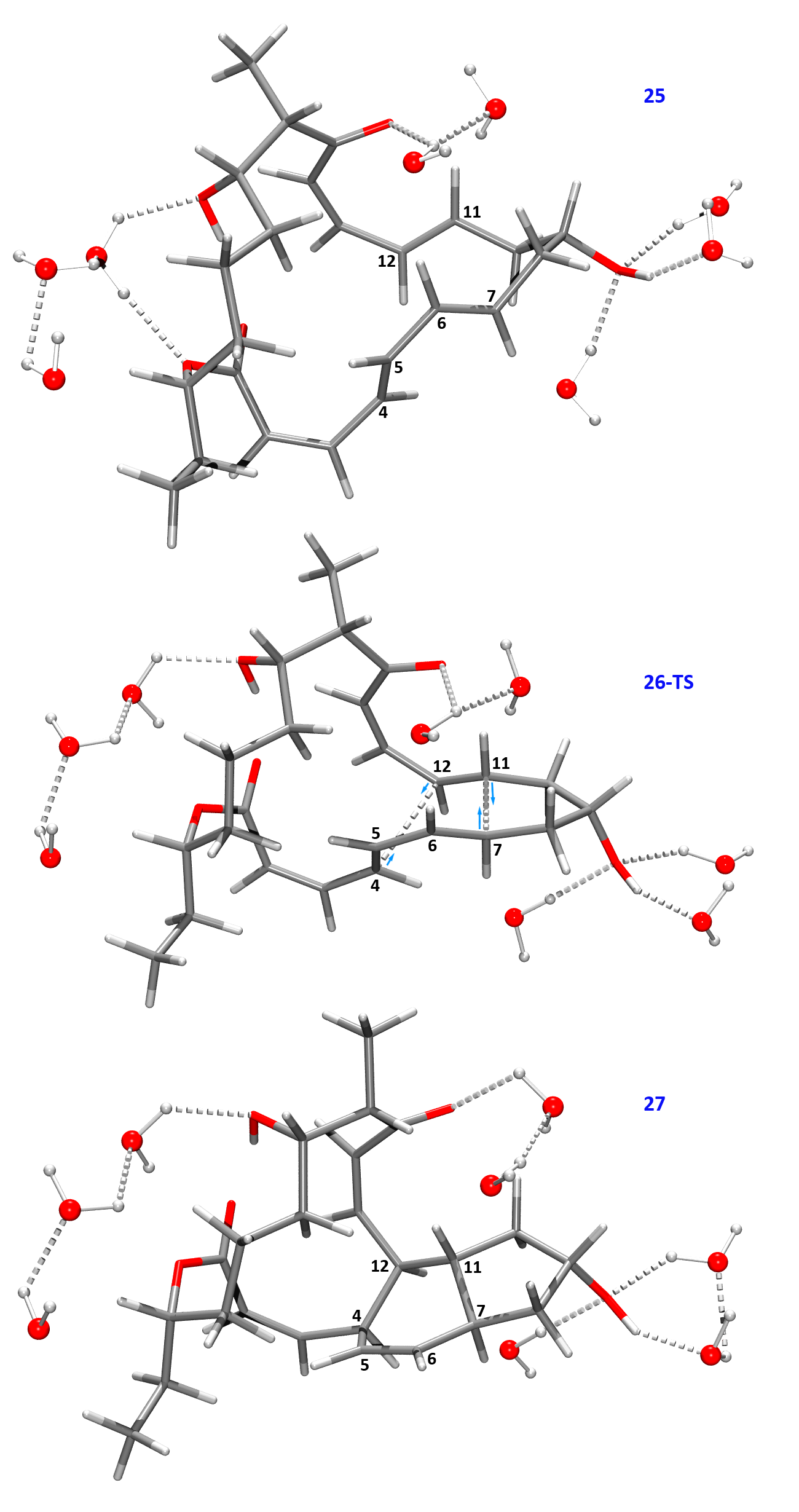

Supplement: S8 Fig — PM6 optimized molecular structures of the reagent, transition state and product of cycloaddition step coordinated by eight water molecules (see S5 Table for interatomic distances). Displacement vectors corresponding to an imaginary frequency are shown for the transition state (see Fig. 2 for pericycle atomic numbers). (TIF) [file pone.0119984.s008.tif]

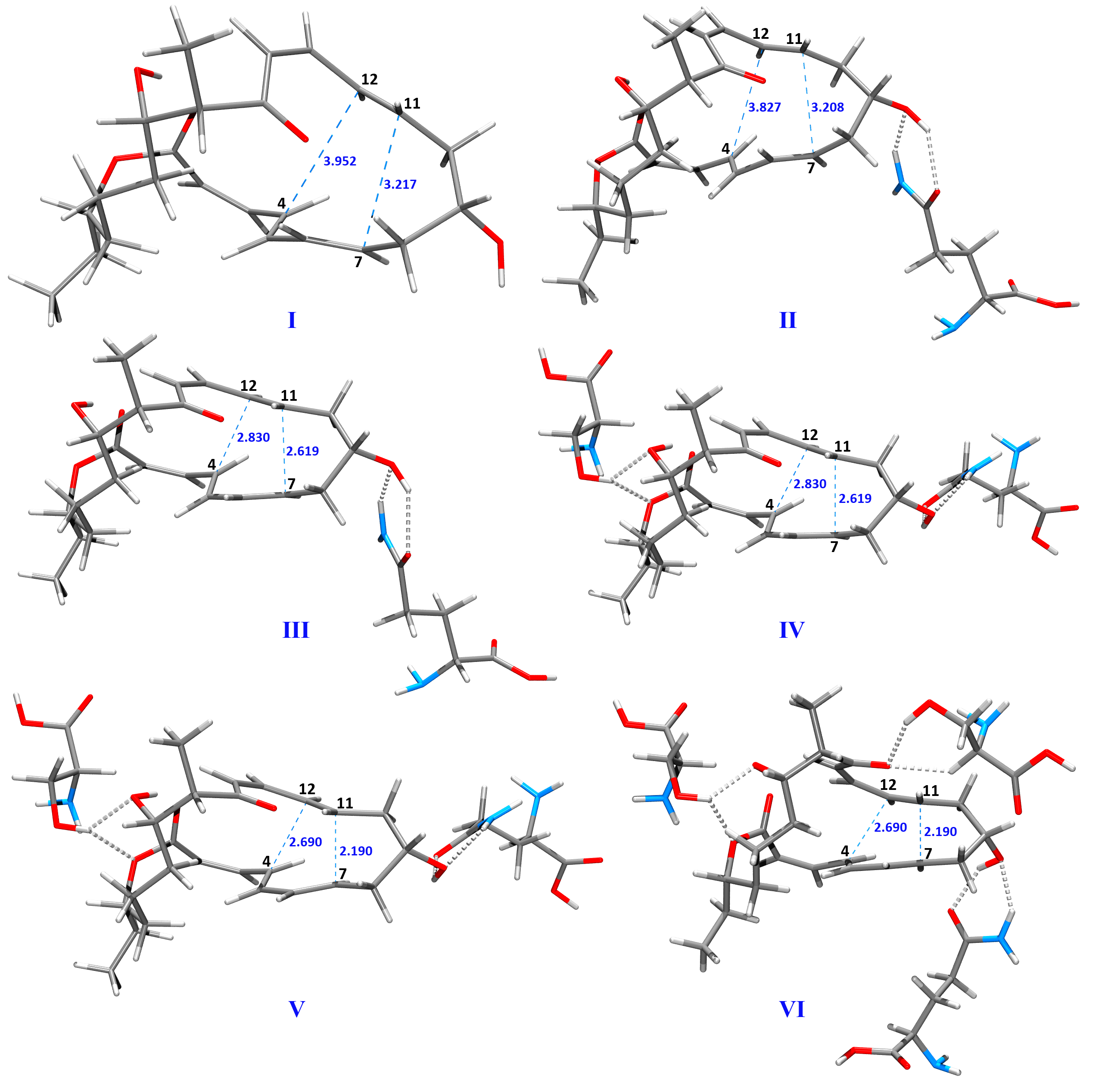

Supplement: S9 Fig — PM6 optimized molecular structures (I-VI) of enzyme-catalyzed cycloaddition reactions of Spinoson A (see Fig. 3 for energy surface). (TIF) [file pone.0119984.s009.tif]

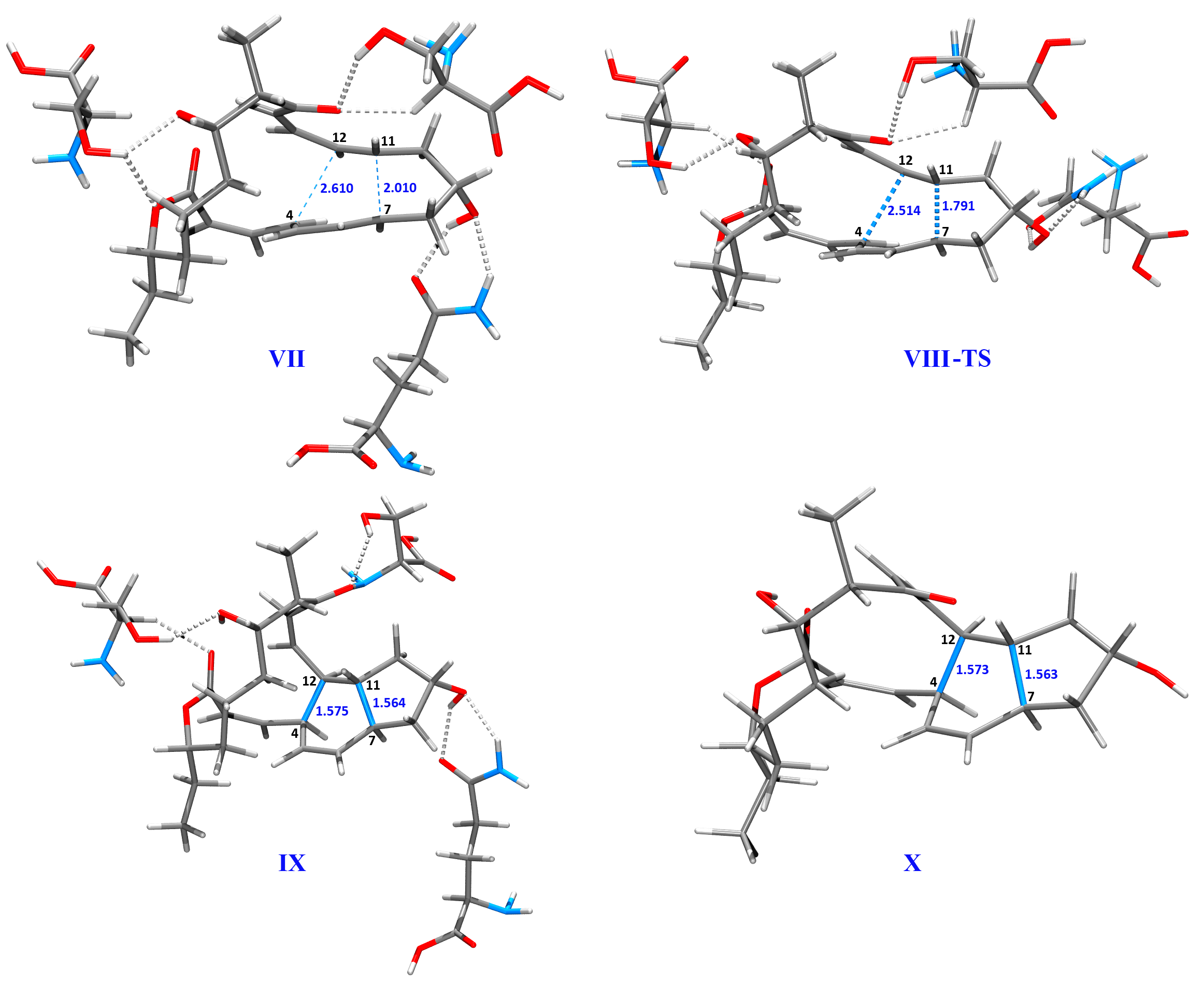

Supplement: S10 Fig — PM6 optimized molecular structures (VII-X) of enzyme-catalyzed cycloaddition reactions of Spinoson A (see Fig. 3 for energy surface). (TIF) [file pone.0119984.s010.tif]
