# Supplementary material for: Computational Study of a Model System of Enzyme-Mediated [4+2] Cycloaddition Reaction
Source: PLoS One. 2015 Apr 8;10(4):e0119984. doi: 10.1371/journal.pone.0119984 (PMC4390235; doi:10.1371/journal.pone.0119984)
Supplement: S1 File — (DOC) [file pone.0119984.s011.doc]

*Supporting Information*

*for the article*

**Computational Study of a Model System of Enzyme-Mediated [4+2] Cycloaddition Reaction**

Evgeniy G. Gordeev, and Valentine P. Ananikov*

*Zelinsky Institute of Organic Chemistry, Russian Academy of Sciences, Leninsky Pr. 47, Moscow, 119991, Russia; E-mail: val@ioc.ac.ru; http://AnanikovLab.ru*

**XYZ structures for studied molecules**

**Structure 1** (Figure S1, Figure 2 (reaction a))

| 1,3-butadien | |
| --- | --- |
| B3LYP/6-311+G(d) structure | PM6 structure |
| E(B3LYP/6-311+G(d)) = -156.03027942 a.u. | E(PM6) = 0.04554976 a.u. |
| C 0.60070 1.74980 0.00000  C 0.60070 0.41140 0.00000  H -0.32430 2.31980 0.00000  H 1.52280 2.32040 0.00000  H 1.55130 -0.12030 0.00000  C -0.60070 -0.41140 0.00000  C -0.60070 -1.74980 0.00000  H -1.55130 0.12030 0.00000  H -1.52280 -2.32040 0.00000  H 0.32430 -2.31980 0.00000 | C -1.11890 1.46190 0.00000  C 0.00000 0.73430 0.00000  H -2.11350 1.03960 0.00000  H -1.12100 2.54250 0.00000  H 0.98410 1.21110 0.00000  C 0.00000 -0.73430 0.00000  C 1.11890 -1.46190 0.00000  H -0.98410 -1.21110 0.00000  H 1.12100 -2.54250 0.00000  H 2.11350 -1.03960 0.00000 |
| Ethylene | |
| B3LYP/6-311+G(d) structure | PM6 structure |
| E(B3LYP/6-311+G(d)) = -78.60836203 a.u. | E(PM6) = 0.02511160 a.u. |
| C 0.00000 -0.66420 0.00000  C 0.00000 0.66420 0.00000  H -0.92260 -1.23790 0.00000  H 0.92260 -1.23790 0.00000  H 0.92260 1.23790 0.00000  H -0.92260 1.23790 0.00000 | C 0.00000 -0.66350 0.00000  C 0.00000 0.66350 0.00000  H 0.90190 -1.26100 0.00000  H -0.90190 -1.26100 0.00000  H -0.90190 1.26100 0.00000  H 0.90190 1.26100 0.00000 |

**Structure 2-TS** (Figure S1, Figure 2 (reaction a))

| B3LYP/6-311+G(d) structure | PM6 structure |
| --- | --- |
| E(B3LYP/6-311+G(d)) = -234.59958740 a.u. | E(PM6) = 0.11286019 a.u. |
| C 1.56550 0.69340 -0.22730  C 1.56540 -0.69320 -0.22780  H 1.47110 1.23580 -1.15910  H 2.06900 -1.23320 0.56640  H 2.06900 1.23250 0.56750  H 1.47080 -1.23480 -1.16000  C -1.31860 -0.70210 -0.28790  C -0.44050 -1.43510 0.48890  C -0.44080 1.43520 0.48860  C -1.31870 0.70170 -0.28810  H -1.86570 -1.21180 -1.07760  H -0.38420 -2.51270 0.37190  H -0.12640 1.07540 1.45950  H -1.86600 1.21120 -1.07780  H -0.12610 -1.07480 1.45970  H -0.38530 2.51290 0.37130 | C 1.45650 0.69080 -0.25400  C 1.45640 -0.69100 -0.25400  H 1.29280 1.24310 -1.17150  H 1.98390 -1.24710 0.51080  H 1.98410 1.24690 0.51080  H 1.29270 -1.24320 -1.17150  C -1.26010 -0.70550 -0.28520  C -0.37980 -1.41020 0.50980  C -0.37960 1.41030 0.50980  C -1.26010 0.70560 -0.28520  H -1.84610 -1.22270 -1.04450  H -0.26620 -2.48070 0.40100  H -0.06440 1.04020 1.48050  H -1.84590 1.22300 -1.04450  H -0.06450 -1.04020 1.48050  H -0.26590 2.48070 0.40100 |

**Structure 3** (Figure S1, Figure 2 (reaction a))

| B3LYP/6-311+G(d) structure | PM6 structure |
| --- | --- |
| E(B3LYP/6-311+G(d)) = -234.69085105 a.u. | E(PM6) = -0.00618507 a.u. |
| C -0.66570 1.30270 -0.17990  C -1.40070 0.09510 0.34160  C 1.40090 0.09140 0.34170  C 0.66910 1.30090 -0.17990  H -1.22830 2.15940 -0.54150  H -2.46310 0.13380 0.08510  H 1.35770 0.09040 1.44050  H 1.23400 2.15620 -0.54140  H -1.35760 0.09410 1.44040  H 2.46350 0.12740 0.08540  C 0.77510 -1.22040 -0.19000  C -0.77830 -1.21850 -0.18980  H 1.13320 -1.37570 -1.21180  H -1.16310 -2.05850 0.39670  H 1.15790 -2.06160 0.39620  H -1.13700 -1.37320 -1.21150 | C 0.66660 1.30730 0.04380  C 1.49040 0.05480 0.11460  C -1.49060 0.05380 -0.11420  C -0.66750 1.30680 -0.04400  H 1.23620 2.23400 0.08520  H 1.85800 -0.06920 1.15510  H -1.85870 -0.07050 -1.15450  H -1.23770 2.23310 -0.08560  H 2.39460 0.15900 -0.51720  H -2.39440 0.15740 0.51800  C -0.70060 -1.19410 0.31180  C 0.70150 -1.19360 -0.31210  H -0.61610 -1.22000 1.41540  H 0.61710 -1.21910 -1.41560  H -1.24870 -2.10800 0.02060  H 1.25030 -2.10710 -0.02110 |

**Structure 4** (Figure S2, Figure 2 (reaction b))

| B3LYP/6-311+G(d) structure | PM6 structure |
| --- | --- |
| E(B3LYP/6-311+G(d)) = -351.40160295 a.u. | E(PM6) = 0.04480062 a.u. |
| C 3.01380 1.41990 -0.33920  C 3.12030 0.12570 -0.01980  C 2.02140 -0.84240 0.08200  C 0.75870 -0.57000 0.43770  C -0.34090 -1.59220 0.50360  C -1.47370 -1.41320 -0.53330  C -2.49620 -0.29740 -0.24080  C -1.99070 1.11080 -0.38770  C -2.11260 2.07580 0.52180  H 2.06010 1.87520 -0.58850  H 3.88440 2.06630 -0.37060  H 4.11550 -0.27620 0.16800  H 2.28760 -1.87860 -0.13030  H 0.49470 0.44980 0.70670  H 0.09510 -2.58800 0.36760  H -0.78730 -1.58730 1.50780  H -2.02660 -2.35800 -0.58840  H -1.03450 -1.26230 -1.52650  H -3.33230 -0.43050 -0.94150  H -2.91750 -0.43560 0.76210  H -1.50790 1.34310 -1.33770  H -1.74730 3.08170 0.34040  H -2.58470 1.89840 1.48490 | C -2.86430 1.44770 0.33230  C -3.05770 0.18330 -0.04890  C -2.01630 -0.85020 -0.07230  C -0.73500 -0.61310 -0.38170  C 0.31620 -1.68050 -0.42490  C 1.46560 -1.40430 0.55810  C 2.41860 -0.29170 0.09710  C 1.89380 1.08710 0.37140  C 2.07290 2.12130 -0.44840  H -1.91110 1.83380 0.66280  H -3.65050 2.18730 0.34640  H -4.04540 -0.16560 -0.36470  H -2.36840 -1.85400 0.17390  H -0.38050 0.38900 -0.63540  H -0.11710 -2.67710 -0.20140  H 0.71090 -1.75740 -1.46040  H 2.04600 -2.33850 0.69510  H 1.04960 -1.16240 1.55630  H 3.39250 -0.40260 0.62260  H 2.65330 -0.42000 -0.97930  H 1.35570 1.19400 1.31590  H 1.70920 3.11510 -0.23460  H 2.59300 2.06040 -1.39230 |

**Structure 5-TS** (Figure S2, Figure 2 (reaction b))

| B3LYP/6-311+G(d) structure | PM6 structure |
| --- | --- |
| E(B3LYP/6-311+G(d)) = -351.36007700 a.u. | E(PM6) = 0.09469108 a.u. |
| C 2.42260 0.29230 0.55040  C 2.21020 -0.83190 -0.23450  C 0.95590 -1.45510 -0.32060  C -0.13400 -0.92870 0.35510  C -1.57300 -1.34760 0.22240  C -2.44890 -0.09510 -0.08950  C -1.63940 1.18990 0.15090  C -0.28820 1.07150 -0.52000  C 0.86430 1.77010 -0.17380  H 1.92660 0.40000 1.50650  H 3.36660 0.82370 0.48000  H 2.96470 -1.10240 -0.96980  H 0.79320 -2.19990 -1.09760  H 0.07880 -0.41770 1.28810  H -1.68970 -2.10490 -0.55860  H -1.90660 -1.81070 1.15830  H -3.37580 -0.09490 0.49160  H -2.74900 -0.12160 -1.14260  H -2.19090 2.04940 -0.25160  H -1.51280 1.38500 1.22280  H -0.34670 0.73460 -1.55330  H 1.54880 2.09400 -0.94630  H 0.87660 2.37330 0.73010 | C 2.30980 0.30590 0.61560  C 2.16810 -0.81750 -0.18260  C 0.91690 -1.45390 -0.31030  C -0.16930 -0.93080 0.36910  C -1.60080 -1.30190 0.11510  C -2.44190 -0.00720 0.04400  C -1.54410 1.24540 0.10610  C -0.22370 0.98190 -0.57220  C 0.95550 1.63830 -0.25110  H 1.75250 0.41490 1.54180  H 3.22480 0.88280 0.59770  H 2.97450 -1.11930 -0.84860  H 0.79120 -2.22990 -1.06080  H -0.00640 -0.43820 1.33750  H -1.71070 -1.87800 -0.82300  H -1.97540 -1.96400 0.91950  H -3.17770 0.01960 0.86670  H -3.02900 -0.00170 -0.89230  H -2.04860 2.09810 -0.39100  H -1.39730 1.55700 1.15810  H -0.31620 0.54140 -1.57000  H 1.69430 1.82850 -1.02060  H 1.00210 2.32580 0.58270 |

**Structure 6** (Figure S2, Figure 2 (reaction b))

| B3LYP/6-311+G(d) structure | PM6 structure |
| --- | --- |
| E(B3LYP/6-311+G(d)) = -351.44886954 a.u. | E(PM6) = -0.01198243 a.u. |
| C -2.31630 -0.62460 -0.18870  C -2.14770 0.87710 -0.11290  C -0.98900 1.49520 0.13750  C 0.26940 0.71240 0.38860  C 1.61690 1.23340 -0.13650  C 2.53300 -0.02940 -0.11860  C 1.58330 -1.25720 0.04810  C 0.18910 -0.68730 -0.23610  C -1.06140 -1.41690 0.24180  H -3.17940 -0.92530 0.41790  H -2.58770 -0.89250 -1.21990  H -3.04340 1.47080 -0.28690  H -0.95250 2.58200 0.18840  H 0.37880 0.58070 1.47860  H 1.49770 1.61060 -1.15810  H 2.02050 2.05420 0.46270  H 3.25660 0.01800 0.69930  H 3.11510 -0.10130 -1.04080  H 1.84860 -2.08600 -0.61420  H 1.62830 -1.64230 1.07350  H 0.09860 -0.54300 -1.32420  H -1.11160 -2.43820 -0.15160  H -1.03320 -1.50450 1.33540 | C -2.32500 -0.60810 -0.06910  C -2.13660 0.88460 -0.14930  C -0.96520 1.51100 0.02190  C 0.25010 0.71790 0.38820  C 1.64160 1.23140 -0.01930  C 2.51960 -0.04410 -0.08120  C 1.57040 -1.26880 -0.04540  C 0.17290 -0.67760 -0.29520  C -1.04880 -1.42230 0.24270  H -3.09570 -0.83380 0.69560  H -2.74790 -0.95310 -1.03620  H -3.04720 1.43540 -0.38120  H -0.85100 2.58610 -0.06430  H 0.23810 0.58740 1.50160  H 1.61000 1.73460 -0.99970  H 2.03660 1.96520 0.69830  H 3.22400 -0.07580 0.76510  H 3.13000 -0.04890 -0.99810  H 1.84180 -2.01650 -0.80490  H 1.62440 -1.77870 0.93010  H 0.04270 -0.53780 -1.39630  H -1.12300 -2.42900 -0.20430  H -0.95720 -1.57910 1.33330 |

**Structure 7** (Figure S2, Figure 2 (reaction c))

| B3LYP/6-311+G(d) structure | PM6 structure |
| --- | --- |
| E(B3LYP/6-311+G(d)) = -426.63902400 a.u. | E(PM6) = -0.02684140 a.u. |
| C 3.50700 0.79190 -0.28120  C 3.26680 -0.51600 -0.13830  C 1.95110 -1.15130 0.00260  C 0.85470 -0.59080 0.53120  C -0.47810 -1.27540 0.63340  C -1.56510 -0.76630 -0.34020  C -2.09920 0.64230 -0.05440  C -1.18400 1.77570 -0.42160  C -0.89460 2.81430 0.35970  H 2.70610 1.52320 -0.33310  H 4.51940 1.17270 -0.35970  H 4.11620 -1.19810 -0.14280  H 1.89160 -2.18630 -0.33590  H 0.91510 0.42120 0.92140  H -0.33860 -2.34720 0.44070  H -0.87790 -1.18960 1.65140  H -1.15870 -0.79580 -1.36210  H -3.02010 0.73290 -0.64370  H -2.39880 0.71040 0.99690  H -0.76660 1.74550 -1.42820  H -0.26000 3.62550 0.01760  H -1.28620 2.89810 1.37030  O -2.71790 -1.61610 -0.26480  H -2.45070 -2.52980 -0.42180 | C -3.88820 0.06230 -0.26950  C -2.81460 0.85310 -0.31750  C -1.54180 0.56930 0.35550  C -1.00270 -0.65310 0.45060  C 0.29860 -0.92700 1.13870  C 1.47360 -1.12840 0.16280  C 1.73540 0.09320 -0.73580  C 2.33110 1.23170 0.03340  C 1.85550 2.47500 -0.00040  H -3.92220 -0.87000 0.27580  H -4.81820 0.29770 -0.76600  H -2.83170 1.79780 -0.86940  H -1.04110 1.44650 0.77500  H -1.49220 -1.53020 0.02490  H 0.55880 -0.10790 1.84170  H 0.19650 -1.83950 1.76510  H 2.39440 -1.42250 0.72180  H 2.43130 -0.20610 -1.55090  H 0.80030 0.39610 -1.24940  H 3.21000 0.97210 0.62780  H 2.30150 3.29380 0.54510  H 0.99180 2.77450 -0.57550  O 1.26070 -2.28340 -0.65900  H 0.45340 -2.19180 -1.20060 |

**Structure 8-TS** (Figure S2, Figure 2 (reaction c))

| B3LYP/6-311+G(d) structure | PM6 structure |
| --- | --- |
| E(B3LYP/6-311+G(d)) = -426.59736363 a.u. | E(PM6) = 0.02175324 a.u. |
| C -2.78990 0.26210 -0.64440  C -2.60550 -0.85710 0.15520  C -1.35040 -1.46500 0.30850  C -0.23400 -0.92330 -0.30930  C 1.20170 -1.31470 -0.09960  C 2.03190 -0.05460 0.29840  C 1.23240 1.21310 -0.00490  C -0.15770 1.07980 0.57360  C -1.29290 1.76210 0.14730  H -2.24710 0.37120 -1.57450  H -3.74300 0.78130 -0.62600  H -3.39490 -1.13640 0.84920  H -1.22020 -2.21010 1.09120  H -0.40100 -0.41390 -1.25160  H 1.29570 -2.09720 0.66080  H 1.62370 -1.71040 -1.03040  H 2.21500 -0.08740 1.38210  H 1.75830 2.07220 0.42710  H 1.19800 1.38070 -1.08710  H -0.16880 0.75360 1.61190  H -2.02580 2.08740 0.87310  H -1.25410 2.35590 -0.76180  O 3.28490 0.03790 -0.38000  H 3.87120 -0.66350 -0.07300 | C 2.56730 0.31890 0.81420  C 2.54200 -0.80610 0.00590  C 1.32450 -1.45400 -0.28530  C 0.15480 -0.94160 0.24660  C -1.22490 -1.31150 -0.19990  C -2.06660 -0.01980 -0.32820  C -1.18760 1.24590 -0.20810  C 0.20910 0.97900 -0.69910  C 1.33350 1.63940 -0.22630  H 1.89280 0.42350 1.65960  H 3.47280 0.90270 0.91710  H 3.43260 -1.10150 -0.54670  H 1.30550 -2.23130 -1.04520  H 0.18690 -0.44950 1.22800  H -1.21490 -1.85470 -1.16370  H -1.70840 -2.00590 0.51720  H -2.66590 -0.01950 -1.26560  H -1.63810 2.07100 -0.79690  H -1.19110 1.61030 0.83750  H 0.25270 0.53480 -1.69880  H 2.16660 1.83060 -0.89340  H 1.26710 2.33190 0.60250  O -3.09400 0.00740 0.66990  H -2.71880 -0.00360 1.57050 |

**Structure 9** (Figure S2, Figure 2 (reaction c))

| B3LYP/6-311+G(d) structure | PM6 structure |
| --- | --- |
| E(B3LYP/6-311+G(d)) = -426.68630525 a.u. | E(PM6) = -0.08137802 a.u. |
| C 2.69880 -0.61290 0.01000  C 2.51540 0.88850 -0.03710  C 1.33340 1.50280 -0.15070  C 0.05940 0.71360 -0.26220  C -1.22220 1.20620 0.42150  C -2.14140 -0.04940 0.41280  C -1.19540 -1.26680 0.23390  C 0.21980 -0.68600 0.34840  C 1.40680 -1.41080 -0.27570  H 3.48660 -0.90530 -0.69500  H 3.09010 -0.88420 1.00090  H 3.42260 1.48560 0.03720  H 1.28720 2.58950 -0.19250  H -0.18100 0.58810 -1.33000  H -1.00780 1.50960 1.45330  H -1.69620 2.05600 -0.07950  H -2.71290 -0.11860 1.34620  H -1.39710 -2.06220 0.95580  H -1.37040 -1.67910 -0.76490  H 0.44430 -0.54120 1.41660  H 1.50660 -2.43250 0.10680  H 1.25030 -1.49560 -1.35820  O -3.05710 -0.05750 -0.69210  H -3.71270 0.63910 -0.56450 | C 2.68170 -0.62950 -0.09280  C 2.52180 0.86530 0.00460  C 1.34700 1.50670 -0.04420  C 0.09190 0.72690 -0.27990  C -1.24640 1.26410 0.25380  C -2.11880 -0.00630 0.43050  C -1.19720 -1.24260 0.30920  C 0.22310 -0.66630 0.40350  C 1.36950 -1.42860 -0.25950  H 3.35960 -0.86490 -0.93860  H 3.20660 -0.97970 0.82120  H 3.45880 1.40480 0.13790  H 1.25550 2.58320 0.04920  H -0.01170 0.58570 -1.38870  H -1.11730 1.79180 1.21220  H -1.70540 1.98000 -0.44430  H -2.69460 0.01280 1.37610  H -1.40470 -1.99180 1.08550  H -1.38120 -1.74150 -0.66290  H 0.47530 -0.52380 1.48310  H 1.47900 -2.43500 0.18150  H 1.15690 -1.58940 -1.33310  O -3.06140 -0.15120 -0.64920  H -3.62060 0.63950 -0.73650 |

**Structure 10** (Figure S3, Figure 2 (reaction d))

| B3LYP/6-311+G(d) structure | PM6 structure |
| --- | --- |
| E(B3LYP/6-311+G(d)) = -581.47957105 a.u. | E(PM6) = 0.01765826 a.u. |
| C -2.59760 2.16410 0.00070  C -2.69470 3.46520 0.30640  H -1.95060 4.18610 -0.02200  H -3.51740 3.85230 0.89740  C 2.96480 0.15320 0.06090  C 2.80660 -1.11010 -0.38720  C 1.56900 -1.88370 -0.40300  C 0.47780 -1.66510 0.34770  C -0.77250 -2.49120 0.29200  C -2.09990 -1.70560 0.27990  C -2.34280 -0.80570 -0.94500  C -1.36940 0.32200 -1.17390  C -1.50810 1.60000 -0.78160  H 2.10260 0.70030 0.43890  H 3.67930 -1.61210 -0.80420  H 1.55630 -2.72700 -1.09400  H 0.47850 -0.86130 1.07950  H -0.75020 -3.16660 -0.57110  H -0.80680 -3.14510 1.17860  H -2.90850 -2.45210 0.28130  H -2.34530 -1.45560 -1.82810  H -3.36060 -0.41820 -0.84960  H -0.47820 0.07080 -1.74120  H -0.72800 2.30260 -1.07290  O -2.23290 -0.87660 1.43890  H -2.21370 -1.42910 2.22990  C 4.23080 0.85760 0.06120  C 4.40510 2.11330 0.49960  H 5.09010 0.31030 -0.32500  H 5.37660 2.59420 0.47860  H 3.58000 2.69880 0.89530  H -3.35560 1.48040 0.37130 | C 0.19500 2.12570 0.64620  C 1.42480 2.63370 0.75640  H 1.89370 3.24630 0.00010  H 2.05320 2.47400 1.62110  C 2.55630 -0.87780 -0.03380  C 1.94990 -2.07600 0.01800  C 0.52530 -2.26510 0.29550  C -0.42790 -1.38330 -0.03910  C -1.87560 -1.60050 0.26820  C -2.78060 -0.51760 -0.34390  C -2.56910 0.88390 0.25130  C -1.86860 1.79330 -0.70760  C -0.65860 2.34080 -0.52550  H 1.99810 0.04890 0.13400  H 2.51000 -3.00230 -0.13530  H 0.28100 -3.20280 0.80010  H -0.18630 -0.45010 -0.55490  H -2.19860 -2.60040 -0.08890  H -2.03060 -1.63030 1.36960  H -2.70270 -0.51630 -1.45400  H -3.57140 1.30810 0.50540  H -2.03900 0.81910 1.22380  H -2.43700 2.00600 -1.61750  H -0.24350 3.00590 -1.28960  O -4.15540 -0.81120 -0.00240  H -4.42230 -1.68770 -0.33020  C 3.98730 -0.72260 -0.30550  C 4.59200 0.46760 -0.34700  H 4.54770 -1.64590 -0.47630  H 5.64630 0.58780 -0.54750  H 4.08030 1.40590 -0.18510  H -0.23660 1.51130 1.44420 |

**Structure 11-TS** (Figure S3, Figure 2 (reaction d))

| B3LYP/6-311+G(d) structure | PM6 structure |
| --- | --- |
| E(B3LYP/6-311+G(d)) = -581.44197916 a.u. | E(PM6) = 0.06536324 a.u. |
| C 1.01830 1.94470 -0.14070  C 2.27090 2.45770 -0.21610  H 2.97220 2.13960 -0.97920  H 2.60010 3.24650 0.45090  C 1.79920 -1.15620 0.38370  C 1.08780 -2.15780 -0.26440  C -0.30370 -2.17770 -0.35990  C -1.10190 -1.10880 0.10080  C -2.60820 -1.11050 -0.01890  C -3.06760 0.34870 0.11830  C -2.01280 1.24230 -0.57060  C -0.81030 0.42560 -1.05160  C 0.51810 0.91890 -0.99660  H 1.28170 -0.51890 1.09090  H 1.64970 -2.90120 -0.82790  H -0.76120 -2.92450 -1.00620  H -0.76920 -0.62300 1.01470  H -2.91130 -1.51100 -0.99410  H -3.08420 -1.73920 0.74330  H -4.06300 0.49050 -0.31970  H -2.45010 1.76490 -1.42690  H -1.71960 2.01150 0.14400  H -1.02020 -0.08840 -1.98750  H 1.21140 0.54660 -1.74380  O -3.10430 0.75870 1.49600  H -3.79260 0.26350 1.95730  C 3.20620 -0.94110 0.26890  C 3.87930 0.01330 0.95180  H 3.74800 -1.55180 -0.45310  H 4.94140 0.17470 0.80370  H 3.39330 0.62340 1.70380  H 0.35720 2.33170 0.63280 | C -1.08530 -1.78320 -0.02060  C -2.25860 -2.41120 -0.16500  H -2.98800 -2.15070 -0.91810  H -2.56760 -3.23530 0.45910  C -1.74720 1.01720 0.35730  C -1.08220 2.09590 -0.20690  C 0.31600 2.17990 -0.19920  C 1.07500 1.10810 0.29120  C 2.58080 1.10120 0.21100  C 3.04390 -0.33270 -0.12130  C 1.83310 -1.18740 -0.55110  C 0.69020 -0.29450 -0.98900  C -0.65600 -0.67630 -0.86530  H -1.29090 0.45190 1.17480  H -1.64190 2.84690 -0.76720  H 0.80100 3.00300 -0.71890  H 0.68210 0.55090 1.15740  H 2.95410 1.80840 -0.55320  H 3.03360 1.43960 1.16410  H 3.85820 -0.34160 -0.87850  H 2.11220 -1.86760 -1.37930  H 1.53540 -1.85940 0.27860  H 0.93740 0.29010 -1.88980  H -1.36780 -0.31810 -1.61090  O 3.69440 -0.92910 1.00640  H 3.10230 -0.97150 1.78090  C -3.17550 0.81030 0.13500  C -3.90690 -0.06500 0.83420  H -3.63050 1.41040 -0.65810  H -4.96010 -0.22580 0.65920  H -3.50880 -0.67920 1.62930  H -0.38080 -2.09320 0.75790 |

**Structure 12** (Figure S3, Figure 2 (reaction d))

| B3LYP/6-311+G(d) structure | PM6 structure |
| --- | --- |
| E(B3LYP/6-311+G(d)) = -581.50193989 a.u. | E(PM6) = -0.02490631 a.u. |
| C 0.62150 1.86960 0.84030  C 0.69490 3.18050 0.61890  H 0.93540 3.58350 -0.36170  H 0.51630 3.90320 1.40880  C 1.99080 -0.18510 0.28360  C 1.45540 -1.33070 1.12990  C 0.16330 -1.62600 1.30570  C -0.91820 -0.79820 0.67970  C -2.16480 -1.46990 0.08680  C -2.79490 -0.34890 -0.78870  C -1.64740 0.65210 -1.08890  C -0.38650 -0.02360 -0.53270  C 0.86670 0.80460 -0.19660  H 2.69270 0.38580 0.90430  H 2.21590 -1.94600 1.60610  H -0.11570 -2.46040 1.94700  H -1.28120 -0.07480 1.42560  H -1.87260 -2.31970 -0.54170  H -2.86940 -1.84170 0.83690  H -3.21550 -0.76770 -1.71080  H -1.57170 0.89210 -2.15250  H -1.86560 1.58170 -0.55700  H -0.06960 -0.78160 -1.26360  H 1.22380 1.29900 -1.10700  O -3.82240 0.37660 -0.09870  H -4.59100 -0.19540 0.01620  C 2.78300 -0.73460 -0.88180  C 4.09220 -0.56970 -1.06010  H 2.21830 -1.31510 -1.61040  H 4.60860 -0.99080 -1.91690  H 4.69950 -0.00690 -0.35580  H 0.38410 1.52170 1.84480 | C -0.61450 1.73970 -0.85900  C -1.06270 2.99060 -0.78300  H -1.63600 3.37790 0.04640  H -0.88620 3.72860 -1.55200  C -1.92320 -0.33230 -0.30050  C -1.36630 -1.63460 -0.83560  C -0.06410 -1.91500 -0.96260  C 0.95380 -0.88140 -0.60060  C 2.35110 -1.32890 -0.14070  C 2.85110 -0.15800 0.74580  C 1.64850 0.76850 1.03850  C 0.42360 -0.04750 0.60090  C -0.85890 0.70120 0.20630  H -2.48900 0.15010 -1.14230  H -2.13430 -2.35560 -1.11670  H 0.30120 -2.86150 -1.34810  H 1.08380 -0.20780 -1.49100  H 2.30770 -2.26870 0.43240  H 3.02240 -1.50740 -0.99430  H 3.35430 -0.51780 1.66420  H 1.60360 1.07090 2.09370  H 1.75450 1.70460 0.45230  H 0.15390 -0.74630 1.43250  H -1.26390 1.20470 1.12060  O 3.78390 0.67520 0.03220  H 4.55590 0.16370 -0.26520  C -2.87040 -0.65400 0.83350  C -4.15030 -0.29050 0.84900  H -2.41920 -1.21470 1.65370  H -4.82050 -0.52100 1.66470  H -4.63020 0.26410 0.05560  H -0.03490 1.38680 -1.71610 |

**Structure 13** (Figure S4, Figure 2 (reaction e))

| B3LYP/6-311+G(d) structure | PM6 structure |
| --- | --- |
| E(B3LYP/6-311+G(d)) = -1311.40135124 a.u. | E(PM6) = -0.31531296 a.u.  E^SP^(B3LYP/6-311+G(d,p)) = -1311.3842628 a.u.  E^SP^(O3LYP/6-311+G(d,p)) = -1310.8592787 a.u.  E^SP^(M062X/6-311+G(d,p)) = -1310.8357044 a.u. |
| C 2.21100 1.97730 -1.27540  C 1.46440 2.30050 -0.20060  H 1.90640 2.35890 0.78690  C 1.20790 -2.51710 -0.01680  C 2.18530 -2.96550 0.80250  C 3.62270 -2.80890 0.61090  C 4.21700 -1.93720 -0.22180  C 5.70040 -1.74910 -0.33780  C 6.24050 -0.46180 0.33010  C 5.82610 0.86190 -0.34790  C 4.37240 1.21670 -0.26980  C 3.62230 1.61720 -1.31020  H 1.47140 -2.04020 -0.95880  H 1.88520 -3.47770 1.71540  H 4.25460 -3.43200 1.24270  H 3.59730 -1.28370 -0.82800  H 6.22330 -2.58810 0.13010  H 5.99410 -1.73860 -1.39790  H 5.90430 -0.44760 1.37080  H 6.40440 1.64480 0.16050  H 6.15810 0.86070 -1.39420  H 4.08460 1.64210 -2.29590  O 7.66460 -0.52320 0.42870  H 8.04640 -0.57140 -0.45770  C -0.19450 -2.58090 0.30470  C -1.16430 -2.02340 -0.44650  H -0.48510 -3.06730 1.23390  H -0.93720 -1.51250 -1.37640  H 1.69810 1.98840 -2.23500  O -3.31400 -1.27400 -0.85800  C 0.02010 2.60210 -0.34670  C -0.77150 3.04420 0.89210  C -2.30810 2.90540 0.70590  C -0.29050 2.41580 2.21270  C -2.79420 1.45280 0.58030  C -4.32480 1.34260 0.55430  C -4.89480 -0.08350 0.65260  C -4.72240 -0.97570 -0.58200  C -2.57190 -2.00730 -0.00020  C -5.56970 -2.25530 -0.57180  C -7.05100 -1.99360 -0.86340  O -2.99610 -2.54470 1.00230  O -0.52200 2.54570 -1.44610  H -0.99590 2.64960 3.01460  H 0.68080 2.80410 2.52590  H -0.21210 1.32780 2.15240  H -2.76710 3.34220 1.60030  H -5.16790 -2.92480 -1.33990  H -5.45140 -2.77080 0.38310  H -7.52600 -1.38250 -0.09120  H -7.60370 -2.93550 -0.91220  H -7.18920 -1.48230 -1.82180  H -5.97430 0.00240 0.81960  H -4.49640 -0.60160 1.52910  H -4.72700 1.91590 1.39940  H -4.70880 1.83780 -0.34330  H -2.41050 0.87650 1.43110  H -2.36770 1.00500 -0.31970  O -2.77330 3.72410 -0.36320  H -2.29780 3.43740 -1.15800  H -0.58840 4.12810 0.95140  H -4.98650 -0.39580 -1.47190  H 3.91570 1.14430 0.71650 | C 2.02500 1.59350 -1.31990  C 1.31510 2.14260 -0.32090  H 1.78210 2.56510 0.56640  C 1.38380 -2.04680 0.21190  C 2.34490 -2.77630 0.80690  C 3.77840 -2.51530 0.68350  C 4.35200 -1.90330 -0.36300  C 5.81870 -1.61280 -0.43460  C 6.16870 -0.33450 0.35270  C 5.70680 0.96190 -0.33970  C 4.22240 1.12580 -0.28570  C 3.48170 1.46770 -1.34900  H 1.62690 -1.20210 -0.43930  H 2.09060 -3.62020 1.45780  H 4.37780 -2.86500 1.52970  H 3.77190 -1.56860 -1.22510  H 6.41300 -2.45490 -0.01540  H 6.14600 -1.52540 -1.48880  H 5.79300 -0.39310 1.40200  H 6.18930 1.82620 0.17400  H 6.08780 1.00010 -1.37930  H 3.93270 1.65060 -2.32850  O 7.58590 -0.27500 0.55400  H 8.07270 -0.24110 -0.29080  C -0.03790 -2.31340 0.41910  C -1.00630 -1.63480 -0.21520  H -0.29360 -3.09610 1.14740  H -0.81790 -0.85500 -0.96370  H 1.50930 1.20230 -2.21180  O -3.19250 -1.12660 -0.76080  C -0.16450 2.20710 -0.40340  C -0.89970 3.04290 0.63910  C -2.44510 2.86240 0.55480  C -0.39880 2.77840 2.05540  C -2.89440 1.41330 0.77010  C -4.42030 1.27430 0.75320  C -4.87210 -0.19130 0.77790  C -4.63290 -0.93010 -0.54150  C -2.42960 -1.87700 0.09640  C -5.34380 -2.28400 -0.63150  C -6.82760 -2.12750 -0.93820  O -2.90650 -2.57970 0.96280  O -0.76410 1.64570 -1.30620  H -0.99790 3.32490 2.79570  H 0.64190 3.09830 2.18770  H -0.45420 1.71340 2.31600  H -2.94600 3.56570 1.25840  H -4.85350 -2.89530 -1.41710  H -5.19350 -2.85520 0.31030  H -7.34900 -1.56270 -0.15640  H -7.31610 -3.10710 -1.01050  H -6.99550 -1.60930 -1.88970  H -5.95380 -0.23540 1.01940  H -4.36940 -0.73670 1.60470  H -4.85680 1.80490 1.62160  H -4.82420 1.78810 -0.14460  H -2.48400 1.01660 1.71550  H -2.46450 0.78020 -0.04230  O -2.87930 3.34370 -0.72100  H -2.58550 2.72100 -1.43740  H -0.69510 4.11650 0.36980  H -4.84760 -0.28580 -1.42730  H 3.76820 0.93350 0.69050 |

**Structure 14-TS** (Figure S4, Figure 2 (reaction e))

| B3LYP/6-311+G(d) structure | PM6 structure |
| --- | --- |
| E(B3LYP/6-311+G(d)) = -1311.36482513 a.u. | E(PM6) = -0.25983734 a.u.  E^SP^(B3LYP/6-311+G(d,p)) = -1311.3400007 a.u.  E^SP^(O3LYP/6-311+G(d,p)) = -1310.8171895 a.u.  E^SP^(M062X/6-311+G(d,p)) = -1310.7923034 a.u. |
| C -1.69050 0.79890 1.33820  C -1.24520 1.76240 0.47120  H -1.78990 2.00400 -0.43350  C -1.21090 -1.96810 -0.34320  C -1.82770 -1.53380 -1.50590  C -3.19530 -1.32520 -1.68930  C -4.15800 -1.40630 -0.65270  C -5.64880 -1.37050 -0.98950  C -6.31520 -0.20440 -0.22210  C -5.38860 0.10340 0.95320  C -3.96470 0.14340 0.40240  C -2.87480 0.02490 1.30370  H -1.78780 -2.39410 0.47040  H -1.17470 -1.25260 -2.32970  H -3.51720 -0.91970 -2.64600  H -3.92510 -2.12610 0.12810  H -5.81720 -1.27730 -2.06490  H -6.11430 -2.31380 -0.68380  H -6.36210 0.67500 -0.87040  H -5.64720 1.05390 1.42780  H -5.47150 -0.67620 1.72210  H -3.01050 -0.66680 2.13410  O -7.67010 -0.43140 0.13390  H -7.72590 -1.19680 0.72090  C 0.21160 -1.89900 -0.21050  C 0.92860 -2.23810 0.88470  H 0.75670 -1.49300 -1.05790  H 0.45890 -2.63880 1.77720  H -1.03650 0.61490 2.18740  O 2.93060 -1.51340 -0.11550  C 0.01870 2.44440 0.74270  C 0.69400 3.31110 -0.33290  C 2.22930 3.02990 -0.36730  C 0.06150 3.26240 -1.72890  C 2.58710 1.56550 -0.67710  C 4.10160 1.33750 -0.75570  C 4.55240 -0.07950 -1.14870  C 4.35490 -1.18610 -0.11160  C 2.38670 -2.04210 1.00250  C 5.15830 -2.45810 -0.40990  C 6.65760 -2.31730 -0.13010  O 3.01380 -2.30810 2.00710  O 0.57750 2.33840 1.84090  H 0.64380 3.87040 -2.42690  H -0.95470 3.66520 -1.72900  H 0.01690 2.24880 -2.13540  H 2.64890 3.66340 -1.15770  H 4.75930 -3.26360 0.21140  H 4.98990 -2.74850 -1.45380  H 7.13530 -1.56590 -0.76460  H 7.17170 -3.26510 -0.31000  H 6.84050 -2.03870 0.91200  H 5.62350 -0.03680 -1.37140  H 4.06540 -0.39350 -2.08060  H 4.51960 2.03560 -1.49250  H 4.55360 1.61660 0.20050  H 2.12780 1.27650 -1.63170  H 2.14840 0.91900 0.08630  O 2.85290 3.48050 0.82940  H 2.32990 3.12000 1.56580  H 0.61420 4.34160 0.03870  H 4.60120 -0.82870 0.89200  H -3.84490 0.93260 -0.33960 | C -1.12600 0.34790 1.13270  C -0.99000 1.62010 0.71020  H -1.77950 2.16390 0.20010  C -1.53770 -1.11650 -1.14850  C -2.08580 -0.04830 -1.84330  C -3.45020 0.26300 -1.75950  C -4.27600 -0.47340 -0.90100  C -5.72860 -0.13390 -0.69020  C -6.07220 -0.40690 0.79070  C -4.76520 -0.61540 1.58670  C -3.60180 0.01490 0.84810  C -2.30110 -0.49430 0.99800  H -2.13980 -1.99010 -0.88970  H -1.43010 0.64900 -2.37290  H -3.82280 1.16260 -2.24640  H -4.06380 -1.55120 -0.78840  H -5.95080 0.91870 -0.94740  H -6.38180 -0.73640 -1.35350  H -6.70940 0.39510 1.22490  H -4.84990 -0.16280 2.59560  H -4.60410 -1.69430 1.77550  H -2.17630 -1.53120 1.31790  O -6.92040 -1.54910 0.91580  H -6.50110 -2.35420 0.55580  C -0.08730 -1.24960 -1.04110  C 0.54970 -2.38660 -0.72880  H 0.48900 -0.33010 -1.22170  H 0.05670 -3.34090 -0.54780  H -0.27200 -0.14470 1.62910  O 2.44960 -1.51500 0.27730  C 0.28440 2.33460 0.92320  C 0.85920 3.19950 -0.19760  C 2.37130 2.86970 -0.38740  C 0.10530 3.10730 -1.51740  C 2.62540 1.37610 -0.64850  C 4.12490 1.05710 -0.66050  C 4.41120 -0.44780 -0.72330  C 3.87930 -1.21660 0.48860  C 2.02970 -2.43420 -0.64860  C 4.64040 -2.50650 0.80280  C 5.94810 -2.22270 1.53110  O 2.78210 -3.14660 -1.27130  O 0.90540 2.23440 1.97020  H 0.62200 3.66610 -2.30890  H -0.90310 3.53100 -1.43520  H 0.00190 2.07010 -1.86110  H 2.80360 3.50110 -1.19770  H 3.99680 -3.16730 1.41890  H 4.82680 -3.08030 -0.13120  H 6.61840 -1.58990 0.93730  H 6.48630 -3.15470 1.74390  H 5.78100 -1.71750 2.49000  H 5.50690 -0.60320 -0.80590  H 3.99220 -0.88450 -1.65290  H 4.61060 1.55860 -1.52030  H 4.59720 1.50130 0.24270  H 2.16570 1.08100 -1.60930  H 2.13080 0.75570 0.12980  O 3.08780 3.32020 0.76090  H 2.72770 2.89900 1.59190  H 0.81460 4.26140 0.17270  H 3.77980 -0.56680 1.39160  H -3.70060 1.10840 0.75320 |

**Structure 15** (Figure S4, Figure 2 (reaction e))

| B3LYP/6-311+G(d) structure | PM6 structure |
| --- | --- |
| E(B3LYP/6-311+G(d)) = -1311.41620162 a.u. | E(PM6) = -0.35485161 a.u.  E^SP^(B3LYP/6-311+G(d,p)) = -1311.3793023 a.u.  E^SP^(O3LYP/6-311+G(d,p)) = -1310.8595873 a.u.  E^SP^(M062X/6-311+G(d,p)) = -1310.8471486 a.u. |
| C -1.50600 0.78020 1.40790  C -1.02390 1.63380 0.49570  H -1.34460 1.59350 -0.53730  C -1.73010 -1.72710 1.35650  C -2.63340 -2.90060 1.01540  C -3.82070 -2.79660 0.41340  C -4.40960 -1.46190 0.06670  C -5.21350 -1.27830 -1.22660  C -5.27730 0.26870 -1.41010  C -4.18850 0.85080 -0.45760  C -3.33730 -0.36370 -0.05510  C -2.48330 -0.34150 1.21540  H -1.41990 -1.82200 2.40420  H -2.23770 -3.88140 1.26850  H -4.40260 -3.69330 0.21040  H -5.07370 -1.17230 0.90030  H -4.68560 -1.74000 -2.06820  H -6.21670 -1.70980 -1.19700  H -5.06680 0.53580 -2.44630  H -3.63010 1.66240 -0.92510  H -4.67520 1.27880 0.42810  H -3.17140 -0.30220 2.07280  O -6.57250 0.82520 -1.18460  H -6.81480 0.71180 -0.25670  C -0.48790 -1.79610 0.50080  C 0.76440 -1.80010 0.96140  H -0.63210 -1.84070 -0.57650  H 0.98700 -1.73710 2.02200  H -1.10200 0.87420 2.41550  O 3.03820 -1.43850 0.69670  C 0.03160 2.61020 0.86740  C 0.76590 3.41050 -0.21150  C 2.28130 3.02520 -0.19460  C 0.16490 3.37390 -1.62010  C 2.54500 1.53130 -0.43560  C 4.03470 1.21150 -0.61410  C 4.35400 -0.22560 -1.06630  C 4.33080 -1.31480 0.01130  C 1.92250 -1.79530 0.03020  C 4.81000 -2.69200 -0.46690  C 6.31740 -2.75290 -0.73460  O 1.86720 -2.04290 -1.15460  O 0.37370 2.76130 2.03620  H 0.72580 4.04090 -2.28030  H -0.87280 3.71860 -1.62390  H 0.18830 2.37900 -2.07070  H 2.75600 3.59950 -0.99860  H 4.55770 -3.41670 0.31470  H 4.25230 -2.98550 -1.35890  H 6.61750 -2.12100 -1.57440  H 6.62040 -3.77470 -0.97750  H 6.89620 -2.43960 0.14050  H 5.37680 -0.22880 -1.45850  H 3.70860 -0.51900 -1.89810  H 4.44020 1.89190 -1.37360  H 4.57040 1.44900 0.31050  H 2.00180 1.20680 -1.33150  H 2.13760 0.96150 0.40390  O 2.90760 3.47930 0.99970  H 2.31540 3.28160 1.74310  H 0.74310 4.44750 0.14360  H 4.96830 -0.99320 0.84040  H -2.69590 -0.62880 -0.90710 | C -1.35880 0.47690 1.36910  C -0.99190 1.44770 0.52270  H -1.45740 1.58700 -0.45040  C -1.76430 -1.98030 1.05470  C -2.65820 -3.03690 0.43920  C -3.86310 -2.80020 -0.09080  C -4.45910 -1.43100 -0.01550  C -5.43810 -0.97360 -1.10490  C -5.35980 0.57510 -1.06580  C -4.10900 0.95540 -0.22900  C -3.32920 -0.35850 -0.07630  C -2.42330 -0.55860 1.14730  H -1.52800 -2.30150 2.10350  H -2.21940 -4.03570 0.43840  H -4.46760 -3.57860 -0.55000  H -4.98590 -1.36180 0.97410  H -5.15210 -1.35690 -2.09860  H -6.46740 -1.32530 -0.92860  H -5.35370 1.02760 -2.07890  H -3.51580 1.74040 -0.72340  H -4.40510 1.38190 0.74480  H -3.07340 -0.55320 2.06480  O -6.54340 1.13630 -0.49850  H -6.69640 0.81150 0.40920  C -0.49760 -1.92570 0.23220  C 0.72460 -1.86040 0.76960  H -0.64370 -1.94320 -0.85450  H 0.91820 -1.82310 1.84390  H -0.84630 0.38250 2.34040  O 2.95510 -1.27730 0.64970  C 0.10230 2.37630 0.89400  C 0.71610 3.30870 -0.14550  C 2.24000 3.00410 -0.27060  C 0.04600 3.30660 -1.51200  C 2.52900 1.51110 -0.47820  C 3.99620 1.27190 -0.85060  C 4.28840 -0.20120 -1.15240  C 4.30390 -1.09690 0.08550  C 1.93140 -1.81190 -0.08540  C 4.89420 -2.48830 -0.17310  C 6.41590 -2.45670 -0.23420  O 2.05230 -2.16730 -1.23790  O 0.54860 2.39990 2.03130  H 0.51410 4.04360 -2.17920  H -1.01570 3.57330 -1.44350  H 0.12080 2.33140 -2.00970  H 2.68740 3.62810 -1.07880  H 4.56070 -3.17610 0.63100  H 4.47310 -2.90970 -1.11060  H 6.77940 -1.81690 -1.04740  H 6.82100 -3.46150 -0.40750  H 6.85490 -2.08610 0.69990  H 5.27360 -0.27960 -1.65760  H 3.55620 -0.59620 -1.89080  H 4.26410 1.88080 -1.73710  H 4.64550 1.64100 -0.02970  H 1.86510 1.10080 -1.26250  H 2.29490 0.94110 0.44720  O 2.90760 3.49390 0.89220  H 2.52360 3.09270 1.71660  H 0.63920 4.34690 0.28660  H 4.77930 -0.59480 0.96170  H -2.71860 -0.51590 -0.99890 |
| **Structure 16** (Figure S5) | **Structure 17-TS** (Figure S5) |
| PM6 structure | PM6 structure |
| E(PM6) = -0.53550312 a.u.  E^SP^(B3LYP/6-311+G(d,p)) = -1843.31645861 a.u.  E^SP^(O3LYP/6-311+G(d,p)) = -1842.59402318 a.u.  E^SP^(M062X/6-311+G(d,p)) = -1842.55203055 a.u. | E(PM6) = -0.48194613 a.u.  E^SP^(B3LYP/6-311+G(d,p)) = -1843.27173802 a.u.  E^SP^(O3LYP/6-311+G(d,p)) = -1842.55237512 a.u.  E^SP^(M062X/6-311+G(d,p)) = -1842.50776337 a.u. |
| C -3.12300 -2.61540 1.88690  C -3.84060 -2.69280 0.75420  H -4.93820 -2.73240 0.82550  C -0.91040 1.19100 1.50340  C -1.01490 0.92010 0.19480  C -0.48660 -0.29840 -0.41620  C 0.71700 -0.82700 -0.16030  C -0.77200 -3.22400 1.36630  C -1.67560 -2.51720 2.05890  H -0.37760 0.51870 2.18380  H -1.55830 1.59300 -0.48810  H -1.18900 -0.80920 -1.08980  H 1.44030 -0.34430 0.49530  H -1.37240 -1.81290 2.83840  C -1.49370 2.36470 2.15540  C -2.79100 2.69120 2.16740  H -0.76560 2.99370 2.68570  H -3.66980 -2.58810 2.84380  O -4.82790 2.60540 0.82400  C -3.34880 -2.67150 -0.63210  C -4.37930 -2.37530 -1.72870  C -5.03770 -0.97830 -1.60020  C -3.99570 0.15150 -1.59040  C -4.66540 1.50840 -1.83600  C -3.80570 2.69810 -1.38160  C -4.50240 3.49970 -0.27560  C -3.88290 1.90200 1.56220  C -3.71990 4.73180 0.18970  C -3.89600 5.89810 -0.77730  O -4.14160 0.73580 1.74610  O -2.19830 -2.89150 -0.97160  H -5.77820 -0.85600 -2.42720  H -4.08040 5.02550 1.19760  H -2.64380 4.49920 0.30650  H -3.52710 5.65830 -1.78140  H -3.34490 6.78130 -0.43120  H -4.94840 6.19310 -0.87400  H -3.59730 3.36700 -2.23890  H -2.80450 2.35350 -1.03540  H -4.91970 1.61360 -2.90680  H -5.63960 1.52080 -1.29210  H -3.20790 -0.03360 -2.34290  H -3.48490 0.14960 -0.60230  O -5.86770 -0.90920 -0.43740  H -5.37600 -0.53340 0.34110  H -5.54640 3.77940 -0.57020  H -1.07430 -3.92170 0.57620  C -5.44730 -3.46870 -1.74630  H -6.03010 -3.43810 -2.67400  H -5.00970 -4.46960 -1.66390  H -6.15870 -3.33580 -0.91620  H -3.82900 -2.39920 -2.70630  H -3.15090 3.57820 2.69920  C 1.15180 -2.13140 -0.75030  C 1.50630 -3.19340 0.30510  C 0.70010 -3.12180 1.61070  H 0.35030 -2.54650 -1.40610  H 2.03380 -1.96630 -1.41210  H 1.43430 -4.20930 -0.14980  H 1.02870 -3.94860 2.27980  H 0.96790 -2.19500 2.15800  O 2.87650 -3.03240 0.74250  H 3.48890 -3.03910 -0.05290  C 9.33340 1.14210 -0.04900  C 8.02980 1.25070 -0.84560  C 6.98350 0.33790 -0.17090  C 5.65960 0.33750 -0.93380  C 4.72340 -0.74060 -0.42080  O 4.41690 -1.72300 -1.09110  O 9.56390 1.43890 1.09690  O 10.30320 0.58570 -0.84180  N 7.70970 2.69480 -0.88220  N 4.17100 -0.57200 0.83770  H 8.21830 0.90360 -1.90210  H 7.38420 -0.69900 -0.11930  H 6.83160 0.64870 0.88280  H 5.16470 1.32720 -0.88820  H 5.83780 0.14100 -2.01700  H 11.18510 0.51030 -0.38460  H 6.95000 2.88650 -1.52680  H 7.44780 3.04630 0.03570  H 3.55010 -1.31320 1.21010  H 4.44770 0.15370 1.47120 | C 1.61830 -0.86990 -1.64870  C 2.75770 -1.56660 -1.42860  H 3.59340 -1.34320 -2.12360  C 0.99330 1.27900 0.14370  C 1.80320 0.61240 1.04660  C 1.31350 -0.45370 1.81180  C -0.02660 -0.87270 1.62490  C -0.07290 -1.71950 0.02830  C 0.33010 -0.84360 -1.00450  H -0.09850 1.17630 0.22410  H 2.87110 0.87150 1.12100  H 1.98500 -1.03040 2.44300  H -0.74740 -0.05170 1.40110  H -0.42880 -0.22480 -1.48760  C 1.40370 2.41720 -0.66080  C 2.57760 2.72100 -1.23410  H 0.59430 3.16300 -0.76870  H 1.67510 -0.15600 -2.49780  O 4.99990 2.30320 -1.01680  C 3.13820 -2.56830 -0.42890  C 4.62850 -2.92890 -0.32310  C 5.59430 -1.72270 -0.18340  C 5.06420 -0.68570 0.82180  C 5.99440 0.52780 0.93860  C 5.24360 1.80580 1.34910  C 5.32710 2.89390 0.27260  C 3.75860 1.86870 -1.43120  C 4.49080 4.13950 0.57500  C 5.17030 5.02640 1.61300  O 3.79070 0.87320 -2.12640  O 2.37410 -3.16810 0.31190  H 6.60270 -2.09970 0.11370  H 4.34240 4.71100 -0.36470  H 3.47620 3.86110 0.92240  H 5.29430 4.51220 2.57340  H 4.57790 5.93000 1.80400  H 6.16330 5.35560 1.28300  H 5.65710 2.20390 2.29570  H 4.17700 1.57660 1.57420  H 6.80750 0.31570 1.65530  H 6.49790 0.68470 -0.04610  H 4.91790 -1.15970 1.80930  H 4.05650 -0.34700 0.49580  O 5.87250 -1.12890 -1.44850  H 5.14330 -0.50840 -1.73940  H 6.39170 3.16600 0.05410  H 0.63460 -2.56300 0.23950  C 5.01750 -3.79120 -1.52380  H 5.98970 -4.27220 -1.36400  H 4.28240 -4.58000 -1.71610  H 5.11700 -3.17890 -2.43340  H 4.73580 -3.54690 0.60800  H 2.70090 3.68910 -1.73950  C -0.67390 -1.94870 2.47330  C -1.78100 -2.58290 1.60040  C -1.51810 -2.18990 0.12780  H 0.05660 -2.71630 2.79180  H -1.10180 -1.52960 3.40330  H -1.86070 -3.67880 1.75200  H -1.68240 -3.04430 -0.55210  H -2.23710 -1.40500 -0.18800  O -3.07070 -2.11780 2.01840  H -3.16060 -1.13210 1.85750  C -9.55550 1.43900 -0.03520  C -8.31940 1.19320 -0.90550  C -7.36210 0.27030 -0.12140  C -6.05100 0.04760 -0.87480  C -5.03250 -0.66250 -0.00310  O -4.03760 -0.09790 0.44700  O -10.38260 0.65990 0.36800  O -9.61420 2.76670 0.30130  N -8.83650 0.66180 -2.18570  N -5.22640 -2.00900 0.24790  H -7.81720 2.18190 -1.11150  H -7.14810 0.72510 0.87150  H -7.86000 -0.69610 0.09830  H -6.21490 -0.51410 -1.81510  H -5.61180 1.02510 -1.18200  H -10.41420 2.99500 0.84940  H -8.10320 0.60650 -2.88450  H -9.24340 -0.26390 -2.07360  H -4.56780 -2.50030 0.87480  H -6.05330 -2.50910 -0.02130 |

| **Structure 18** (Figure S5) | **Structure 19** (Figure S6) |
| --- | --- |
| PM6 structure | PM6 structure |
| E(PM6) = -0.56276688 a.u.  E^SP^(B3LYP/6-311+G(d,p)) = -1843.30794105 a.u.  E^SP^(O3LYP/6-311+G(d,p)) = -1842.58994553 a.u.  E^SP^(M062X/6-311+G(d,p)) = -1842.56499377 a.u. | E(PM6) = -0.77365538 a.u.  E^SP^(B3LYP/6-311+G(d,p)) = -2242.39741056 a.u.  E^SP^(O3LYP/6-311+G(d,p)) = -2241.51933658 a.u.  E^SP^(M062X/6-311+G(d,p)) = -2241.48768776 a.u. |
| C -1.34150 0.22630 -1.50240  C -1.75590 1.48990 -1.61450  H -2.29020 1.78860 -2.53500  C -1.39980 -1.81720 0.04240  C -2.22750 -1.62980 1.29350  C -1.66730 -1.04250 2.36110  C -0.27920 -0.51820 2.16170  C -0.30890 0.33220 0.84920  C -0.63850 -0.51070 -0.39880  H -0.55060 -2.50190 0.36500  H -3.24840 -2.01100 1.28500  H -2.15530 -0.90120 3.31620  H 0.41860 -1.38680 2.03560  H 0.33610 -0.85720 -0.84550  C -2.14660 -2.60160 -1.00400  C -3.28570 -2.39720 -1.66820  H -1.65490 -3.57550 -1.17930  H -1.53130 -0.43290 -2.36900  O -5.43910 -1.18430 -1.44680  C -1.59800 2.64560 -0.70400  C -2.83250 3.30670 -0.08760  C -4.18540 2.55390 -0.13490  C -4.16080 1.20000 0.59240  C -5.57510 0.60660 0.66990  C -5.58390 -0.89860 0.97370  C -6.01140 -1.74310 -0.23160  C -4.12290 -1.19340 -1.83600  C -5.71930 -3.23910 -0.09250  C -6.71300 -3.91380 0.84690  O -3.80710 -0.23700 -2.51590  O -0.50750 3.14460 -0.49440  H -4.96570 3.22320 0.31040  H -5.77380 -3.71070 -1.09600  H -4.68540 -3.41230 0.26490  H -6.65760 -3.50730 1.86360  H -6.51650 -4.99110 0.91770  H -7.74750 -3.79820 0.50020  H -6.27910 -1.10830 1.81090  H -4.58790 -1.22750 1.34240  H -6.16410 1.14690 1.43410  H -6.09520 0.79970 -0.29860  H -3.73550 1.31470 1.60580  H -3.47950 0.50250 0.06470  O -4.64970 2.42730 -1.47710  H -4.32920 1.59000 -1.90890  H -7.09030 -1.56530 -0.48070  H -1.11470 1.09590 0.98130  C -3.00030 4.66790 -0.77610  H -3.69670 5.31040 -0.22680  H -2.04510 5.20270 -0.85300  H -3.40960 4.54050 -1.79000  H -2.58390 3.48210 0.99240  H -3.66570 -3.19950 -2.32420  C 0.33540 0.43650 3.19150  C 1.34320 1.28810 2.37360  C 1.05070 1.04370 0.87460  H -0.42290 1.07700 3.66860  H 0.84820 -0.09770 4.00850  H 1.31840 2.35960 2.65590  H 1.03000 1.98830 0.29580  H 1.85000 0.42620 0.42160  O 2.68610 0.91080 2.70200  H 2.89110 -0.00900 2.35930  C 9.47020 -0.99040 -0.50100  C 8.11180 -0.83370 -1.19010  C 7.11470 -0.27000 -0.15560  C 5.70010 -0.18180 -0.72720  C 4.69180 0.16000 0.35480  O 3.87480 -0.65560 0.77770  O 10.20070 -0.15380 -0.03200  O 9.79200 -2.32210 -0.43480  N 8.36590 0.01020 -2.37900  N 4.69200 1.45000 0.84940  H 7.76530 -1.84660 -1.54500  H 7.11120 -0.92450 0.74440  H 7.45720 0.72190 0.20360  H 5.64210 0.55180 -1.55470  H 5.40150 -1.15940 -1.17120  H 10.67980 -2.48780 -0.01510  H 7.55020 0.05130 -2.98100  H 8.61920 0.96080 -2.11900  H 4.01910 1.70330 1.59310  H 5.34490 2.15560 0.56520 | C 0.82420 1.94890 2.01710  C 1.88360 2.51320 1.41290  H 2.86190 2.45670 1.91200  C -0.46960 -1.05220 -1.02400  C 0.00620 -0.19570 -1.93830  C -0.43370 1.19460 -2.03470  C -1.69620 1.61200 -1.87420  C -1.25180 2.87440 1.01220  C -0.56200 1.86770 1.56700  H -1.27640 -0.76840 -0.33760  H 0.80800 -0.48890 -2.63360  H 0.37670 1.91760 -2.19080  H -2.52990 0.91910 -1.73280  H -1.02470 0.89220 1.74520  C 0.03360 -2.41290 -0.82540  C 1.27150 -2.72290 -0.42230  H -0.70450 -3.20490 -1.01080  H 0.99210 1.43680 2.98020  O 3.57120 -1.88040 -0.64880  C 1.92790 3.18090 0.10460  C 3.31600 3.37190 -0.52650  C 4.02080 2.04670 -0.89800  C 3.19840 1.19980 -1.88180  C 4.08230 0.20410 -2.64390  C 3.38090 -1.12700 -2.95370  C 3.86000 -2.25050 -2.02900  C 2.31750 -1.74570 -0.07500  C 3.32110 -3.63570 -2.39030  C 4.13040 -4.26410 -3.52030  O 2.26960 -0.87190 0.76540  O 0.96200 3.61410 -0.49870  H 5.04030 2.27840 -1.29240  H 3.36860 -4.28800 -1.49290  H 2.25240 -3.58690 -2.67680  H 4.08870 -3.66620 -4.43830  H 3.74980 -5.26340 -3.76650  H 5.18790 -4.38050 -3.25060  H 3.58020 -1.42030 -4.00410  H 2.27450 -1.01370 -2.89140  H 4.43820 0.66710 -3.58360  H 5.00690 0.00220 -2.04710  H 2.65120 1.84520 -2.59460  H 2.41370 0.66420 -1.30460  O 4.33000 1.26350 0.26460  H 3.52110 0.77840 0.60180  H 4.98180 -2.26370 -1.94790  H -0.78970 3.84810 0.81540  C 4.20060 4.20960 0.39640  H 5.08320 4.58620 -0.13360  H 3.66430 5.07730 0.79790  H 4.56490 3.61260 1.24600  H 3.15880 3.94450 -1.48000  H 1.57730 -3.76100 -0.25330  C -2.04770 3.06700 -1.84920  C -3.01930 3.42590 -0.71390  C -2.69210 2.74810 0.63060  H -1.12520 3.68720 -1.73750  H -2.49700 3.36460 -2.82060  H -3.10200 4.53120 -0.60290  H -3.32400 3.19160 1.42840  H -3.00600 1.67980 0.57580  O -4.36040 3.05570 -1.08690  H -4.38830 2.08850 -1.35020  C -9.82280 -2.22350 0.26250  C -8.45940 -2.09760 0.94900  C -7.81220 -0.77650 0.48110  C -6.40020 -0.61090 1.04220  C -5.68860 0.56270 0.39640  O -4.76290 0.42060 -0.39960  O -10.80080 -1.52460 0.35520  O -9.80570 -3.30430 -0.58080  N -8.73460 -2.21330 2.39790  N -6.08480 1.83710 0.76290  H -7.81550 -2.97100 0.64160  H -7.77500 -0.76220 -0.63100  H -8.45320 0.08280 0.76570  H -6.41310 -0.49780 2.14390  H -5.79580 -1.52600 0.84200  H -10.67700 -3.45430 -1.04000  H -7.87530 -2.29470 2.93090  H -9.25560 -1.41310 2.74880  H -5.63890 2.65040 0.30660  H -6.88260 2.02380 1.34180  C 5.36980 -1.46140 2.00000  C 5.69140 -1.38340 3.48380  C 6.39990 -0.63810 1.18400  O 6.13020 -0.81220 -0.20540  O 5.02400 -0.96410 4.39600  O 6.96730 -1.84830 3.73110  N 5.31060 -2.89530 1.62340  H 4.33470 -1.02650 1.84400  H 6.35180 0.43630 1.44060  H 7.43030 -1.01940 1.30890  H 5.26060 -0.34380 -0.43240  H 7.19600 -1.84350 4.69700  H 4.97280 -2.96150 0.65810  H 6.24490 -3.30280 1.64080 |

| **Structure 20-TS** (Figure S6) | **Structure 21** (Figure S6) |
| --- | --- |
| PM6 structure | PM6 structure |
| E(PM6) = -0.71625968 a.u.  E^SP^(B3LYP/6-311+G(d,p)) = -2242.35441277 a.u.  E^SP^(O3LYP/6-311+G(d,p)) = -2241.48159977 a.u.  E^SP^(M062X/6-311+G(d,p)) = -2241.43919423 a.u. | E(PM6) = -0.79656815 a.u.  E^SP^(B3LYP/6-311+G(d,p)) = -2242.39266535 a.u.  E^SP^(O3LYP/6-311+G(d,p)) = -2241.52123266 a.u.  E^SP^(M062X/6-311+G(d,p)) = -2241.49752139 a.u. |
| C -0.56260 0.51990 -1.34780  C -1.63080 1.35250 -1.38930  H -2.55590 0.90940 -1.81130  C 0.19970 -0.53180 1.20610  C -0.42530 0.57950 1.74340  C 0.22890 1.81580 1.82030  C 1.54320 1.93280 1.30530  C 1.38100 1.89320 -0.49250  C 0.79750 0.67320 -0.90560  H 1.29460 -0.53670 1.10270  H -1.47970 0.51900 2.05560  H -0.30870 2.70060 2.15360  H 2.18260 1.02890 1.43890  H 1.45510 -0.17910 -1.09070  C -0.39040 -1.85820 1.12230  C -1.65320 -2.25230 0.90310  H 0.35070 -2.65670 1.31380  H -0.76630 -0.50380 -1.72950  O -3.96670 -1.40010 1.15570  C -1.83000 2.74160 -0.97630  C -3.28050 3.25830 -0.92560  C -4.24410 2.40090 -0.07020  C -3.60250 1.99090 1.26530  C -4.55430 1.17780 2.15030  C -3.81510 0.14590 3.01850  C -4.12860 -1.29460 2.60140  C -2.79690 -1.46230 0.43370  C -3.33810 -2.35820 3.36280  C -3.90380 -2.57210 4.76280  O -2.89640 -0.97430 -0.67780  O -0.95420 3.54100 -0.68600  H -5.20140 2.95410 0.08310  H -3.37610 -3.31300 2.79700  H -2.26540 -2.08770 3.42670  H -3.83840 -1.66560 5.37610  H -3.35250 -3.36030 5.29140  H -4.95700 -2.87920 4.73710  H -4.09400 0.27660 4.08290  H -2.71580 0.32270 2.98510  H -5.15060 1.85470 2.78930  H -5.30100 0.65460 1.49850  H -3.25140 2.89110 1.80400  H -2.68830 1.39230 1.05600  O -4.69410 1.24480 -0.78810  H -3.94190 0.58760 -0.92660  H -5.23120 -1.49640 2.64780  H 0.74650 2.80300 -0.65060  C -3.81000 3.42100 -2.34920  H -4.74300 3.99740 -2.36000  H -3.09150 3.94230 -2.99240  H -4.03560 2.44720 -2.80790  H -3.23260 4.27470 -0.44880  H -1.90720 -3.32240 0.95110  C 2.36020 3.20230 1.43660  C 3.34910 3.20700 0.24820  C 2.84220 2.19200 -0.80250  H 1.72340 4.10670 1.41530  H 2.90610 3.23590 2.39840  H 3.50480 4.22400 -0.16670  H 2.93340 2.59370 -1.82730  H 3.47080 1.27680 -0.77660  O 4.66280 2.84930 0.69380  H 4.67600 1.90600 1.03510  C 10.51130 -2.08270 0.03320  C 9.17090 -2.13170 -0.70650  C 8.41050 -0.82400 -0.39830  C 7.01360 -0.82470 -1.01850  C 6.19320 0.35020 -0.52150  O 5.24570 0.21730 0.25090  O 11.43710 -1.32150 -0.09760  O 10.54110 -3.07380 0.97980  N 9.51200 -2.37030 -2.12600  N 6.51170 1.60820 -1.00080  H 8.58100 -3.01700 -0.33190  H 8.32830 -0.70100 0.70460  H 8.99780 0.04900 -0.74930  H 7.06220 -0.82280 -2.12510  H 6.47020 -1.75870 -0.74410  H 11.40240 -3.11010 1.47930  H 8.68360 -2.57270 -2.67540  H 9.98540 -1.57110 -2.54080  H 5.99330 2.42620 -0.64180  H 7.31660 1.79670 -1.56930  C -5.88950 -1.89070 -1.59870  C -6.16370 -2.49050 -2.96880  C -6.92160 -0.77760 -1.28080  O -6.76260 -0.37350 0.07550  O -5.44380 -2.57470 -3.93200  O -7.46140 -2.95540 -3.04700  N -5.88590 -3.00390 -0.61740  H -4.84670 -1.44730 -1.61680  H -6.79890 0.09110 -1.95170  H -7.96140 -1.15430 -1.32740  H -5.90850 0.17130 0.16930  H -7.66180 -3.38560 -3.91870  H -5.55300 -2.64190 0.28150  H -6.83620 -3.34160 -0.46670 | C 0.40030 -0.79010 -0.26000  C 0.88870 -1.34540 0.85220  H 1.57350 -2.20630 0.74200  C 0.06930 1.42050 -1.51710  C 0.67010 2.63600 -0.84830  C -0.02250 3.25990 0.11530  C -1.31380 2.61520 0.50990  C -1.01040 1.10250 0.76530  C -0.52050 0.37190 -0.50030  H -0.86050 1.81380 -2.03980  H 1.63750 2.98230 -1.21700  H 0.29710 4.16000 0.62370  H -2.03640 2.71640 -0.34280  H -1.42450 -0.07250 -1.00770  C 0.95650 0.91630 -2.62470  C 2.22340 0.49770 -2.63740  H 0.45400 0.99420 -3.60420  H 0.70680 -1.26120 -1.21330  O 4.25080 0.79310 -1.21380  C 0.65770 -1.04270 2.28110  C 1.85750 -0.73150 3.18300  C 3.11760 -0.10590 2.54530  C 2.82980 1.16990 1.74110  C 4.13690 1.85410 1.31550  C 3.95150 2.80520 0.12560  C 4.48650 2.23210 -1.19310  C 3.11490 0.10100 -1.53370  C 3.95760 2.93620 -2.44200  C 4.62980 4.28940 -2.64320  O 3.03400 -0.97740 -0.97030  O -0.43900 -1.13150 2.79820  H 3.86370 0.09090 3.35660  H 4.13750 2.29440 -3.32970  H 2.85370 3.06660 -2.37930  H 4.43400 4.97490 -1.81030  H 4.26390 4.77680 -3.55620  H 5.71840 4.19520 -2.74540  H 4.48300 3.75860 0.32450  H 2.88450 3.09070 0.02480  H 4.56850 2.40440 2.17260  H 4.89300 1.06890 1.05550  H 2.20920 1.86640 2.33500  H 2.22030 0.92210 0.84760  O 3.82210 -1.06390 1.74340  H 3.36950 -1.20580 0.86140  H 5.60750 2.19940 -1.18550  H -0.20090 1.05760 1.53440  C 2.23730 -2.03810 3.88970  H 2.92090 -1.85300 4.72650  H 1.35600 -2.55240 4.29340  H 2.75390 -2.72270 3.20100  H 1.48410 -0.00690 3.95630  H 2.70530 0.26550 -3.60390  C -2.03350 3.05460 1.79000  C -2.84570 1.80300 2.21900  C -2.31780 0.60330 1.39740  H -1.32900 3.35610 2.58080  H -2.70130 3.91630 1.62450  H -2.81500 1.63250 3.31360  H -2.15340 -0.29170 2.02910  H -3.06010 0.30460 0.63240  O -4.24150 2.01540 1.97530  H -4.42900 2.04850 0.99050  C -10.50320 -1.10300 -1.29500  C -9.05330 -1.58480 -1.39700  C -8.20240 -0.74430 -0.42130  C -6.71670 -1.08520 -0.53210  C -5.86930 -0.10670 0.26030  O -5.14930 0.73150 -0.27910  O -11.26490 -1.13610 -0.36090  O -10.87750 -0.54100 -2.48870  N -9.10080 -3.04030 -1.13480  N -5.89970 -0.19850 1.63860  H -8.69200 -1.42890 -2.45400  H -8.35300 0.33610 -0.64140  H -8.56290 -0.88560 0.61810  H -6.51400 -2.12400 -0.20640  H -6.38750 -1.03450 -1.59570  H -11.82310 -0.22860 -2.49280  H -8.20560 -3.47770 -1.32650  H -9.35540 -3.24170 -0.17050  H -5.34250 0.46850 2.19900  H -6.48940 -0.83480 2.14130  C 5.99760 -2.20870 -0.82010  C 6.33590 -3.61180 -1.30050  C 6.63000 -1.94820 0.57120  O 6.43940 -0.58090 0.91900  O 5.60380 -4.50920 -1.63450  O 7.70140 -3.81380 -1.29240  N 6.44490 -1.26220 -1.87140  H 4.86940 -2.13070 -0.74630  H 6.19300 -2.60330 1.34470  H 7.73090 -2.06730 0.55090  H 5.46780 -0.42200 1.16880  H 7.95800 -4.70930 -1.63530  H 6.07390 -0.33290 -1.65210  H 7.46150 -1.18520 -1.86590 |

| **Structure 22** (Figure S7) | **Structure 23-TS** (Figure S7) |
| --- | --- |
| PM6 structure | PM6 structure |
| E(PM6) = -1.00478653 a.u.  E^SP^(B3LYP/6-311+G(d,p)) = -2641.47985089 a.u.  E^SP^(O3LYP/6-311+G(d,p)) = -2640.44948306 a.u.  E^SP^(M062X/6-311+G(d,p)) = -2640.41620849 a.u. | E(PM6) = -0.94956272 a.u.  E^SP^(B3LYP/6-311+G(d,p)) = -2641.43982897 a.u.  E^SP^(O3LYP/6-311+G(d,p)) = -2640.41240350 a.u.  E^SP^(M062X/6-311+G(d,p)) = -2640.37678788 a.u. |
| C 0.66330 0.69400 1.90180  C 1.70410 1.40360 1.42580  H 2.67260 1.30710 1.94310  C 0.10790 -2.18230 -1.12560  C 0.44180 -1.30350 -2.08150  C -0.18840 0.00240 -2.25110  C -1.42990 0.30690 -1.84330  C -1.44000 1.62540 0.94510  C -0.70600 0.60490 1.41220  H -0.71540 -1.99440 -0.42700  H 1.27150 -1.51500 -2.77500  H 0.45080 0.76000 -2.71190  H -2.09920 -0.44450 -1.41580  H -1.13060 -0.40270 1.48140  C 0.79810 -3.45520 -0.90240  C 2.04940 -3.57800 -0.44550  H 0.19430 -4.34670 -1.12060  H 0.85010 0.04230 2.77460  O 4.17860 -2.34780 -0.60020  C 1.75140 2.28300 0.26170  C 3.13110 2.70790 -0.25180  C 4.03230 1.53220 -0.70110  C 3.36370 0.64460 -1.76220  C 4.40300 -0.16950 -2.54600  C 3.94040 -1.59090 -2.89940  C 4.56420 -2.64180 -1.97620  C 2.91440 -2.45330 -0.05090  C 4.26490 -4.08760 -2.37390  C 5.19370 -4.55520 -3.49000  O 2.70330 -1.62890 0.81620  O 0.76850 2.74210 -0.31430  H 5.00760 1.94830 -1.05570  H 4.39370 -4.74230 -1.48620  H 3.20990 -4.20600 -2.68980  H 5.07750 -3.95610 -4.40070  H 4.98770 -5.59870 -3.76010  H 6.24910 -4.50250 -3.19340  H 4.22390 -1.82570 -3.94570  H 2.82920 -1.66190 -2.88000  H 4.67660 0.37360 -3.47060  H 5.34850 -0.23070 -1.95160  H 2.75740 1.25440 -2.45790  H 2.63900 -0.02780 -1.25410  O 4.44510 0.72950 0.41320  H 3.72270 0.08720 0.68970  H 5.67130 -2.47670 -1.85710  H -1.03670 2.64920 0.87300  C 3.83570 3.54110 0.81970  H 4.68510 4.09200 0.39770  H 3.16430 4.27630 1.27760  H 4.23530 2.90060 1.62130  H 2.96590 3.35980 -1.15240  H 2.50310 -4.55830 -0.26280  C -1.97910 1.69510 -1.92610  C -3.11120 1.98330 -0.92570  C -2.85810 1.46660 0.50460  H -1.15990 2.43350 -1.76400  H -2.36200 1.88180 -2.95430  H -3.35620 3.07430 -0.91290  H -3.51940 2.02620 1.20500  H -3.19590 0.40670 0.57010  O -4.33360 1.40730 -1.41390  H -4.25420 0.40950 -1.47380  C -9.88240 -3.64230 0.24980  C -8.62200 -3.34190 1.06630  C -7.92320 -2.12120 0.43060  C -6.59600 -1.80890 1.12110  C -5.81200 -0.76180 0.35230  O -4.78900 -1.03150 -0.27540  O -10.86880 -2.97110 0.07650  O -9.74790 -4.86590 -0.35450  N -9.08530 -3.17610 2.46160  N -6.25950 0.54470 0.40280  H -7.94000 -4.23930 1.02570  H -7.74220 -2.32450 -0.64840  H -8.59740 -1.24070 0.45520  H -6.75090 -1.48480 2.16870  H -5.96690 -2.72710 1.18130  H -10.55010 -5.12980 -0.88280  H -8.30290 -3.12330 3.10530  H -9.65060 -2.33770 2.57460  H -5.75810 1.26960 -0.13960  H -7.11510 0.82190 0.84700  C 5.92480 -1.84830 2.03760  C 6.23590 -1.79140 3.52490  C 6.83880 -0.86540 1.26090  O 6.56580 -0.98620 -0.13340  O 5.52190 -1.49620 4.44990  O 7.56000 -2.10390 3.75680  N 6.04910 -3.26110 1.60170  H 4.84290 -1.54350 1.89550  H 6.67360 0.17890 1.58340  H 7.90840 -1.13130 1.35130  H 5.64010 -0.61640 -0.32100  H 7.78860 -2.10990 4.72300  H 5.73280 -3.32820 0.62990  H 7.02630 -3.55120 1.61940  C -0.98380 5.21660 0.14790  C -2.44950 5.53150 0.39390  C -0.61730 5.52300 -1.32650  O 0.75130 5.20360 -1.54560  O -3.36420 4.76390 0.58190  O -2.68730 6.88200 0.33150  N -0.16670 5.94600 1.14500  H -0.83090 4.09850 0.34380  H -1.25410 4.95940 -2.03100  H -0.67510 6.60520 -1.55520  H 0.90800 4.22340 -1.37660  H -3.63570 7.11710 0.52310  H 0.81980 5.73530 0.98610  H -0.27780 6.95380 1.03080 | C 0.68570 0.13090 0.83740  C 1.69050 1.02220 0.62110  H 2.61590 0.82770 1.20400  C 0.23850 -1.87380 -1.13980  C 0.80950 -0.99560 -2.04210  C 0.06260 0.04260 -2.61630  C -1.29470 0.21080 -2.24180  C -1.26670 0.88910 -0.58490  C -0.63610 -0.01400 0.30890  H -0.85820 -1.94420 -1.07280  H 1.88460 -1.06390 -2.27040  H 0.54840 0.77610 -3.25630  H -1.84500 -0.73850 -2.03740  H -1.25610 -0.78190 0.77870  C 0.92100 -3.00830 -0.53370  C 2.18310 -3.16080 -0.11130  H 0.26150 -3.89370 -0.45600  H 0.93770 -0.66320 1.57440  O 4.43850 -2.22960 -0.54430  C 1.81590 2.20050 -0.21350  C 3.21900 2.78280 -0.44880  C 4.30950 1.75900 -0.84680  C 3.81170 0.80490 -1.94460  C 4.90010 -0.16880 -2.41130  C 4.32720 -1.52290 -2.86110  C 4.71640 -2.65980 -1.91090  C 3.22130 -2.14060 0.08700  C 4.08320 -4.00950 -2.24630  C 4.78680 -4.67220 -3.42600  O 3.18370 -1.27000 0.93760  O 0.88400 2.83050 -0.71540  H 5.23320 2.30380 -1.15930  H 4.14710 -4.67180 -1.35700  H 3.00260 -3.90040 -2.46560  H 4.70510 -4.07600 -4.34240  H 4.34880 -5.65560 -3.64060  H 5.85440 -4.83320 -3.22960  H 4.69170 -1.76980 -3.87820  H 3.21920 -1.46700 -2.95840  H 5.49030 0.28460 -3.22880  H 5.62970 -0.32800 -1.57540  H 3.42620 1.38690 -2.80250  H 2.94180 0.23220 -1.55400  O 4.78250 1.02280 0.28630  H 4.07600 0.39110 0.63190  H 5.83160 -2.74500 -1.82000  H -0.71250 1.84220 -0.76660  C 3.63020 3.56960 0.79520  H 4.53730 4.15790 0.61020  H 2.84590 4.27370 1.11240  H 3.85470 2.90180 1.63870  H 3.12720 3.50380 -1.30590  H 2.51890 -4.14080 0.26380  C -2.20370 1.22620 -2.90550  C -3.28040 1.59360 -1.85520  C -2.76260 1.15010 -0.46920  H -1.65660 2.12680 -3.23480  H -2.68050 0.80960 -3.81340  H -3.56370 2.67040 -1.88550  H -2.94880 1.93660 0.29270  H -3.32070 0.25690 -0.12020  O -4.51260 0.93940 -2.17340  H -4.44810 -0.04860 -2.01850  C -10.24040 -3.17730 0.87830  C -8.89110 -2.83810 1.51810  C -8.16760 -1.83150 0.59850  C -6.76190 -1.51360 1.10700  C -5.97800 -0.70870 0.08650  O -5.03270 -1.18490 -0.54180  O -11.19010 -2.46890 0.65540  O -10.24860 -4.50010 0.51620  N -9.20870 -2.36400 2.88340  N -6.33510 0.61040 -0.10610  H -8.28270 -3.78370 1.60640  H -8.10440 -2.25420 -0.42890  H -8.76960 -0.90520 0.49870  H -6.79300 -0.98090 2.07740  H -6.19900 -2.45600 1.30010  H -11.11290 -4.78850 0.11410  H -8.36700 -2.25390 3.43920  H -9.70100 -1.47370 2.86840  H -5.82580 1.17510 -0.80900  H -7.10950 1.05080 0.35420  C 6.15060 -1.46180 2.28180  C 6.35010 -1.47300 3.78930  C 7.12130 -0.45120 1.61740  O 7.03860 -0.59320 0.20230  O 5.56040 -1.25090 4.67240  O 7.66360 -1.75660 4.10460  N 6.31130 -2.85540 1.79790  H 5.08040 -1.15110 2.07640  H 6.88580 0.58780 1.90830  H 8.17900 -0.68290 1.84760  H 6.15790 -0.20740 -0.12630  H 7.82030 -1.80550 5.08370  H 6.05090 -2.88610 0.80750  H 7.29000 -3.13630 1.85560  C -1.36150 4.47340 0.36770  C -2.84250 4.82980 0.31120  C -0.48190 5.74150 0.27540  O 0.89540 5.38260 0.31870  O -3.73700 4.26460 -0.27260  O -3.10820 5.96880 1.03190  N -1.19210 3.68050 1.60830  H -1.13290 3.79900 -0.51600  H -0.68900 6.31020 -0.64880  H -0.61030 6.40740 1.15180  H 1.10300 4.72330 -0.40380  H -4.07400 6.20630 1.04040  H -0.39460 3.05010 1.50450  H -0.99900 4.28690 2.40340 |

| **Structure 24** (Figure S7) | **Structure 25** (Figure S8) |
| --- | --- |
| PM6 structure | PM6 structure |
| E(PM6) = -1.02283038 a.u.  E^SP^(B3LYP/6-311+G(d,p)) = -2641.47865484 a.u.  E^SP^(O3LYP/6-311+G(d,p)) = -2640.45639050 a.u.  E^SP^(M062X/6-311+G(d,p)) = -2640.43249355 a.u. | E(PM6) = -1.08081126 a.u. |
| C -1.29940 1.02240 -0.83020  C -2.13100 1.50950 0.09480  H -3.20170 1.60950 -0.16820  C 0.49360 -0.72740 -1.33290  C 0.76610 -1.72100 -0.22640  C 1.59930 -1.38150 0.76730  C 2.11810 0.01910 0.72570  C 0.88880 0.96540 0.53060  C 0.17160 0.72480 -0.81380  H 0.30020 -2.70460 -0.31310  H 1.89440 -2.03330 1.57870  H 2.81810 0.11400 -0.15410  H 0.61950 1.43630 -1.56950  C -0.41350 -1.32880 -2.37490  C -1.63900 -1.85220 -2.29670  H 0.09440 -1.40400 -3.35180  H -1.74820 0.79370 -1.81630  O -3.09390 -2.84170 -0.52610  C -1.88380 2.00420 1.46170  C -2.54200 1.32200 2.66010  C -3.06270 -0.12490 2.49450  C -1.97780 -1.12850 2.07990  C -2.51420 -2.56640 2.13880  C -1.66980 -3.55130 1.31920  C -2.33610 -3.96960 0.00180  C -2.65820 -1.75680 -1.23700  C -1.37520 -4.56610 -1.02580  C -0.98130 -5.99140 -0.65660  O -3.34210 -0.76240 -1.06090  O -1.23870 3.02300 1.65840  H -3.54710 -0.43530 3.45560  H -1.85660 -4.55840 -2.02620  H -0.46400 -3.93310 -1.12260  H -0.45640 -6.03840 0.30470  H -0.31130 -6.42110 -1.41270  H -1.85400 -6.65360 -0.59000  H -1.48660 -4.46930 1.91500  H -0.66170 -3.12930 1.13230  H -2.56760 -2.90370 3.19120  H -3.57220 -2.57830 1.76970  H -1.08960 -1.02630 2.73070  H -1.62000 -0.89710 1.05540  O -4.17400 -0.17070 1.59060  H -3.87210 -0.13570 0.63560  H -3.20310 -4.65390 0.19630  H 0.16970 0.74520 1.35670  C -3.70150 2.22470 3.10200  H -4.07460 1.93910 4.09230  H -3.40070 3.27890 3.15650  H -4.54910 2.14490 2.40450  H -1.77130 1.30820 3.47740  H -2.07530 -2.33900 -3.18710  C 2.83480 0.61560 1.94150  C 2.69870 2.14430 1.71340  C 1.49400 2.35940 0.76530  H 2.36990 0.31290 2.89220  H 3.89150 0.31030 1.99310  H 2.62740 2.71900 2.65690  H 0.75690 3.05720 1.20420  H 1.82060 2.83630 -0.17840  O 3.89510 2.66110 1.11790  H 3.99810 2.30380 0.18460  H 1.49720 -0.59290 -1.85050  C 8.39760 -3.70540 -0.04590  C 8.34530 -2.38030 -0.81210  C 7.39070 -1.43130 -0.05710  C 7.18400 -0.11990 -0.81410  C 6.08740 0.71280 -0.17570  O 4.98030 0.85430 -0.68830  O 8.77780 -3.93660 1.07460  O 7.88140 -4.70320 -0.83310  N 9.75180 -1.93650 -0.92640  N 6.36560 1.31960 1.03680  H 7.95140 -2.57640 -1.85050  H 6.40960 -1.93560 0.08600  H 7.77410 -1.23860 0.96600  H 8.12370 0.46180 -0.88210  H 6.88340 -0.32430 -1.86800  H 7.91960 -5.59940 -0.40060  H 9.84010 -1.15270 -1.56470  H 10.13600 -1.66150 -0.02540  H 5.65040 1.92400 1.47340  H 7.26690 1.29490 1.47570  C -6.40130 -1.71850 -0.76140  C -7.52780 -1.14920 -1.61130  C -6.82100 -1.76380 0.73000  O -5.81290 -2.43690 1.47730  O -7.51960 -0.22020 -2.37880  O -8.70300 -1.83760 -1.38630  N -6.05170 -3.04300 -1.33050  H -5.50020 -1.04270 -0.88420  H -6.98630 -0.75120 1.13670  H -7.72550 -2.38460 0.88320  H -5.00220 -1.83670 1.58070  H -9.45080 -1.51420 -1.95350  H -5.17460 -3.35980 -0.90760  H -6.76490 -3.73010 -1.08840  C 0.07190 5.49200 -0.62160  C 0.87280 6.29050 -1.64190  C -1.03150 6.37760 0.01400  O -2.02960 5.56100 0.61590  O 2.06030 6.48730 -1.69440  O 0.03890 6.85170 -2.58380  N -0.44800 4.28570 -1.31220  H 0.79120 5.14820 0.17660  H -0.61280 7.07470 0.76000  H -1.60040 6.94410 -0.75270  H -1.63750 4.96900 1.31020  H 0.53420 7.35080 -3.28680  H -0.89600 3.67570 -0.61780  H -1.17530 4.55100 -1.97840 | C -0.83990 1.43020 2.60440  C -0.22550 2.39600 1.90540  H 0.62710 2.92770 2.35920  C -0.23730 -2.56980 0.91310  C -0.19640 -1.93500 -0.26770  C -1.30560 -1.17450 -0.83500  C -2.59930 -1.46220 -0.64170  C -3.04020 0.87720 1.53910  C -1.93860 0.54450 2.22530  H -1.15170 -2.60630 1.51650  H 0.72570 -1.92660 -0.87410  H -0.99840 -0.30360 -1.43560  H -2.92860 -2.34300 -0.07760  H -1.81280 -0.47830 2.60640  C 0.90380 -3.27600 1.49600  C 2.10120 -2.74180 1.76700  H 0.71160 -4.33450 1.72290  H -0.45820 1.21030 3.61740  O 3.62100 -1.01660 0.90790  C -0.51180 2.85130 0.54000  C 0.64740 3.31590 -0.33870  C 1.92300 2.44220 -0.30910  C 1.70970 1.04280 -0.89550  C 3.00100 0.47960 -1.50460  C 3.08130 -1.05300 -1.46870  C 4.02750 -1.57620 -0.38570  C 2.46670 -1.32550 1.60140  C 4.15380 -3.09910 -0.34730  C 5.04340 -3.59530 -1.48300  O 1.94520 -0.36730 2.13730  O -1.64820 2.95950 0.09400  H 2.73820 2.99520 -0.83730  H 4.59560 -3.39860 0.62570  H 3.16390 -3.59020 -0.40290  H 4.55010 -3.51760 -2.45740  H 5.32720 -4.64280 -1.33770  H 5.97970 -3.01000 -1.55130  H 3.44360 -1.42890 -2.44920  H 2.06650 -1.49730 -1.35130  H 3.09070 0.82880 -2.55220  H 3.88750 0.90770 -0.97240  H 0.90380 1.05890 -1.66590  H 1.32400 0.37540 -0.09590  O 2.43700 2.35070 1.03500  H 2.11540 1.51340 1.48790  H 5.04440 -1.09560 -0.45870  H -3.18310 1.88130 1.11670  C 0.98080 4.75650 0.05880  H 1.64020 5.22770 -0.68020  H 0.08210 5.37980 0.13440  H 1.50300 4.78910 1.02610  H 0.27230 3.30520 -1.40330  H 2.90710 -3.34130 2.20560  C -3.69700 -0.59400 -1.16970  C -4.69930 -0.15160 -0.09370  C -4.17110 -0.08260 1.34500  H -3.28400 0.31110 -1.67460  H -4.24220 -1.13850 -1.97650  H -5.18130 0.80800 -0.39620  H -5.00780 0.20030 2.02220  H -3.88970 -1.11360 1.67710  O -5.77830 -1.12840 -0.00650  H -5.98750 -1.50060 -0.94870  O -6.89260 -1.41080 -2.34790  H -7.05620 -0.44950 -2.38280  H -7.73710 -1.77410 -2.02160  O 6.87160 -1.04480 -1.65140  H 6.74620 -0.36680 -2.32060  H 7.15260 -0.55230 -0.86210  O 6.36140 1.21140 -0.89160  H 6.66680 1.70100 -0.11520  H 5.38450 1.14020 -0.74830  O 4.89670 1.55760 1.01060  H 4.23590 2.27500 1.12090  H 4.45200 0.75250 1.32730  O -4.43810 -3.23470 1.29350  H -5.03570 -2.58050 0.87270  H -4.94970 -4.01440 1.45470  O -3.45610 2.53730 -1.39660  H -3.12380 3.44670 -1.40360  H -3.36410 2.25960 -0.45550  O -1.05180 1.59100 -2.45460  H -1.75820 1.94910 -1.86320  H -1.43930 1.53690 -3.32060  O -8.11390 -0.31480 -0.62600  H -7.40230 -0.41980 0.05120  H -8.84350 0.12630 -0.21380 |

| **Structure 26-TS** (Figure S8) | **Structure 27** (Figure S8) |
| --- | --- |
| PM6 structure | PM6 structure |
| E(PM6) = -1.02696498 a.u. | E(PM6) = -1.10679595 a.u. |
| C 0.66360 0.67540 -2.17580  C -0.06390 1.69400 -1.64070  H -1.04940 1.86740 -2.11890  C 0.65680 -1.90250 -0.86420  C 0.39800 -1.22920 0.31060  C 1.42290 -0.60170 1.03870  C 2.74400 -0.63710 0.53190  C 2.80230 0.51590 -0.82220  C 1.93110 0.10030 -1.86240  H 1.69670 -2.17100 -1.13180  H -0.63710 -1.12770 0.67520  H 1.17330 -0.01470 1.93180  H 2.99890 -1.58130 -0.02270  H 2.31380 -0.63730 -2.57340  C -0.34850 -2.61510 -1.64150  C -1.62960 -2.32260 -1.89930  H 0.03740 -3.57300 -2.03820  H 0.16910 0.17070 -3.03360  O -3.47200 -1.02410 -0.87000  C 0.19350 2.63110 -0.56560  C -0.98890 3.39860 0.04500  C -2.26640 2.56330 0.31000  C -1.94410 1.22870 0.98970  C -3.18710 0.49330 1.50310  C -2.99910 -1.03240 1.51880  C -3.73340 -1.74500 0.38140  C -2.35920 -1.06610 -1.66480  C -3.43330 -3.23870 0.27610  C -4.13870 -4.00800 1.38890  O -2.17470 -0.03810 -2.29340  O 1.30550 2.91410 -0.11940  H -2.98940 3.17190 0.90240  H -3.78450 -3.61050 -0.70850  H -2.34500 -3.43670 0.31200  H -3.67380 -3.83870 2.36570  H -4.12560 -5.08610 1.19800  H -5.19940 -3.70640 1.48220  H -3.37690 -1.44620 2.47780  H -1.91540 -1.28880 1.50560  H -3.43300 0.84870 2.52220  H -4.07220 0.75710 0.87210  H -1.22930 1.39490 1.83070  H -1.39580 0.58530 0.26930  O -2.99300 2.35490 -0.91140  H -2.53070 1.68000 -1.49890  H -4.84550 -1.56070 0.43550  H 2.51120 1.46700 -0.31120  C -1.30260 4.59750 -0.85070  H -1.98180 5.29760 -0.35020  H -0.39650 5.15100 -1.12280  H -1.80220 4.27930 -1.77780  H -0.63830 3.77730 1.04320  H -2.25230 -3.04050 -2.45500  C 3.94210 -0.15870 1.33070  C 5.01420 0.19640 0.28230  C 4.31260 0.47840 -1.05970  H 3.70420 0.71660 1.96940  H 4.29410 -0.94390 2.02620  H 5.68060 1.01600 0.61520  H 4.64480 1.43400 -1.50370  H 4.58680 -0.31050 -1.79500  O 5.85590 -0.95240 0.01270  H 6.20800 -1.36280 0.89370  O 7.50840 -1.72980 1.85410  H 7.95180 -0.86210 1.90020  H 8.04960 -2.22690 1.21120  O -6.65950 -2.11480 1.54280  H -6.73600 -1.39180 2.17330  H -6.91710 -1.71980 0.69460  O -6.54780 0.19180 0.76970  H -6.97830 0.65120 0.03470  H -5.60270 0.47450 0.69450  O -5.26170 1.28670 -0.97170  H -4.83770 2.16620 -0.94580  H -4.57590 0.68860 -1.32090  O 4.06910 -2.71460 -1.40000  H 4.73500 -2.12040 -0.98770  H 4.43960 -3.58810 -1.38370  O 2.72580 2.84200 1.80830  H 2.34920 3.66750 1.44670  H 2.74700 2.24390 1.02180  O 0.55960 1.68940 2.95810  H 1.29720 2.21120 2.54400  H 0.65480 1.79350 3.89540  O 8.38600 -0.70230 -0.10300  H 7.50250 -0.56870 -0.52260  H 9.04600 -0.41410 -0.71840 | C -0.34400 0.17410 2.15340  C -0.16830 1.47880 1.92870  H 0.54230 2.02980 2.57370  C -0.36000 -2.10720 0.98710  C -0.01600 -2.06710 -0.48400  C -0.98490 -1.78600 -1.36800  C -2.31240 -1.44200 -0.77240  C -2.05110 -0.33550 0.30210  C -1.18810 -0.85730 1.46730  H -1.12560 -2.94880 1.08090  H 1.00830 -2.30440 -0.77110  H -0.86070 -1.76530 -2.44240  H -2.72570 -2.36450 -0.27280  H -1.89450 -1.25470 2.25680  C 0.81380 -2.55220 1.81740  C 2.03920 -2.05970 2.01290  H 0.60490 -3.52910 2.29060  H 0.22330 -0.26110 2.99770  O 3.67080 -0.59830 0.83160  C -0.79370 2.39080 0.95350  C 0.02160 3.00850 -0.17800  C 1.44170 2.45930 -0.44570  C 1.44560 0.99950 -0.91570  C 2.79370 0.60260 -1.53420  C 3.04730 -0.91070 -1.51150  C 3.97870 -1.35780 -0.38330  C 2.62680 -0.74760 1.69750  C 4.00520 -2.86430 -0.13590  C 4.79340 -3.57010 -1.23530  O 2.38540 0.27430 2.32300  O -1.96140 2.74000 1.07860  H 1.94410 3.12190 -1.19420  H 4.48250 -3.06030 0.84660  H 2.98440 -3.28570 -0.06970  H 4.24540 -3.59630 -2.18280  H 5.02670 -4.60350 -0.95830  H 5.75510 -3.06100 -1.43540  H 3.51300 -1.22440 -2.47080  H 2.08500 -1.46630 -1.47050  H 2.84000 0.96950 -2.57800  H 3.62440 1.12610 -0.99650  H 0.63120 0.83540 -1.65230  H 1.19300 0.33240 -0.06780  O 2.27510 2.64590 0.70830  H 2.12600 1.93040 1.39130  H 5.02470 -0.95970 -0.54300  H -1.50800 0.49200 -0.21940  C 0.10600 4.51690 0.08200  H 0.42860 5.05880 -0.81420  H -0.85870 4.93590 0.39180  H 0.83830 4.73450 0.87450  H -0.58120 2.83200 -1.12420  H 2.75850 -2.65140 2.60650  C -3.42510 -0.83300 -1.63200  C -4.33180 -0.14850 -0.58600  C -3.46830 0.14760 0.66150  H -3.01750 -0.08450 -2.34370  H -3.96170 -1.58400 -2.23060  H -4.86170 0.73690 -0.98620  H -3.47830 1.22150 0.93800  H -3.87380 -0.39530 1.54030  O -5.34560 -1.07030 -0.10990  H -5.78770 -1.56360 -0.89880  O -7.19340 -1.81370 -1.77780  H -7.46280 -0.88980 -1.93990  H -7.78760 -2.10080 -1.06030  O 6.79430 -1.13070 -1.73670  H 6.65190 -0.45580 -2.40840  H 7.02980 -0.62730 -0.94060  O 6.17600 1.12880 -1.07590  H 6.53480 1.77150 -0.44860  H 5.20140 1.16310 -0.90770  O 4.76850 2.09510 0.65970  H 4.10490 2.80920 0.57800  H 4.33290 1.40000 1.18330  O -3.79450 -2.83680 1.64030  H -4.30440 -2.23440 1.05530  H -4.40700 -3.48970 1.95370  O -3.71580 3.19120 -0.54090  H -3.03370 3.81640 -0.22450  H -3.79360 2.55420 0.20450  O -2.06050 1.99540 -2.29100  H -2.79120 2.31260 -1.69410  H -2.19370 2.43440 -3.12240  O -7.78140 -0.32500 -0.01540  H -6.86490 -0.31200 0.35570  H -8.31590 0.25180 0.51180 |

| **Structure I** (Figure S9) | **Structure II** (Figure S9) |
| --- | --- |
| PM6 structure | PM6 structure |
| E(PM6) = -0.29418192 a.u. | E(PM6) = -0.53550312 a.u. |
| C -1.91310 1.20740 2.17100  C -1.47190 2.17240 1.34810  H -0.89990 3.01170 1.77300  C 0.15310 -2.47860 0.55130  C 0.09720 -1.80100 -0.60370  C -1.14880 -1.28960 -1.17170  C -2.31120 -1.95350 -1.20160  C -3.69670 -0.07690 1.01330  C -2.65000 -0.01200 1.84720  H -0.75430 -2.73490 1.10890  H 1.01160 -1.54260 -1.16110  H -1.08850 -0.25620 -1.53810  H -2.41470 -2.98360 -0.85900  H -2.28350 -0.89910 2.37210  C 1.39440 -2.92070 1.18980  C 2.38670 -2.12170 1.59840  H 1.46260 -4.00650 1.34260  H -1.67890 1.29380 3.24470  O 3.56360 -0.05250 1.06380  C -1.60180 2.23960 -0.11570  C -0.67950 3.22180 -0.84870  C 0.83040 2.93170 -0.65450  C 1.21600 1.52360 -1.13390  C 2.73740 1.39910 -1.27680  C 3.21990 -0.05800 -1.34520  C 4.19540 -0.37610 -0.20540  C 2.38910 -0.64710 1.51620  C 4.73890 -1.80820 -0.23140  C 5.86440 -1.95630 -1.24990  O 1.54990 0.11970 1.92550  O -2.40190 1.60080 -0.77850  H 1.41510 3.72480 -1.18070  H 5.11470 -2.06660 0.78060  H 3.93390 -2.53570 -0.45170  H 5.52690 -1.73660 -2.26950  H 6.25870 -2.98020 -1.25230  H 6.70590 -1.28780 -1.02910  H 3.71810 -0.25010 -2.31490  H 2.35750 -0.76280 -1.32090  H 3.07840 1.95210 -2.17130  H 3.21240 1.91130 -0.40640  H 0.71660 1.28270 -2.08960  H 0.84100 0.78340 -0.39270  O 1.21640 3.12450 0.70950  H 1.20680 2.26930 1.21640  H 5.03230 0.36700 -0.16470  H -4.05790 0.80700 0.47640  C -1.00610 4.65100 -0.41390  H -0.56580 5.38400 -1.09940  H -2.08600 4.83330 -0.38490  H -0.59640 4.86010 0.58660  H -0.90300 3.12300 -1.94370  H 3.27870 -2.52380 2.08960  C -3.57380 -1.30820 -1.68340  C -4.75820 -1.55630 -0.73660  C -4.44460 -1.34570 0.75410  H -3.42760 -0.20140 -1.77830  H -3.81510 -1.66140 -2.70510  H -5.63380 -0.94520 -1.05500  H -5.39570 -1.34830 1.33000  H -3.89310 -2.23770 1.12530  O -5.18010 -2.94010 -0.78140  H -5.16370 -3.28410 -1.69050 | C -3.12300 -2.61540 1.88690  C -3.84060 -2.69280 0.75420  H -4.93820 -2.73240 0.82550  C -0.91040 1.19100 1.50340  C -1.01490 0.92010 0.19480  C -0.48660 -0.29840 -0.41620  C 0.71700 -0.82700 -0.16030  C -0.77200 -3.22400 1.36630  C -1.67560 -2.51720 2.05890  H -0.37760 0.51870 2.18380  H -1.55830 1.59300 -0.48810  H -1.18900 -0.80920 -1.08980  H 1.44030 -0.34430 0.49530  H -1.37240 -1.81290 2.83840  C -1.49370 2.36470 2.15540  C -2.79100 2.69120 2.16740  H -0.76560 2.99370 2.68570  H -3.66980 -2.58810 2.84380  O -4.82790 2.60540 0.82400  C -3.34880 -2.67150 -0.63210  C -4.37930 -2.37530 -1.72870  C -5.03770 -0.97830 -1.60020  C -3.99570 0.15150 -1.59040  C -4.66540 1.50840 -1.83600  C -3.80570 2.69810 -1.38160  C -4.50240 3.49970 -0.27560  C -3.88290 1.90200 1.56220  C -3.71990 4.73180 0.18970  C -3.89600 5.89810 -0.77730  O -4.14160 0.73580 1.74610  O -2.19830 -2.89150 -0.97160  H -5.77820 -0.85600 -2.42720  H -4.08040 5.02550 1.19760  H -2.64380 4.49920 0.30650  H -3.52710 5.65830 -1.78140  H -3.34490 6.78130 -0.43120  H -4.94840 6.19310 -0.87400  H -3.59730 3.36700 -2.23890  H -2.80450 2.35350 -1.03540  H -4.91970 1.61360 -2.90680  H -5.63960 1.52080 -1.29210  H -3.20790 -0.03360 -2.34290  H -3.48490 0.14960 -0.60230  O -5.86770 -0.90920 -0.43740  H -5.37600 -0.53340 0.34110  H -5.54640 3.77940 -0.57020  H -1.07430 -3.92170 0.57620  C -5.44730 -3.46870 -1.74630  H -6.03010 -3.43810 -2.67400  H -5.00970 -4.46960 -1.66390  H -6.15870 -3.33580 -0.91620  H -3.82900 -2.39920 -2.70630  H -3.15090 3.57820 2.69920  C 1.15180 -2.13140 -0.75030  C 1.50630 -3.19340 0.30510  C 0.70010 -3.12180 1.61070  H 0.35030 -2.54650 -1.40610  H 2.03380 -1.96630 -1.41210  H 1.43430 -4.20930 -0.14980  H 1.02870 -3.94860 2.27980  H 0.96790 -2.19500 2.15800  O 2.87650 -3.03240 0.74250  H 3.48890 -3.03910 -0.05290  C 9.33340 1.14210 -0.04900  C 8.02980 1.25070 -0.84560  C 6.98350 0.33790 -0.17090  C 5.65960 0.33750 -0.93380  C 4.72340 -0.74060 -0.42080  O 4.41690 -1.72300 -1.09110  O 9.56390 1.43890 1.09690  O 10.30320 0.58570 -0.84180  N 7.70970 2.69480 -0.88220  N 4.17100 -0.57200 0.83770  H 8.21830 0.90360 -1.90210  H 7.38420 -0.69900 -0.11930  H 6.83160 0.64870 0.88280  H 5.16470 1.32720 -0.88820  H 5.83780 0.14100 -2.01700  H 11.18510 0.51030 -0.38460  H 6.95000 2.88650 -1.52680  H 7.44780 3.04630 0.03570  H 3.55010 -1.31320 1.21010  H 4.44770 0.15370 1.47120 |

| **Structure III** (Figure S9) | **Structure IV** (Figure S9) |
| --- | --- |
| PM6 structure | PM6 structure |
| E(PM6) = -0.52390221 a.u. | E(PM6) = -0.76204832 a.u. |
| C -2.79920 -1.76610 1.85580  C -3.64810 -2.00460 0.83790  H -4.71080 -1.75480 1.02250  C -1.04060 0.77640 1.40060  C -1.13440 0.61330 0.07050  C -0.48520 -0.48510 -0.64280  C 0.67900 -1.01390 -0.23690  C -0.59610 -2.75590 1.24590  C -1.36510 -1.96670 2.01650  H -0.42920 0.12100 2.02940  H -1.73860 1.29600 -0.54780  H -1.04680 -0.90430 -1.48290  H 1.24890 -0.56540 0.58170  H -0.94380 -1.48240 2.90310  C -1.61810 1.91210 2.12300  C -2.88770 2.33140 2.14100  H -0.87060 2.46690 2.71240  H -3.25760 -1.32510 2.76350  O -4.89670 2.41230 0.72380  C -3.41560 -2.48440 -0.52730  C -4.59410 -2.38760 -1.50770  C -5.19950 -0.96480 -1.63120  C -4.10250 0.10850 -1.73680  C -4.69750 1.49870 -1.98860  C -3.81350 2.63220 -1.44290  C -4.49560 3.37670 -0.29000  C -4.05070 1.66910 1.52230  C -3.67000 4.52990 0.28680  C -3.76690 5.77330 -0.59160  O -4.45490 0.55300 1.76970  O -2.36990 -2.94700 -0.95510  H -5.89150 -0.94010 -2.50650  H -4.04240 4.76490 1.30570  H -2.60890 4.23780 0.40830  H -3.38160 5.59330 -1.60210  H -3.18630 6.60090 -0.16540  H -4.80180 6.12410 -0.69070  H -3.58000 3.35380 -2.24970  H -2.82530 2.23660 -1.11380  H -4.87560 1.64300 -3.06960  H -5.70460 1.54340 -1.50970  H -3.38280 -0.15620 -2.53250  H -3.52360 0.11380 -0.78850  O -6.08640 -0.67930 -0.54990  H -5.59460 -0.35760 0.25790  H -5.51940 3.72690 -0.58320  H -1.01280 -3.26340 0.35860  C -5.66860 -3.40040 -1.11560  H -6.39540 -3.53700 -1.92480  H -5.24050 -4.38120 -0.88240  H -6.23620 -3.05320 -0.23810  H -4.19240 -2.66710 -2.51760  H -3.17770 3.21160 2.72800  C 1.32030 -2.22170 -0.83710  C 1.65110 -3.26200 0.25260  C 0.82860 -3.09910 1.54400  H 0.66650 -2.68560 -1.60600  H 2.25460 -1.93510 -1.37520  H 1.55890 -4.29210 -0.15870  H 0.86330 -4.04660 2.12620  H 1.32160 -2.35370 2.20120  O 3.01760 -3.10660 0.69660  H 3.63940 -3.12750 -0.09090  C 9.34950 1.24110 0.03330  C 8.07570 1.29540 -0.81560  C 7.02760 0.37240 -0.15780  C 5.73370 0.31990 -0.96880  C 4.80620 -0.77010 -0.46640  O 4.54110 -1.77010 -1.12900  O 9.52560 1.56850 1.18050  O 10.36490 0.69630 -0.70850  N 7.72100 2.72920 -0.90160  N 4.20820 -0.59330 0.77000  H 8.31420 0.92720 -1.85460  H 7.45190 -0.65230 -0.06590  H 6.82880 0.70430 0.88160  H 5.21240 1.29710 -0.96440  H 5.95750 0.10460 -2.04000  H 11.23010 0.65670 -0.21620  H 6.98470 2.88570 -1.58180  H 7.41260 3.09610 -0.00450  H 3.60280 -1.34850 1.13860  H 4.45120 0.14680 1.40100 | C -0.62390 0.83650 -1.56150  C -1.67840 1.65050 -1.35640  H -2.63780 1.31550 -1.79570  C 0.16250 -0.78410 0.98780  C -0.29720 0.19130 1.78840  C 0.34430 1.49990 1.89890  C 1.67070 1.66030 1.78200  C 1.42170 2.03810 -0.79760  C 0.77860 0.92130 -1.18500  H 1.08880 -0.68430 0.41070  H -1.21130 0.05500 2.38770  H -0.33230 2.35240 2.00650  H 2.34220 0.79830 1.71320  H 1.33710 -0.00980 -1.33190  C -0.43350 -2.12080 0.92090  C -1.69520 -2.45340 0.62630  H 0.28580 -2.92250 1.15490  H -0.84460 -0.09240 -2.12650  O -3.96770 -1.50600 0.84190  C -1.80970 2.89330 -0.59490  C -3.24110 3.39910 -0.33970  C -4.14710 2.37280 0.38230  C -3.41830 1.70560 1.56010  C -4.36550 0.87870 2.43730  C -3.71120 -0.39720 2.99090  C -4.20790 -1.65520 2.27310  C -2.76260 -1.54870 0.17430  C -3.64660 -2.96340 2.83050  C -4.39080 -3.38950 4.09200  O -2.77040 -0.89790 -0.85330  O -0.89330 3.55780 -0.14040  H -5.09430 2.87000 0.69960  H -3.74090 -3.75670 2.05910  H -2.56370 -2.87400 3.04570  H -4.29390 -2.65110 4.89650  H -3.99920 -4.34010 4.47590  H -5.46250 -3.53560 3.90570  H -3.93290 -0.49450 4.07280  H -2.60060 -0.32780 2.92920  H -4.74420 1.49950 3.27050  H -5.27200 0.60400 1.84050  H -2.90130 2.46940 2.17130  H -2.61580 1.05660 1.14860  O -4.63620 1.36970 -0.51930  H -3.89320 0.75970 -0.82000  H -5.33170 -1.68130 2.23090  H 0.88060 2.98770 -0.65210  C -3.86930 3.85730 -1.65390  H -4.78120 4.43940 -1.47440  H -3.18430 4.48610 -2.23450  H -4.15890 3.00070 -2.28040  H -3.14590 4.29080 0.33680  H -2.00980 -3.50540 0.61320  C 2.36090 2.98490 1.74320  C 3.30300 3.07490 0.52560  C 2.90390 2.12860 -0.62500  H 1.63010 3.82080 1.70830  H 2.94850 3.14500 2.67290  H 3.40070 4.12840 0.17990  H 3.35570 2.48340 -1.57550  H 3.36710 1.12890 -0.45130  O 4.64500 2.76360 0.93510  H 4.70500 1.81190 1.24570  C 10.58300 -1.99790 -0.13040  C 9.22160 -2.04290 -0.83090  C 8.44230 -0.77010 -0.43620  C 7.02750 -0.77320 -1.01460  C 6.19690 0.35630 -0.43610  O 5.27750 0.16420 0.35700  O 11.48690 -1.20930 -0.25130  O 10.66470 -3.03280 0.76480  N 9.52330 -2.20440 -2.27010  N 6.47080 1.64330 -0.86480  H 8.66340 -2.95900 -0.48260  H 8.39070 -0.70340 0.67350  H 8.99930 0.13260 -0.76030  H 7.04240 -0.71610 -2.12070  H 6.51340 -1.73200 -0.77090  H 11.54160 -3.07260 1.23590  H 8.68270 -2.39900 -2.80350  H 9.96580 -1.37500 -2.65910  H 5.94850 2.43040 -0.44640  H 7.25860 1.87860 -1.43990  C -5.92370 -1.58600 -1.74820  C -6.32830 -1.82290 -3.19430  C -6.87230 -0.55230 -1.08680  O -6.55580 -0.46140 0.29990  O -5.69750 -1.66050 -4.20840  O -7.63740 -2.25540 -3.26850  N -5.90100 -2.90240 -1.06280  H -4.86460 -1.18290 -1.74350  H -6.77920 0.44240 -1.55930  H -7.92600 -0.88860 -1.10180  H -5.67210 0.03090 0.40850  H -7.92320 -2.45230 -4.19850  H -5.52260 -2.76710 -0.12050  H -6.85060 -3.25740 -0.95310 |

| **Structure V** (Figure S9) | **Structure VI** (Figure S9) |
| --- | --- |
| PM6 structure | PM6 structure |
| E(PM6) = -0.74182644 a.u. | E(PM6) = -0.97374576 a.u. |
| C -0.56740 0.64040 -1.40650  C -1.62870 1.47210 -1.34120  H -2.57440 1.06540 -1.75320  C 0.14580 -0.70920 1.08550  C -0.38100 0.34400 1.74300  C 0.29340 1.62580 1.86630  C 1.59700 1.76210 1.53550  C 1.43230 1.92480 -0.64220  C 0.83180 0.77900 -1.04770  H 1.16230 -0.69500 0.67630  H -1.38220 0.28080 2.19960  H -0.32180 2.48110 2.14720  H 2.22160 0.86640 1.39030  H 1.43760 -0.11090 -1.24460  C -0.47490 -2.03420 1.05740  C -1.74260 -2.37980 0.80240  H 0.23870 -2.83740 1.31060  H -0.78430 -0.35890 -1.83950  O -4.01990 -1.42570 0.98380  C -1.79510 2.81740 -0.79390  C -3.23760 3.34400 -0.67230  C -4.18540 2.42050 0.13070  C -3.51340 1.89440 1.40990  C -4.47980 1.09390 2.29070  C -3.80180 -0.08960 3.00020  C -4.24420 -1.43810 2.42490  C -2.82570 -1.52120 0.30410  C -3.61680 -2.65260 3.10920  C -4.30860 -2.95900 4.43350  O -2.85700 -0.97210 -0.78140  O -0.90230 3.56190 -0.42270  H -5.13600 2.96190 0.35170  H -3.70000 -3.53170 2.43570  H -2.53260 -2.49990 3.27770  H -4.21530 -2.13250 5.14780  H -3.87150 -3.84720 4.90740  H -5.37900 -3.16070 4.29960  H -4.04150 -0.06850 4.08220  H -2.69270 0.00520 2.94870  H -4.95230 1.76070 3.03550  H -5.32400 0.71710 1.65790  H -3.08030 2.73620 1.98230  H -2.65150 1.25550 1.11960  O -4.65820 1.32570 -0.66540  H -3.91070 0.68560 -0.88330  H -5.36450 -1.51950 2.40100  H 0.83780 2.85250 -0.52170  C -3.79720 3.62810 -2.06450  H -4.72470 4.21030 -2.00650  H -3.08800 4.19500 -2.67890  H -4.04120 2.69740 -2.59730  H -3.17130 4.31600 -0.11310  H -2.05090 -3.43380 0.85290  C 2.37990 3.03680 1.62160  C 3.36270 3.10800 0.43410  C 2.91710 2.15080 -0.69100  H 1.71900 3.92730 1.62400  H 2.94140 3.08240 2.57890  H 3.49760 4.15190 0.07750  H 3.17740 2.56960 -1.68470  H 3.49350 1.20020 -0.62030  O 4.68320 2.76010 0.87150  H 4.71720 1.80160 1.16680  C 10.60610 -2.04700 -0.04380  C 9.26550 -2.07510 -0.78440  C 8.48910 -0.79470 -0.40910  C 7.09240 -0.78000 -1.02970  C 6.25680 0.35610 -0.47230  O 5.30820 0.17040 0.28790  O 11.52090 -1.26700 -0.13440  O 10.65090 -3.08650 0.84860  N 9.60810 -2.23630 -2.21430  N 6.56120 1.64240 -0.88090  H 8.68720 -2.98580 -0.45520  H 8.40510 -0.73060 0.69870  H 9.06580 0.10250 -0.71350  H 7.14120 -0.71900 -2.13460  H 6.56080 -1.73420 -0.80560  H 11.51340 -3.13760 1.34480  H 8.78210 -2.42270 -2.77280  H 10.06930 -1.41030 -2.58830  H 6.03270 2.43280 -0.47830  H 7.36710 1.87180 -1.43290  C -5.93890 -1.72680 -1.69120  C -6.30050 -2.14040 -3.10860  C -6.89910 -0.61510 -1.19280  O -6.65990 -0.38990 0.19380  O -5.63510 -2.11590 -4.11320  O -7.61340 -2.56360 -3.17250  N -5.94970 -2.94790 -0.84740  H -4.87670 -1.33180 -1.70400  H -6.75800 0.32440 -1.75700  H -7.95780 -0.93430 -1.23420  H -5.77490 0.09960 0.30630  H -7.87160 -2.87360 -4.07920  H -5.59660 -2.69920 0.08170  H -6.90640 -3.27800 -0.72120 | C 0.95190 1.34260 1.79340  C 1.71160 1.80270 0.77260  H 2.79180 1.55390 0.84050  C -0.42290 -1.12320 1.42850  C -0.52170 -0.84700 0.11130  C -1.48710 0.09520 -0.42940  C -2.48020 0.57900 0.35030  C -1.39340 2.14810 1.42400  C -0.44880 1.47350 2.13020  H 0.15200 -1.32240 -0.61890  H -1.33020 0.43740 -1.45040  H -2.69680 0.09550 1.32310  H -0.70800 1.07380 3.11750  C 0.43180 -2.18310 1.96510  C 1.71760 -2.46030 1.71820  H -0.11610 -2.84980 2.65450  H 1.51020 0.75870 2.55720  O 3.36790 -2.14910 -0.10730  C 1.39850 2.57150 -0.41700  C 2.46890 2.65790 -1.51720  C 3.09870 1.30260 -1.92580  C 2.03590 0.20310 -2.08760  C 2.62590 -1.09070 -2.66270  C 1.97500 -2.35760 -2.08480  C 2.90290 -3.09570 -1.11500  C 2.68810 -1.64470 0.97720  C 2.30130 -4.36120 -0.50510  C 2.34490 -5.52430 -1.49080  O 3.10390 -0.55370 1.32370  O 0.35860 3.19400 -0.62210  H 3.69760 1.44190 -2.85810  H 2.87130 -4.62990 0.40960  H 1.25880 -4.18790 -0.17260  H 1.75820 -5.32120 -2.39420  H 1.93680 -6.43680 -1.03760  H 3.37020 -5.75470 -1.80750  H 1.70110 -3.04880 -2.90720  H 1.01140 -2.11000 -1.58320  H 2.53440 -1.08890 -3.76480  H 3.72750 -1.11190 -2.46050  H 1.21000 0.56430 -2.72820  H 1.57830 0.00130 -1.09550  O 4.10710 0.88650 -0.99840  H 3.70320 0.62570 -0.11150  H 3.89860 -3.30710 -1.59140  H -1.14430 2.61840 0.45430  C 3.54580 3.65050 -1.08160  H 4.22190 3.88990 -1.91210  H 3.11690 4.59490 -0.72550  H 4.17260 3.23590 -0.27790  H 1.96000 3.06680 -2.43110  H 2.19670 -3.33170 2.18800  C -3.54140 1.54290 -0.07170  C -3.91080 2.41240 1.14660  C -2.67140 2.65350 2.03230  H -3.23460 2.19540 -0.91670  H -4.43460 1.00000 -0.44110  H -4.41240 3.35770 0.83700  H -2.54540 3.75140 2.20270  H -2.85390 2.23390 3.04260  O -4.92700 1.76270 1.92480  H -4.55950 0.94570 2.37910  H -1.12680 -0.71580 2.16510  C -5.13320 -5.19220 -1.58360  C -5.56810 -4.82350 -0.16240  C -5.50670 -3.28720 -0.02890  C -5.82490 -2.83190 1.39510  C -5.52210 -1.35580 1.57270  O -4.54540 -0.95380 2.20290  O -5.64220 -4.92410 -2.64280  O -3.96260 -5.90660 -1.52750  N -6.90010 -5.44230 0.01470  N -6.39000 -0.44670 0.99750  H -4.85720 -5.30180 0.57100  H -4.49010 -2.93500 -0.31340  H -6.19640 -2.81230 -0.75660  H -6.87630 -3.05030 1.66510  H -5.20460 -3.39570 2.13000  H -3.64770 -6.20650 -2.42330  H -7.20670 -5.38660 0.98020  H -7.60380 -4.99910 -0.57150  H -6.21010 0.56560 1.10840  H -7.21340 -0.70880 0.48840  C 6.33240 -1.07310 0.85330  C 7.40810 -0.58140 1.80800  C 6.68210 -0.67170 -0.60370  O 5.76260 -1.30820 -1.48750  O 7.32270 0.17360 2.74330  O 8.64000 -1.09880 1.46010  N 6.18330 -2.53710 1.04880  H 5.35240 -0.58990 1.15420  H 6.64210 0.42360 -0.74320  H 7.66870 -1.06400 -0.91520  H 4.85480 -0.85720 -1.40860  H 9.36080 -0.82370 2.08480  H 5.40310 -2.86010 0.46850  H 7.01780 -3.02140 0.71770  C -1.86440 5.28220 -0.61840  C -3.32940 5.31930 -1.02300  C -1.00030 6.02500 -1.66670  O 0.36960 5.94490 -1.29090  O -4.08950 4.39670 -1.20250  O -3.75880 6.60920 -1.21400  N -1.76700 5.82330 0.75890  H -1.53720 4.19230 -0.57550  H -1.13800 5.60290 -2.67800  H -1.20690 7.11300 -1.68630  H 0.65270 4.98590 -1.24380  H -4.72420 6.66590 -1.45100  H -0.81030 5.69860 1.09760  H -1.95260 6.82660 0.76250 |

| **Structure VII** (Figure S10) | **Structure VIII** (Figure S10) |
| --- | --- |
| PM6 structure | PM6 structure |
| E(PM6) = -0.95964089 a.u. | E(PM6) = -0.94956272 a.u. |
| C 0.92810 1.32290 1.77030  C 1.67710 1.78430 0.73850  H 2.76200 1.55870 0.81180  C -0.46570 -1.10390 1.41140  C -0.52050 -0.79840 0.09220  C -1.48870 0.12830 -0.44350  C -2.44820 0.65340 0.37480  C -1.43780 2.07040 1.38050  C -0.47170 1.41320 2.10160  H 0.20640 -1.22270 -0.61780  H -1.36540 0.45790 -1.47280  H -2.69040 0.11480 1.31920  H -0.73000 1.01750 3.08950  C 0.40270 -2.13700 1.97380  C 1.68780 -2.42270 1.73150  H -0.13670 -2.78650 2.68700  H 1.50530 0.77040 2.54370  O 3.34290 -2.17700 -0.09860  C 1.35390 2.53240 -0.45870  C 2.42820 2.63590 -1.55480  C 3.07950 1.29020 -1.96100  C 2.02900 0.18140 -2.13930  C 2.64430 -1.12490 -2.65570  C 1.95920 -2.37590 -2.08170  C 2.85810 -3.12640 -1.09420  C 2.66700 -1.63640 0.97070  C 2.21460 -4.36350 -0.46870  C 2.22370 -5.54110 -1.43800  O 3.09640 -0.54410 1.29550  O 0.30240 3.13220 -0.67850  H 3.68670 1.43810 -2.88660  H 2.77300 -4.63730 0.45150  H 1.17720 -4.15220 -0.14220  H 1.64680 -5.33170 -2.34630  H 1.78440 -6.43340 -0.97360  H 3.24190 -5.80930 -1.74740  H 1.68200 -3.06550 -2.90380  H 0.99490 -2.10580 -1.59380  H 2.61010 -1.14810 -3.76060  H 3.73420 -1.14280 -2.39630  H 1.23020 0.52450 -2.82290  H 1.52950 0.00030 -1.16270  O 4.08260 0.88420 -1.02390  H 3.67300 0.62410 -0.13930  H 3.84910 -3.37550 -1.56010  H -1.15310 2.60520 0.44950  C 3.48790 3.64520 -1.11610  H 4.16430 3.89350 -1.94370  H 3.04260 4.58380 -0.76420  H 4.11650 3.24190 -0.30830  H 1.91650 3.03650 -2.47100  H 2.16360 -3.28290 2.22630  C -3.55840 1.56550 -0.05810  C -3.93830 2.42070 1.16800  C -2.67800 2.64090 2.02920  H -3.28400 2.22550 -0.90710  H -4.42880 0.98150 -0.41420  H -4.44690 3.36680 0.87730  H -2.50710 3.73410 2.17780  H -2.84640 2.23930 3.04790  O -4.94120 1.75270 1.94740  H -4.55730 0.94340 2.40040  H -1.24260 -0.76480 2.11190  C -5.02850 -5.19780 -1.60390  C -5.44790 -4.84540 -0.17390  C -5.41980 -3.30860 -0.03430  C -5.72380 -2.86690 1.39710  C -5.45200 -1.38500 1.57660  O -4.47460 -0.96350 2.19320  O -5.56130 -4.93720 -2.65310  O -3.84050 -5.88440 -1.57060  N -6.76210 -5.49570 0.02250  N -6.34930 -0.49370 1.01960  H -4.71410 -5.31020 0.54530  H -4.41690 -2.93180 -0.33490  H -6.13300 -2.84700 -0.74780  H -6.76520 -3.11060 1.68380  H -5.07860 -3.41970 2.11860  H -3.53390 -6.17330 -2.47290  H -7.05410 -5.45100 0.99310  H -7.48550 -5.06730 -0.55050  H -6.19170 0.52240 1.13280  H -7.17530 -0.77260 0.52350  C 6.31660 -1.01820 0.86580  C 7.36480 -0.52850 1.85190  C 6.69850 -0.59900 -0.57800  O 5.81580 -1.24350 -1.49250  O 7.24820 0.20680 2.79960  O 8.61170 -1.02020 1.52050  N 6.17630 -2.48510 1.04420  H 5.32600 -0.54560 1.14840  H 6.64440 0.49650 -0.70960  H 7.70010 -0.97220 -0.86520  H 4.89750 -0.81120 -1.43550  H 9.31470 -0.74640 2.16560  H 5.40190 -2.80770 0.45610  H 7.01610 -2.96000 0.71320  C -1.87070 5.27230 -0.61450  C -3.34690 5.34680 -0.97050  C -1.02450 5.99860 -1.68870  O 0.35540 5.87760 -1.36350  O -4.13310 4.44350 -1.13520  O -3.75280 6.64800 -1.13390  N -1.71450 5.80470 0.76070  H -1.56850 4.17470 -0.58640  H -1.21020 5.58790 -2.69710  H -1.20110 7.09200 -1.69400  H 0.61160 4.91010 -1.32950  H -4.72380 6.72890 -1.33850  H -0.75040 5.65580 1.06710  H -1.87650 6.81190 0.77470 | C 0.68570 0.13090 0.83740  C 1.69050 1.02220 0.62110  H 2.61590 0.82770 1.20400  C 0.23850 -1.87380 -1.13980  C 0.80950 -0.99560 -2.04210  C 0.06260 0.04260 -2.61630  C -1.29470 0.21080 -2.24180  C -1.26670 0.88910 -0.58490  C -0.63610 -0.01400 0.30890  H -0.85820 -1.94420 -1.07280  H 1.88460 -1.06390 -2.27040  H 0.54840 0.77610 -3.25630  H -1.84500 -0.73850 -2.03740  H -1.25610 -0.78190 0.77870  C 0.92100 -3.00830 -0.53370  C 2.18310 -3.16080 -0.11130  H 0.26150 -3.89370 -0.45600  H 0.93770 -0.66320 1.57440  O 4.43850 -2.22960 -0.54430  C 1.81590 2.20050 -0.21350  C 3.21900 2.78280 -0.44880  C 4.30950 1.75900 -0.84680  C 3.81170 0.80490 -1.94460  C 4.90010 -0.16880 -2.41130  C 4.32720 -1.52290 -2.86110  C 4.71640 -2.65980 -1.91090  C 3.22130 -2.14060 0.08700  C 4.08320 -4.00950 -2.24630  C 4.78680 -4.67220 -3.42600  O 3.18370 -1.27000 0.93760  O 0.88400 2.83050 -0.71540  H 5.23320 2.30380 -1.15930  H 4.14710 -4.67180 -1.35700  H 3.00260 -3.90040 -2.46560  H 4.70510 -4.07600 -4.34240  H 4.34880 -5.65560 -3.64060  H 5.85440 -4.83320 -3.22960  H 4.69170 -1.76980 -3.87820  H 3.21920 -1.46700 -2.95840  H 5.49030 0.28460 -3.22880  H 5.62970 -0.32800 -1.57540  H 3.42620 1.38690 -2.80250  H 2.94180 0.23220 -1.55400  O 4.78250 1.02280 0.28630  H 4.07600 0.39110 0.63190  H 5.83160 -2.74500 -1.82000  H -0.71250 1.84220 -0.76660  C 3.63020 3.56960 0.79520  H 4.53730 4.15790 0.61020  H 2.84590 4.27370 1.11240  H 3.85470 2.90180 1.63870  H 3.12720 3.50380 -1.30590  H 2.51890 -4.14080 0.26380  C -2.20370 1.22620 -2.90550  C -3.28040 1.59360 -1.85520  C -2.76260 1.15010 -0.46920  H -1.65660 2.12680 -3.23480  H -2.68050 0.80960 -3.81340  H -3.56370 2.67040 -1.88550  H -2.94880 1.93660 0.29270  H -3.32070 0.25690 -0.12020  O -4.51260 0.93940 -2.17340  H -4.44810 -0.04860 -2.01850  C -10.24040 -3.17730 0.87830  C -8.89110 -2.83810 1.51810  C -8.16760 -1.83150 0.59850  C -6.76190 -1.51360 1.10700  C -5.97800 -0.70870 0.08650  O -5.03270 -1.18490 -0.54180  O -11.19010 -2.46890 0.65540  O -10.24860 -4.50010 0.51620  N -9.20870 -2.36400 2.88340  N -6.33510 0.61040 -0.10610  H -8.28270 -3.78370 1.60640  H -8.10440 -2.25420 -0.42890  H -8.76960 -0.90520 0.49870  H -6.79300 -0.98090 2.07740  H -6.19900 -2.45600 1.30010  H -11.11290 -4.78850 0.11410  H -8.36700 -2.25390 3.43920  H -9.70100 -1.47370 2.86840  H -5.82580 1.17510 -0.80900  H -7.10950 1.05080 0.35420  C 6.15060 -1.46180 2.28180  C 6.35010 -1.47300 3.78930  C 7.12130 -0.45120 1.61740  O 7.03860 -0.59320 0.20230  O 5.56040 -1.25090 4.67240  O 7.66360 -1.75660 4.10460  N 6.31130 -2.85540 1.79790  H 5.08040 -1.15110 2.07640  H 6.88580 0.58780 1.90830  H 8.17900 -0.68290 1.84760  H 6.15790 -0.20740 -0.12630  H 7.82030 -1.80550 5.08370  H 6.05090 -2.88610 0.80750  H 7.29000 -3.13630 1.85560  C -1.36150 4.47340 0.36770  C -2.84250 4.82980 0.31120  C -0.48190 5.74150 0.27540  O 0.89540 5.38260 0.31870  O -3.73700 4.26460 -0.27260  O -3.10820 5.96880 1.03190  N -1.19210 3.68050 1.60830  H -1.13290 3.79900 -0.51600  H -0.68900 6.31020 -0.64880  H -0.61030 6.40740 1.15180  H 1.10300 4.72330 -0.40380  H -4.07400 6.20630 1.04040  H -0.39460 3.05010 1.50450  H -0.99900 4.28690 2.40340 |

| **Structure IX** (Figure S10) | **Structure X** (Figure S10) |
| --- | --- |
| PM6 structure | PM6 structure |
| E(PM6) = -1.02283038 a.u. | E(PM6) = -0.31312808 a.u. |
| C -1.29940 1.02240 -0.83020  C -2.13100 1.50950 0.09480  H -3.20170 1.60950 -0.16820  C 0.49360 -0.72740 -1.33290  C 0.76610 -1.72100 -0.22640  C 1.59930 -1.38150 0.76730  C 2.11810 0.01910 0.72570  C 0.88880 0.96540 0.53060  C 0.17160 0.72480 -0.81380  H 0.30020 -2.70460 -0.31310  H 1.89440 -2.03330 1.57870  H 2.81810 0.11400 -0.15410  H 0.61950 1.43630 -1.56950  C -0.41350 -1.32880 -2.37490  C -1.63900 -1.85220 -2.29670  H 0.09440 -1.40400 -3.35180  H -1.74820 0.79370 -1.81630  O -3.09390 -2.84170 -0.52610  C -1.88380 2.00420 1.46170  C -2.54200 1.32200 2.66010  C -3.06270 -0.12490 2.49450  C -1.97780 -1.12850 2.07990  C -2.51420 -2.56640 2.13880  C -1.66980 -3.55130 1.31920  C -2.33610 -3.96960 0.00180  C -2.65820 -1.75680 -1.23700  C -1.37520 -4.56610 -1.02580  C -0.98130 -5.99140 -0.65660  O -3.34210 -0.76240 -1.06090  O -1.23870 3.02300 1.65840  H -3.54710 -0.43530 3.45560  H -1.85660 -4.55840 -2.02620  H -0.46400 -3.93310 -1.12260  H -0.45640 -6.03840 0.30470  H -0.31130 -6.42110 -1.41270  H -1.85400 -6.65360 -0.59000  H -1.48660 -4.46930 1.91500  H -0.66170 -3.12930 1.13230  H -2.56760 -2.90370 3.19120  H -3.57220 -2.57830 1.76970  H -1.08960 -1.02630 2.73070  H -1.62000 -0.89710 1.05540  O -4.17400 -0.17070 1.59060  H -3.87210 -0.13570 0.63560  H -3.20310 -4.65390 0.19630  H 0.16970 0.74520 1.35670  C -3.70150 2.22470 3.10200  H -4.07460 1.93910 4.09230  H -3.40070 3.27890 3.15650  H -4.54910 2.14490 2.40450  H -1.77130 1.30820 3.47740  H -2.07530 -2.33900 -3.18710  C 2.83480 0.61560 1.94150  C 2.69870 2.14430 1.71340  C 1.49400 2.35940 0.76530  H 2.36990 0.31290 2.89220  H 3.89150 0.31030 1.99310  H 2.62740 2.71900 2.65690  H 0.75690 3.05720 1.20420  H 1.82060 2.83630 -0.17840  O 3.89510 2.66110 1.11790  H 3.99810 2.30380 0.18460  H 1.49720 -0.59290 -1.85050  C 8.39760 -3.70540 -0.04590  C 8.34530 -2.38030 -0.81210  C 7.39070 -1.43130 -0.05710  C 7.18400 -0.11990 -0.81410  C 6.08740 0.71280 -0.17570  O 4.98030 0.85430 -0.68830  O 8.77780 -3.93660 1.07460  O 7.88140 -4.70320 -0.83310  N 9.75180 -1.93650 -0.92640  N 6.36560 1.31960 1.03680  H 7.95140 -2.57640 -1.85050  H 6.40960 -1.93560 0.08600  H 7.77410 -1.23860 0.96600  H 8.12370 0.46180 -0.88210  H 6.88340 -0.32430 -1.86800  H 7.91960 -5.59940 -0.40060  H 9.84010 -1.15270 -1.56470  H 10.13600 -1.66150 -0.02540  H 5.65040 1.92400 1.47340  H 7.26690 1.29490 1.47570  C -6.40130 -1.71850 -0.76140  C -7.52780 -1.14920 -1.61130  C -6.82100 -1.76380 0.73000  O -5.81290 -2.43690 1.47730  O -7.51960 -0.22020 -2.37880  O -8.70300 -1.83760 -1.38630  N -6.05170 -3.04300 -1.33050  H -5.50020 -1.04270 -0.88420  H -6.98630 -0.75120 1.13670  H -7.72550 -2.38460 0.88320  H -5.00220 -1.83670 1.58070  H -9.45080 -1.51420 -1.95350  H -5.17460 -3.35980 -0.90760  H -6.76490 -3.73010 -1.08840  C 0.07190 5.49200 -0.62160  C 0.87280 6.29050 -1.64190  C -1.03150 6.37760 0.01400  O -2.02960 5.56100 0.61590  O 2.06030 6.48730 -1.69440  O 0.03890 6.85170 -2.58380  N -0.44800 4.28570 -1.31220  H 0.79120 5.14820 0.17660  H -0.61280 7.07470 0.76000  H -1.60040 6.94410 -0.75270  H -1.63750 4.96900 1.31020  H 0.53420 7.35080 -3.28680  H -0.89600 3.67570 -0.61780  H -1.17530 4.55100 -1.97840 | C -0.88550 0.46730 1.79710  C -0.58040 1.66210 1.27380  H 0.09440 2.29590 1.88240  C -0.76210 -1.81270 0.68220  C -0.45180 -1.43180 -0.74540  C -1.49780 -1.17420 -1.54900  C -2.84490 -1.28820 -0.90310  C -2.84910 -0.40700 0.38790  C -1.68700 -0.72670 1.34470  H -1.44010 -2.72550 0.60440  H 0.59030 -1.39680 -1.05630  H -1.42820 -0.88790 -2.58910  H -2.98830 -2.36780 -0.61380  H -2.11710 -1.19160 2.27140  C 0.39890 -2.28840 1.50780  C 1.61100 -1.79450 1.76440  H 0.18020 -3.27930 1.94630  H -0.42730 0.25790 2.78430  O 3.36410 -0.34700 0.79000  C -0.93150 2.34140 0.00940  C 0.06900 3.35170 -0.56790  C 1.49880 2.80700 -0.82340  C 1.46850 1.36150 -1.34630  C 2.87250 0.85390 -1.69590  C 2.98770 -0.67540 -1.59410  C 3.79570 -1.11580 -0.36950  C 2.18900 -0.46810 1.48970  C 3.77990 -2.62460 -0.11530  C 4.71180 -3.35750 -1.07480  O 1.82380 0.56540 2.01530  O -1.99150 2.21660 -0.57770  H 2.01860 3.49110 -1.53760  H 4.09610 -2.81870 0.93070  H 2.75350 -3.03200 -0.20430  H 4.40780 -3.22910 -2.12040  H 4.71700 -4.43550 -0.86960  H 5.74810 -3.00820 -0.98630  H 3.47480 -1.07940 -2.50330  H 1.97590 -1.14220 -1.57880  H 3.15190 1.18810 -2.71150  H 3.60750 1.33500 -1.00820  H 0.80030 1.28850 -2.22420  H 1.01530 0.71020 -0.56980  O 2.31860 2.92690 0.33450  H 2.18000 2.17350 0.97300  H 4.84660 -0.72790 -0.42170  H -2.74960 0.66590 0.05830  C 0.11160 4.59070 0.32580  H 0.59150 5.42980 -0.19300  H -0.88970 4.91490 0.62810  H 0.71010 4.40620 1.23130  H -0.34540 3.65370 -1.56860  H 2.31100 -2.39180 2.37220  C -4.13150 -0.84350 -1.61020  C -5.12010 -0.61990 -0.43080  C -4.29590 -0.56750 0.88130  H -3.98090 0.08980 -2.17970  H -4.50080 -1.59910 -2.31770  H -5.75220 0.27560 -0.58100  H -4.61190 0.26120 1.53170  H -4.45470 -1.49980 1.45470  O -5.98680 -1.75880 -0.26440  H -6.54860 -1.88820 -1.04770 |

| **Isolated Ser amino acid** | **Isolated Gln amino acid** |
| --- | --- |
| PM6 structure | PM6 structure |
| E(PM6) = -0.21792345 a.u. | E(PM6) = -0.22963813 a.u. |
| C -0.23080 0.13900 -0.48980  C 1.22850 -0.15620 -0.16540  C -1.15120 -0.74880 0.38500  O -2.51130 -0.41750 0.10740  O 2.07820 -0.64220 -0.86630  O 1.50600 0.17590 1.14090  N -0.46090 1.59420 -0.32640  H -0.97130 -1.82470 0.22240  H -1.04580 -0.51400 1.46440  H -2.78400 -0.77200 -0.76580  H 2.46230 0.03460 1.37980  H -1.44470 1.80380 -0.50290  H -0.26170 1.88400 0.63150  H -0.39110 -0.10460 -1.57940 | C 2.36470 -0.24290 0.23910  C 1.16070 0.20110 -0.59130  C -0.12150 -0.07750 0.21860  C -1.36290 -0.07890 -0.67700  C -2.61680 -0.01770 0.17710  O -2.72210 0.64350 1.19490  O 2.88250 0.26610 1.20020  O 2.83680 -1.43460 -0.25980  N 1.39950 1.62090 -0.94600  N -3.71020 -0.75120 -0.28410  H 1.14880 -0.39270 -1.55020  H -0.04250 -1.05190 0.74410  H -0.24240 0.67520 1.03210  H -1.36760 0.80720 -1.34830  H -1.34940 -0.96110 -1.34630  H 3.63420 -1.77140 0.23090  H 0.61820 1.99660 -1.47540  H 1.51060 2.19780 -0.11190  H -4.57100 -0.74550 0.24350  H -3.68540 -1.34690 -1.08930 |
