# Supplementary material for: Computational Study of a Model System of Enzyme-Mediated [4+2] Cycloaddition Reaction
Source: PLoS One. 2015 Apr 8;10(4):e0119984. doi: 10.1371/journal.pone.0119984 (PMC4390235; doi:10.1371/journal.pone.0119984)
Supplement: S1 Table — B3LYP/6-311+G(d) level (bond length in Å, angles in deg); for atom numbering see S1–S4 Figs. (DOC) [file pone.0119984.s012.doc]

**Table S1.** **Optimized geometry parameters of molecular structures 1-15.**

|  | **(a)** | | | **(b)** | | | **(c)** | | | **(d)** | | | **(e)** | | |
| --- | --- | --- | --- | --- | --- | --- | --- | --- | --- | --- | --- | --- | --- | --- | --- |
|  | **1** | **2-TS^a^** | **3** | **4** | **5-TS^a^** | **6** | **7** | **8-TS^a^** | **9** | **10** | **11-TS^a^** | **12** | **13** | **14-TS^a^** | **15** |
| C(4)-C(5) | 1.338 | 1.383 | 1.507 | 1.337 | 1.387 | 1.513 | 1.337 | 1.388 | 1.513 | 1.350 | 1.389 | 1.522 | 1.352 | 1.386 | 1.520 |
| C(5)-C(6) | 1.456 | 1.404 | 1.335 | 1.468 | 1.403 | 1.337 | 1.468 | 1.403 | 1.337 | 1.460 | 1.395 | 1.337 | 1.459 | 1.396 | 1.335 |
| C(6)-C(7) | 1.338 | 1.383 | 1.507 | 1.340 | 1.386 | 1.503 | 1.340 | 1.386 | 1.503 | 1.342 | 1.411 | 1.499 | 1.344 | 1.417 | 1.499 |
| C(11)-C(12) | 1.328 | 1.387 | 1.553 | 1.332 | 1.391 | 1.525 | 1.332 | 1.391 | 1.524 | 1.344 | 1.418 | 1.539 | 1.344 | 1.419 | 1.531 |
| **C(4)-C(12)** | − | **2.256** | **1.548** | **5.239** | **2.266** | **1.545** | **4.886** | **2.262** | **1.545** | **4.776** | **2.802** | **1.572** | **4.959** | **3.075** | **1.583** |
| **C(7)-C(11)** | − | **2.256** | **1.548** | **3.326** | **2.189** | **1.535** | **3.266** | **2.190** | **1.535** | **3.111** | **1.941** | **1.534** | **3.158** | **1.885** | **1.540** |
| C(4)-C(5)-C(6) | 124.4 | 122.0 | 119.1 | 126.5 | 122.1 | 124.4 | 126.5 | 122.1 | 124.5 | 126.8 | 123.6 | 125.4 | 126.7 | 126.4 | 124.7 |
| C(5)-C(6)-C(7) | 124.4 | 122.0 | 119.1 | 126.1 | 120.3 | 121.1 | 126.2 | 120.2 | 121.0 | 126.6 | 122.1 | 121.4 | 125.9 | 124.1 | 121.5 |
| C(6)-C(7)-C(11) | − | 102.2 | 111.4 | 144.7 | 102.0 | 111.3 | 134.3 | 101.9 | 111.1 | 108.2 | 108.7 | 111.0 | 132.8 | 107.0 | 112.3 |
| C(7)-C(11)-C(12) | − | 109.2 | 113.8 | 105.8 | 107.5 | 110.6 | 105.0 | 107.3 | 110.6 | 121.7 | 113.1 | 112.4 | 106.4 | 111.4 | 109.5 |
| C(11)-C(12)-C(4) | − | 109.2 | 113.8 | 73.0 | 108.8 | 109.5 | 78.2 | 108.8 | 109.3 | 70.5 | 100.9 | 108.1 | 79.7 | 97.4 | 109.1 |

B3LYP/6-311+G(d) level (bond length in Å, angles in deg); for atom numbering see Figures S1-S4.

^a^Imaginary frequency for the transition state: 534.7 *i* cm^-1^ (**2-TS**), 531.2 *i* cm^-1^ (**5-TS**), 532.7 *i* cm^-1^ (**8-TS**), 434.6 *i* cm^-1^ (**11-TS**), 384.6 *i* cm^-1^ (**14-TS**).
