# Supplementary material for: Computational Study of a Model System of Enzyme-Mediated [4+2] Cycloaddition Reaction
Source: PLoS One. 2015 Apr 8;10(4):e0119984. doi: 10.1371/journal.pone.0119984 (PMC4390235; doi:10.1371/journal.pone.0119984)
Supplement: S2 Table — PM6 and B3LYP/6-311+G(d) levels of theory (see Fig. 2 for atomic numbers). (DOC) [file pone.0119984.s013.doc]

**Table S2.** **Mulliken atomic charges** **for molecules in reactions (a)-(c).**

|  | C(4) | C(5) | C(6) | C(7) | C(11) | C(12) |
| --- | --- | --- | --- | --- | --- | --- |
| **(a)** | PM6 | | | | | |
| 1 | -0.337 | -0.108 | -0.108 | -0.337 | -0.286 | -0.286 |
| 2-TS | -0.268 | -0.154 | -0.154 | -0.268 | -0.280 | -0.280 |
|  | B3LYP/6-311+G(d) | | | | | |
| 1 | -0.629 | -0.028 | -0.028 | -0.629 | -0.428 | -0.428 |
| 2-TS | -0.535 | -0.118 | -0.118 | -0.535 | -0.523 | -0.523 |
| **(b)** | PM6 | | | | | |
| 4 | -0.357 | -0.078 | -0.224 | -0.096 | -0.058 | -0.396 |
| 5-TS | -0.273 | -0.155 | -0.215 | -0.064 | -0.085 | -0.343 |
|  | B3LYP/6-311+G(d) | | | | | |
| 4 | -0.640 | -0.157 | -0.518 | +0.349 | +0.289 | -0.731 |
| 5-TS | -0.539 | -0.100 | -0.442 | +0.498 | +0.415 | -1.091 |
| **(c)** | PM6 | | | | | |
| 7 | -0.343 | -0.095 | -0.195 | -0.108 | -0.055 | -0.379 |
| 8-TS | -0.270 | -0.153 | -0.208 | -0.064 | -0.081 | -0.336 |
|  | B3LYP/6-311+G(d) | | | | | |
| 7 | -0.642 | -0.136 | -0.576 | +0.514 | +0.260 | -0.712 |
| 8-TS | -0.537 | -0.105 | -0.457 | +0.519 | +0.510 | -1.051 |

PM6 and B3LYP/6-311+G(d) levels of theory (see Figure 2 for atomic numbers).
