# Supplementary material for: Computational Study of a Model System of Enzyme-Mediated [4+2] Cycloaddition Reaction
Source: PLoS One. 2015 Apr 8;10(4):e0119984. doi: 10.1371/journal.pone.0119984 (PMC4390235; doi:10.1371/journal.pone.0119984)
Supplement: S3 Table — PM6 and B3LYP/6-311+G(d) levels of theory (see Fig. 2 for atomic numbers). (PDF) [file pone.0119984.s014.pdf]

Table S3. Mulliken atomic charges for molecules in reaction (d).

	C(4)	C(5)	C(6)	C(7)	C(11)	C(12)	C(13)	C(14)
	PM6							
10	-0.172	-0.135	-0.203	-0.101	-0.115	-0.198	-0.084	-0.354
11-TS	-0.129	-0.163	-0.221	-0.044	-0.093	-0.188	-0.093	-0.353
	B3LYP/6-311+G(d)							
10	+0.189	-0.750	-0.453	+0.631	+0.348	-0.694	+0.224	-0.795
11-TS	+0.387	-1.081	-0.144	+0.372	-0.066	+0.015	-0.221	-0.673

PM6 and B3LYP/6-311+G(d) levels of theory (see Figure 2 for atomic numbers).
