# Supplementary material for: Computational Study of a Model System of Enzyme-Mediated [4+2] Cycloaddition Reaction
Source: PLoS One. 2015 Apr 8;10(4):e0119984. doi: 10.1371/journal.pone.0119984 (PMC4390235; doi:10.1371/journal.pone.0119984)
Supplement: S4 Table — PM6 and B3LYP/6-311+G(d) levels of theory (see Fig. 2 for atomic numbers). (DOC) [file pone.0119984.s015.doc]

**Table S4.** **Mulliken atomic charges** **for molecules in reaction (e).**

|  | C(4) | C(5) | C(6) | C(7) | C(10) | C(11) | C(12) | C(13) | C(14) | C(15) | O(at C(15)) |
| --- | --- | --- | --- | --- | --- | --- | --- | --- | --- | --- | --- |
|  | PM6 | | | | | | | | | | |
| 13 | -0.238 | -0.078 | -0.232 | -0.054 | -0.358 | -0.015 | -0.282 | +0.072 | -0.432 | +0.591 | -0.552 |
| 14-TS | -0.232 | -0.109 | -0.214 | -0.011 | -0.333 | -0.106 | -0.182 | +0.034 | -0.423 | +0.577 | -0.551 |
|  | B3LYP/6-311+G(d) | | | | | | | | | | |
| 13 | +0.802 | -1.067 | -0.311 | +0.782 | -1.065 | +0.876 | -0.418 | -0.526 | +0.107 | -0.127 | -0.311 |
| 14-TS | -0.065 | -0.620 | -0.734 | +0.515 | -0.423 | +0.359 | -0.614 | -0.094 | -0.045 | -0.303 | -0.281 |

PM6 and B3LYP/6-311+G(d) levels of theory (see Figure 2 for atomic numbers).
