# Supplementary material for: Computational Study of a Model System of Enzyme-Mediated [4+2] Cycloaddition Reaction
Source: PLoS One. 2015 Apr 8;10(4):e0119984. doi: 10.1371/journal.pone.0119984 (PMC4390235; doi:10.1371/journal.pone.0119984)
Supplement: S5 Table — PM6 level (bond length in Å, angles in deg); for atom numbering see Figs. S5–S8. (DOC) [file pone.0119984.s016.doc]

**Table S5.** **Optimized geometry parameters of molecular structures 16-27.**

|  | **+Gln** | | | **+Gln+Ser** | | | **+Gln+2Ser** | | | **+8H_2_O** | | |
| --- | --- | --- | --- | --- | --- | --- | --- | --- | --- | --- | --- | --- |
|  | **16** | **17-TS^a^** | **18** | **19** | **20-TS^a^** | **21** | **22** | **23-TS^a^** | **24** | **25** | **26-TS^a^** | **27** |
| C(4)-C(5) | 1.340 | 1.384 | 1.512 | 1.340 | 1.384 | 1.512 | 1.341 | 1.382 | 1.512 | 1.341 | 1.379 | 1.511 |
| C(5)-C(6) | 1.462 | 1.401 | 1.341 | 1.461 | 1.401 | 1.341 | 1.460 | 1.402 | 1.340 | 1.460 | 1.405 | 1.341 |
| C(6)-C(7) | 1.339 | 1.416 | 1.497 | 1.339 | 1.416 | 1.496 | 1.342 | 1.418 | 1.494 | 1.339 | 1.415 | 1.495 |
| C(11)-C(12) | 1.340 | 1.413 | 1.542 | 1.341 | 1.414 | 1.541 | 1.341 | 1.419 | 1.543 | 1.340 | 1.419 | 1.541 |
| **C(4)-C(12)** | **3.827** | **2.503** | **1.575** | **3.905** | **2.504** | **1.575** | **3.856** | **2.514** | **1.575** | **3.784** | **2.575** | **1.574** |
| **C(7)-C(11)** | **3.208** | **1.808** | **1.564** | **3.182** | **1.806** | **1.564** | **3.084** | **1.791** | **1.564** | **3.228** | **1.779** | **1.564** |
| C(4)-C(5)-C(6) | 123.2 | 121.2 | 119.0 | 123.1 | 121.3 | 119.1 | 124.2 | 121.2 | 119.2 | 124.4 | 121.5 | 118.9 |
| C(5)-C(6)-C(7) | 125.0 | 118.9 | 115.8 | 124.9 | 119.1 | 115.8 | 124.5 | 119.3 | 115.6 | 124.5 | 119.0 | 115.3 |
| C(6)-C(7)-C(11) | 88.2 | 106.2 | 106.5 | 95.8 | 106.0 | 107.0 | 112.0 | 105.9 | 107.3 | 94.2 | 106.7 | 106.7 |
| C(7)-C(11)-C(12) | 99.5 | 110.4 | 112.7 | 98.6 | 110.3 | 112.7 | 89.3 | 110.4 | 112.3 | 93.1 | 110.4 | 111.9 |
| C(11)-C(12)-C(4) | 107.5 | 105.4 | 109.3 | 107.4 | 105.5 | 109.5 | 115.9 | 105.2 | 109.6 | 113.4 | 104.3 | 109.4 |

PM6 level (bond length in Å, angles in deg); for atom numbering see Figures S5-S8.

^a^Imaginary frequency for the transition state: 719.5 *i* cm^-1^ (**17-TS**), 713.5 *i* cm^-1^ (**20-TS**), 678.3 *i* cm^-1^ (**23-TS**), 637.3 *i* cm^-1^ (**26-TS**).
